# Magnetic‐optical dual functional Janus particles for the detection of metal ions assisted by machine learning

**DOI:** 10.1002/smo.20230006

**Published:** 2023-09-19

**Authors:** Jianhang Liu, Yingdi Lv, Xinghai Li, Shi Feng, Wenbo Yang, Yumeng Zhou, Shengyang Tao

**Affiliations:** ^1^ State Key Laboratory of Fine Chemicals Frontier Science Center for Smart Materials School of Chemistry Dalian University of Technology Dalian China; ^2^ School of Chemistry and Chemical Engineering Northwestern Polytechnical University Xi'an China; ^3^ Xi'an Modern Chemistry Research Institute Xi'an China; ^4^ Instrumental Analysis Center Dalian University of Technology Dalian China

**Keywords:** functional microspheres, machine learning, metal ions detection, microfluidics, principal component analysis

## Abstract

Functional polymer microspheres have broad application prospects in various fields, such as metal ion detection, adsorption, separation, and controlled drug release. However, integrating different functions in a single microsphere system is a significant challenge in this field. In this work, we prepared multicompartmental emulsion droplets utilizing microfluidic technology. Fe_3_O_4_ magnetic nanoparticles were added to one of the compartments of the emulsion droplets as functional particles, and Janus microspheres were obtained after curing. Fluorescent probes enter the two compartments of the Janus microspheres by diffusion. The fluorescence changes of the microspheres were observed in situ and captured through a fluorescence microscope. The images are processed by image recognition software and a Python program. The “fingerprint” of the detected metal ions is obtained by dimensionality reduction of the data through Principal Component Analysis. We employ different algorithms to build Machine Learning models for predicting the metal ion species and concentration. The variation of fluorescence intensity of the three fluorescent probes and the corresponding R, G, and B channel values and time are used as descriptors. The results show that the Random Forest, K‐neighborhood (KNN), and Neural Network models demonstrated a better predicted effect with a variance (R^2^) greater than 0.9 and a smaller root mean square error; among them, the KNN model predicted the most accurate results.

## Graphical abstract

A composite functional microsphere coupled with magnetic control and metal ion detection is prepared using the droplet template method. PCA is used to downscale the data to obtain a “fingerprint” of the metal ions. The RF, KNN and NN models have good predictions for the concentrations and species of metal ions.

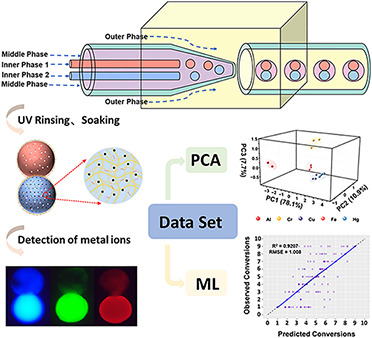

## 1 INTRODUCTION

Functional polymer microspheres are materials with a particle size distribution between 1 and 1000 μm and spherical or sphere‐like shapes with unique structural and functional properties.^[1]^ Due to these properties, they have been widely used in many fields. Functional polymer microspheres can be classified into porous microspheres, microcapsules, and compartment‐structured microspheres. Porous microspheres find extensive applications in areas such as adsorption, separation, and catalysis.[^2^, ^3^, ^4^] Microcapsules are widely utilized in pharmaceutical controlled release and encapsulation of active substances.[^5^, ^6^, ^7^, ^8^, ^9^] Compartment‐structured microspheres can serve as microreactors.[^10^, ^11^, ^12^] These multicompartmental microspheres can also achieve composite functions by adding different functional components to the compartments, such as magnetic particles and luminescent molecules.[^13^, ^14^]

Functional microspheres can be synthesized through various methods such as emulsion polymerization, spray drying, phase separation, and layer self‐assembly. However, the functional microspheres obtained by these preparation methods often have a broad particle size distribution and poor monodispersity. Functional microspheres synthesized by droplet templating method utilizing microfluidic technology provide significant advantages, such as controllable nested structure, uniform droplet size, good monodispersity, and adjustable components.^[15]^

Microfluidics is an emerging technology that utilizes microfluidic manipulation as its core and the microchannel network as its structural feature.^[16]^ It has shown a wide range of applications in many fields such as material preparation,[^17^, ^18^, ^19^] catalytic synthesis,[^20^, ^21^] and disease diagnosis.^[22]^ Droplet templating method is a technique that utilizes microfluidic technology to synthesize microemulsion droplets as templates for the production of functional microspheres. Microemulsion droplets are composed of two or more mutually incompatible liquids, with the dispersed phase distributed as droplets in the continuous phase. Double emulsion droplets are highly structured fluids with smaller droplets wrapped in the droplets of the dispersed phase. Microfluidics provides a new approach to prepare multi‐component double emulsion droplets, where the encapsulated droplets' composition, number, and size can be precisely and independently controlled. Microemulsion droplets can be solidified through various methods, including polymerization reactions, cross‐linking reactions, phase separation, and self‐assembly.[^23^, ^24^, ^25^, ^26^] Polymerization is the most widely used method due to its broad applicability, high curing efficiency, and numerous polymeric materials available. The conversion process can typically be achieved by adding a photosensitive prepolymer into the dispersed phase, with a small amount of initiator, under heating or UV irradiation conditions.

Multicompartment structured double emulsion droplets containing two independent compartments can be utilized to prepare Janus‐structured microspheres, and different functional materials can be added to obtain multifunctional microspheres with unique physicochemical properties. Due to these properties, Janus‐structured microspheres have wide applications in drug delivery,^[27]^ micromotor,^[28]^ optical anti‐counterfeiting,^[29]^ and other fields. For example, researchers produced Janus microspheres containing Fe_3_O_4_ magnetic nanoparticles and Ag nanoparticles using the droplet templating method. The Ag nanoparticles catalyzed the decomposition of H_2_O_2_ and generated oxygen, providing a propulsive effect to the microsphere and promoting mass transfer in solution, thereby facilitating the adsorption of organic pollutants or heavy metal ions.

**FIGURE 1:**
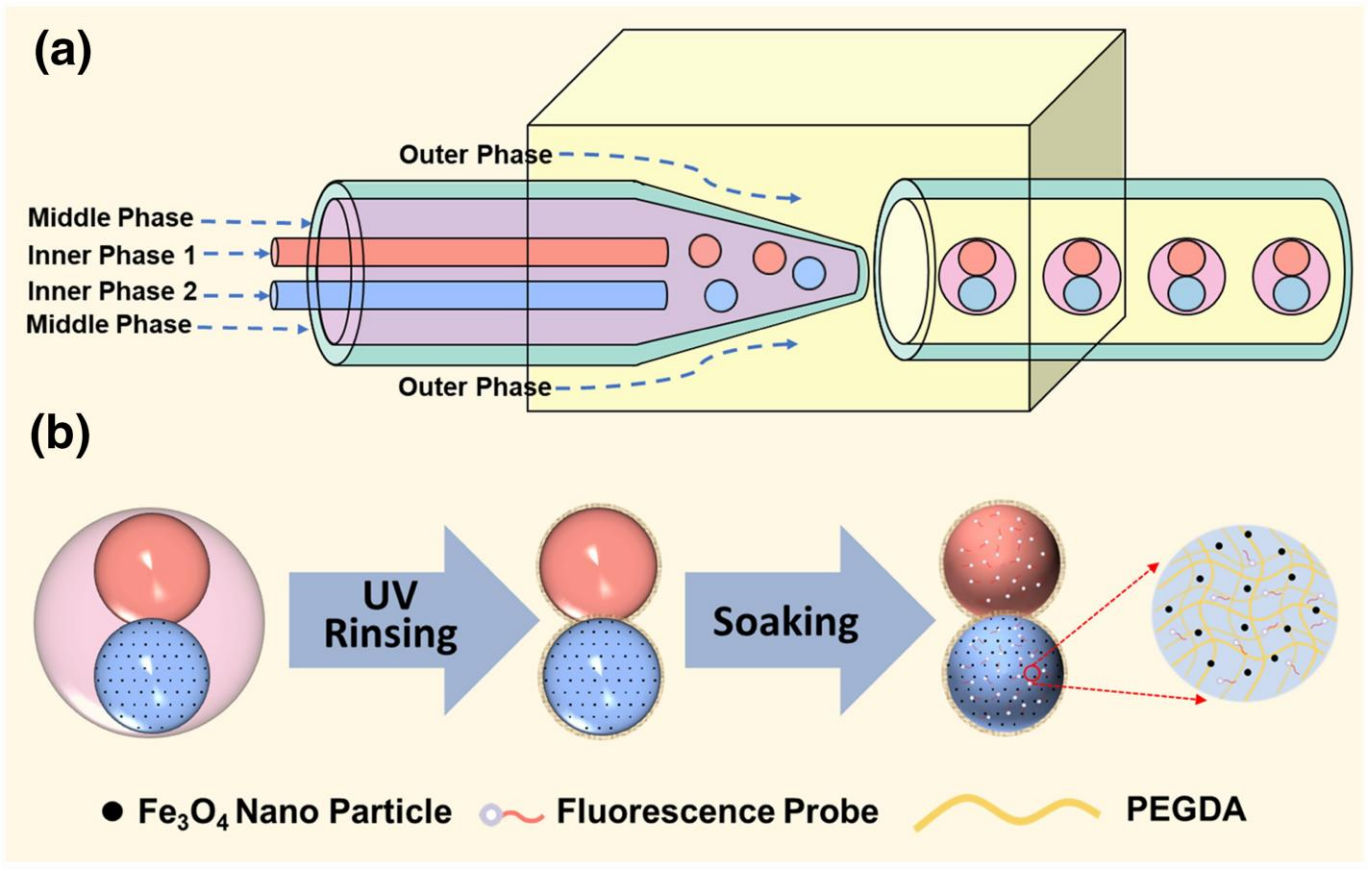
Schematic diagram of the preparation process of composite functional microspheres using droplet templating method. (a) Double emulsion droplets prepared by microfluidic chip; (b) composite functional microspheres prepared using microemulsion droplets as templates.

Pollution caused by metal ions remains one of the most acute problems facing humans and poses a significant threat to human health. Therefore, developing sensors to detect metal ions in water has garnered interest. Using the droplet templating method, we prepared Janus microspheres with magnetic particles and fluorescent probes as functional particles, thus realizing the microspheres' magnetic control and metal ion detection functions. The preparation process of composite functional microspheres using the droplet templating method is shown in Figure 1. During the metal ion detection, the functional microspheres were observed and photographed using fluorescence microscopy, and the changes in Mean Fluorescence Intensity (MFI) were extracted by automating the analysis of the captured images using image analysis software.^[30]^ Using Principal Component Analysis (PCA), data dimensionality reduction was applied to a large dataset with multiple variables to extract the Principal Component (PC) variables that could cover as much information from the data as possible.^[31]^ Characteristic curves of the metal ions were then drawn. At the same time, Machine Learning (ML) research has developed rapidly in recent years due to the improvement of computing power, optimization of algorithms, and the explosive growth of data in the era of Big Data. Researchers have already combined microfluidics with ML to design and control microfluidic devices,[^32^, ^33^, ^34^] and automated generation of droplets.[^35^, ^36^] Thus, we utilized the variable values obtained from the image analysis software as the ground truth to construct a model that could predict the species and concentration of metal ions in the solution. Popular algorithms such as Linear Regression (LR) model, Bayesian (Bys) model, K‐neighborhood (KNN) model, Support Vector Machine (SVM) model, Neural Network (NN) model, and Random Forest (RF) model were used to construct and optimize the model to increase the accuracy of the prediction results. Finally, we imported experimental data and obtained an ML model with R^2^ greater than 0.9 for qualitative and semi‐quantitative detection of various metal ions in solution.

## 2 EXPERIMENTAL SECTION

### 2.1 Material

All reagents were used without purification, and deionized water was used during the experiments. Polyvinyl alcohol (PVA; Mw ≈ 67,000), octadecyl trimethoxy silane, and 7‐diethylamino‐4‐methylcoumarin (MDAC) were purchased from Aladdin Chemical. 2‐Hydroxy‐2‐methylpropiophenone, ferric nitrate, chromium nitrate, and Fe_3_O_4_ nanoparticles (200 nm) were purchased from Macklin Biochemical. Rhodamine B and poly (ethylene glycol) diacrylate (PEGDA; Mn = 700) were purchased from Sigma‐Aldrich. The surfactant EM 90 was purchased from Evonik Industries. Liquid paraffin and anhydrous ethanol were purchased from Kemiou Chemical Reagent Co. Ltd. (Tianjin). Mercuric nitrate was purchased from Xiya Chemical Technology Co. Ltd. (Shandong). Aluminum nitrate was purchased from Xilong Scientific Co. Ltd. (Sichuan). Cupric nitrate was purchased from Bodi Chemical Co. Ltd. (Tianjin). Hydrazine hydrate was purchased from Guangfu Fine Chemical Co. Ltd. (Tianjin). Calcein was purchased from Fuchen Chemical Reagent Co. Ltd. (Tianjin).

### 2.2 Preparation of microfluidic chips

A capillary microfluidic chip was fabricated to produce microemulsion. First, a round borosilicate glass capillary tube (outer diameter: 1 mm, inner diameter: 0.58 mm) was stretched into a conical capillary tube by Micropipette Puller (Sutter Instrument, P‐1000), and the inner diameter of the conical part was polished to 190 μm using a Forging Needle Instrument (Narishige, MF‐900) and sandpaper. The conical tip was rinsed with ethanol, blown dry, and repeatedly rinsed until the capillary tube was without glass residue to ensure it was flush. Collection tubes were made directly from round capillary glass tubes. Two finer capillary glass tubes with an inner diameter of approximately 100 μm were inserted into the injection tube, which was stretched from the round capillary glass tubes using a flame gun.

Next, the capillary tubes were surface‐modified. This work synthesized a (W1+W2)/O/W double emulsion droplet, and two smaller aqueous phase droplets were presented in the middle phase. Therefore, all capillary glass tubes were modified to hydrophilic wettability by oxygen plasma, and then the injection tubes were hydrophobically modified through chemical vapor deposition and treated with octadecyl trimethoxy silane as a hydrophobic agent at 120°C for 6 h. After surface modification of the glass tube, the chip assembly was started by fixing the square glass tube on the glass slide to ensure the coaxiality of the injection tube and the collection tube, and the calibration was completed under the microscope. After the assembly, the joint was fixed with epoxy resin to ensure the chip was sealed without leakage.

### 2.3 The production of (W1+W2)/O/W double emulsion droplets

The solvent system was determined as follows: the outermost phase fluid was 5 wt% PVA solution; the middle phase fluid was liquid paraffin with an appropriate amount of EM 90 as a surfactant; the innermost phase fluid was PEGDA with 2‐hydroxy‐2‐methylpropiophenone as photoinitiator, the microfluidic chip and micro‐syringe pump (Longer, Lsp‐02A) were connected through a Polytetrafluoroethylene tube and a disposable syringe. Flow rate control was achieved by the micro‐syringe pump. During the emulsion preparation, sequential injections of the outermost phase, middle phase, and innermost phase fluids were performed. Utilizing a high‐speed camera, the flow behavior of a three‐phase fluid was observed, and by adjusting the flow rate, the generation of microemulsion droplets containing two compartments was observed, and ultimately double emulsion droplets were collected. Based on the experimental experience, to generate (W1+W2)/O/W double emulsion droplets, the flow rates of the outermost phase, middle phase, and innermost phase fluids were precisely controlled at 5 mL/h, 1.5 mL/h, and 0.2 mL/h, respectively. The preparation process of microemulsion droplets is schematically shown in Figure 1a.

### 2.4 Synthesis of Janus‐structured functional microspheres containing fluorescent probes

The microemulsion droplets were irradiated with UV light, and the light‐curing resin rapidly polymerized under the action of a photoinitiator to obtain Janus‐structured functional microspheres. The resulting microspheres were washed several times with anhydrous ethanol and deionized water to remove residual PVA aqueous solution. Fluorescent probes were not added to the innermost phase during the microemulsion droplet preparation as it would affect UV curing. Instead, the fluorescent probes were loaded into the whole Janus particles after curing by soaking using the diffusion principle. The preparation process of functional microspheres is schematically shown in Figure 1b.

Three cheap and easily available molecules, namely Rhodamine B Hydrazide (RBH), Calcein, and MDAC, were selected for the fluorescent probes. Rhodamine B Hydrazide was synthesized in the laboratory using the following method: Rhodamine B (2.0 g) was dissolved in anhydrous ethanol (80.00 mL), stirred well, and then 85% hydrazine hydrate (8.0 mL) was added slowly. The mixture was heated and stirred to reflux for 6 h. The obtained product was firstly distilled under reduced pressure and then recrystallized from methanol and water to obtain a pink crystalline product which was identified as RBH.^[13]^ The IR and NMR spectra of RBH are shown in Figure S1 and Figure S2.

### 2.5 Detection of metal ions by functional microspheres

A magnetic field immobilized the functional microspheres in microchannels assembled by square glass tubes and slides. Different species and concentrations of metal ions, including Al^3+^, Cr^3+^, Fe^3+^, Cu^2+^, and Hg^2+^, were injected using a syringe pump. Aqueous solutions of metal ions with concentrations of 1 × 10^−3^, 5 × 10^−4^, and 2 × 10^−4^ mol/L, respectively, were prepared. The functional microspheres were observed in situ using a fluorescence microscope (Nikon ECLIPSE Ti‐2), and different detection conditions were set for the three types of functional microspheres. The exposure time was 1 ms for the RBH microspheres with an excitation wavelength of illuminant range from 540 to 580 nm; the exposure time was 50 ms for the Calcein microspheres with an excitation wavelength of illuminant range from 465 to 495 nm; and the exposure time was 15 ms for the MDAC microspheres with the excitation wavelength of illuminant range from 361 to 389 nm. During the assay, a timed image capture program was set up, and the image was captured every 10 s for a total of 61 images. Microsphere images were obtained to provide data for subsequent PCA downscaling and ML model building.

### 2.6 Instrument characterization

The microemulsion droplet preparation process was captured using a Phantom Miro C210 high‐speed camera. Structural and morphological information of the microspheres was obtained using a scanning electron microscope (SEM, QUANTA 450, and NOVA Nano SEM 450) with an operating voltage of 30 kV. The fluorescence emission curves of the fluorescent probes were obtained using a fluorescence spectrophotometer (Agilent G9800A).

## 3 RESULTS AND DISCUSSION

### 3.1 Preparation and characterization of functional microspheres

**FIGURE 2:**
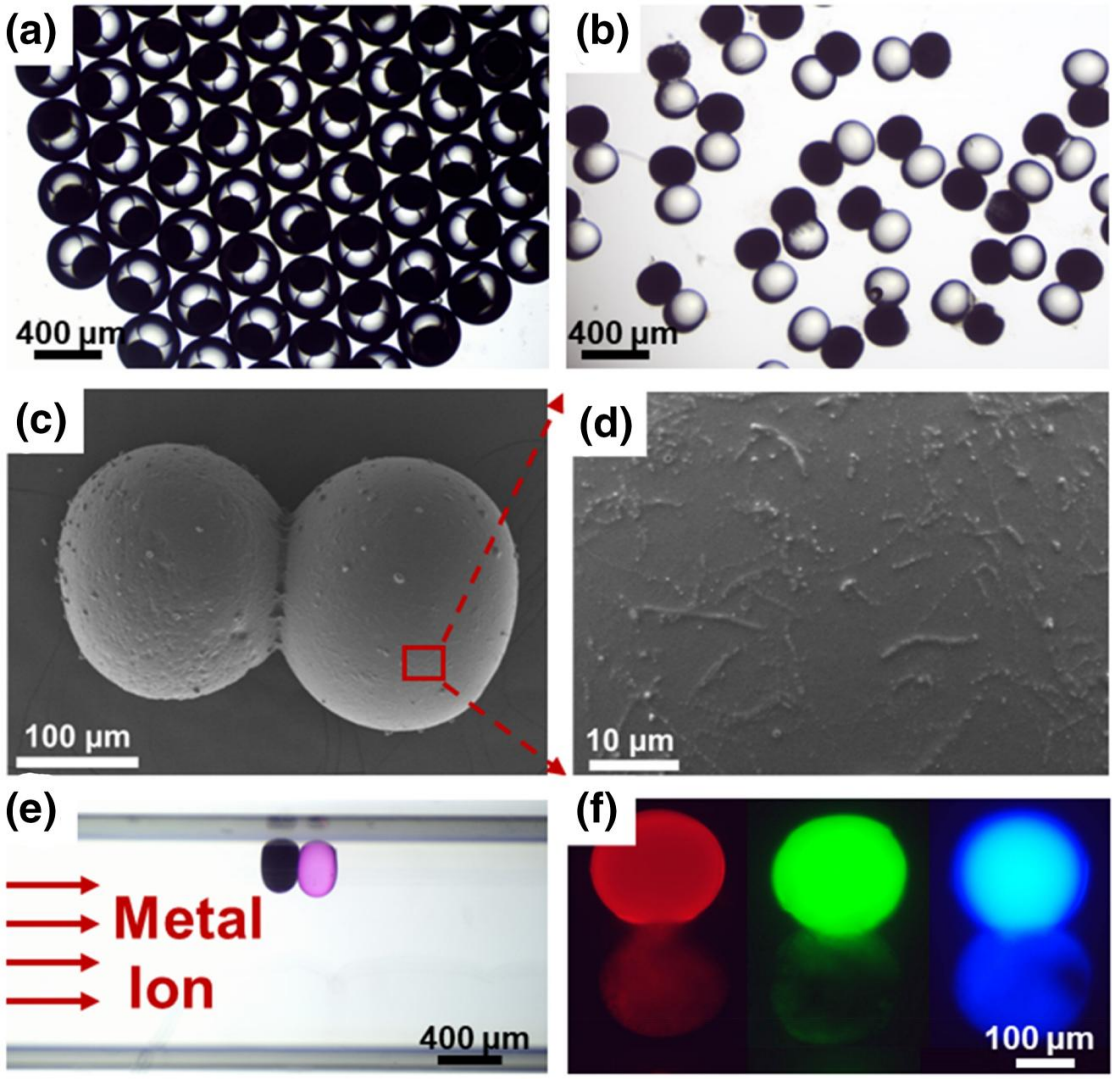
Images of functional microspheres. (a) and (b) are optical microscope images of microemulsion droplets and functional microspheres; (c) and (d) are SEM images of functional microsphere and its surface morphology after magnification; (e) the optical microscope image of the process of detecting metal ions by functional microspheres; (f) images of three types of functional microspheres under a fluorescence microscope. The scale is shown in the figure.

The preparation of double emulsion droplets and composite functional microspheres is illustrated in Figure 1. The generation of double‐emulsion droplets was achieved by controlling the flow rate of the three‐phase fluid, and the images of the double emulsion droplets under the microscope are shown in Figure 2a. Image J software was utilized to measure the average diameter of the double emulsion droplets, which was found to be 405.8 μm, and the Coefficient of Variation was calculated to be 0.927%, indicating that the particle size distribution obtained by microfluidic technology was highly uniform. The functional microspheres with metal ion detection and magnetic control functions were obtained after UV irradiation, rinsing and soaking by fluorescent probe solution, and the images of the functional microspheres under the microscope are shown in Figure 2b.

The SEM image of the functional microsphere and its surface morphology are shown in Figure 2c,d, respectively. The surface of the microspheres that formed after the photopolymerization was slightly rough. The microspheres were immobilized in a microchannel using a magnetic field to detect metal ions, and the channel was injected with a metal ion solution. After interacting with RBH and Hg^2+^, the functional microspheres exhibited a color change under the microscope, as shown in Figure 2e. The three fluorescent probes, including RBH, Calcein, and MDAC, were observed under the fluorescence microscope and are displayed in Figure 2f. Rhodamine B Hydrazide is a fluorescent‐enhanced probe that emits fluorescence after reacting with metal ions to complete the ring‐opening reaction. Calcein and MDAC are quenching fluorescent probes, and their fluorescence intensity decreases after reacting with metal ions.

### 3.2 Exploration of the optimal conditions for the use of fluorescent probes

The optimal use concentration of the three fluorescent probes was explored after selection. Different concentrations of fluorescent probe solutions were configured to detect Hg(NO_3_)_2_ solution. Fluorescence emission spectroscopy was performed after mixing. After the ring‐opening reaction of RBH with Hg^2+^, we found that the maximum emission wavelength of the fluorescence emission spectrum was 586 nm. The fluorescence emission spectra of different concentrations of RBH were tested separately, and it was found that within a specific range, the higher the concentration of RBH, the greater the fluorescence intensity. Therefore, experiments were conducted by soaking the functional microspheres with 10^−3^ mol/L RBH. The fluorescence emission spectra of different concentrations of Calcein and MDAC were also characterized, and the optimal concentrations of Calcein and MDAC were determined to be 10^−4^ mol/L when a strong fluorescence emission signal could be obtained without obvious self‐quenching. The curves are shown in Figure S3.

Next, 10 metal ions, namely Al^3+^, Ca^2+^, Cr^3+^, Fe^3+^, Cu^2+^, Zn^2+^, Cd^2+^, Ba^2+^, Hg^2+^, and Pb^2+^, were initially selected for detection through fluorescent probes at a concentration of 10^−4^ mol/L nitrate solution of each metal ion. The fluorescence intensity of the maximum emission wavelength after mixing is shown in Figure S4. After detection, it was found that RBH only had fluorescence enhancement effects on Hg^2+^, Al^3+^, Cr^3+^, Fe^3+^, and Cu^2+^. Therefore, subsequent experiments mainly focused on these five metal ions.

**FIGURE 3:**
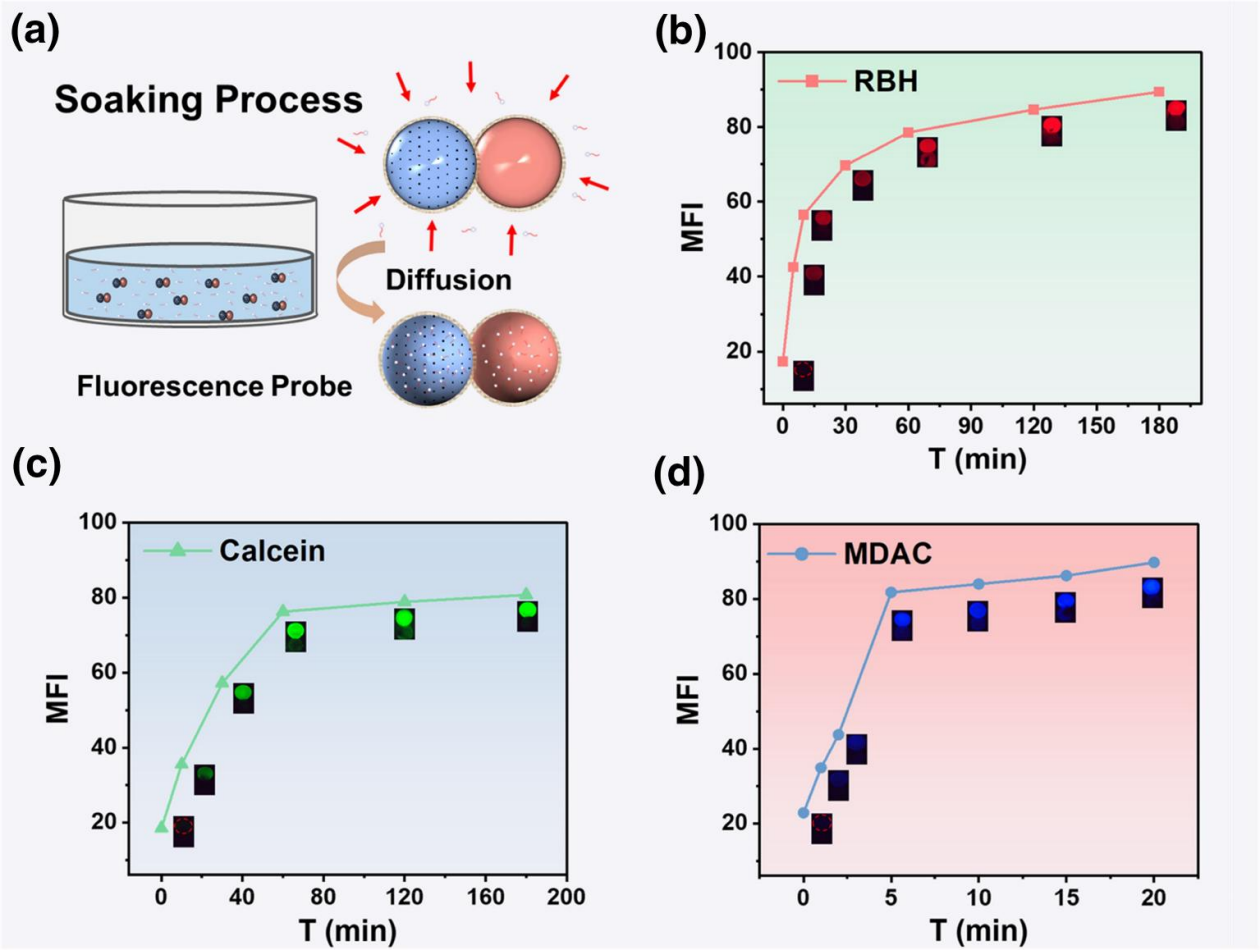
(a) Schematic diagram of the microsphere soaking process. Relationship between Mean Fluorescence Intensity (MFI) of functional microspheres and soaking time with (b) Rhodamine B Hydrazide (RBH) microspheres; (c) Calcein microspheres; (d) MDAC microspheres.

A schematic diagram of the microsphere soaking process is shown in Figure 3a. The relationship between soaking time and MFI of the functional microspheres was investigated, and the results are shown in Figures 3b to 3d. The MFI of RBH microspheres was gradually enhanced with the increase of soaking time after adding 10^−3^ mol/L Hg^2+^, and MFI did not change much when the soaking time reached 180 min. The MFI of Calcein microspheres and MDAC microspheres showed the same trend as RBH microspheres, and the MFI reached the maximum after soaking for 60 and 20 min, respectively. As time progresses, the accumulation of the fluorescent probe within the microspheres can potentially result in self‐quenching. Therefore, stringent regulation of the immersion time was employed as a preventive measure against the occurrence of this phenomenon. 180 min, 60 min, and 20 min were chosen as the soaking loading times for the above three fluorescent probes.

### 3.3 Detection of metal ions by functional microspheres

**FIGURE 4:**
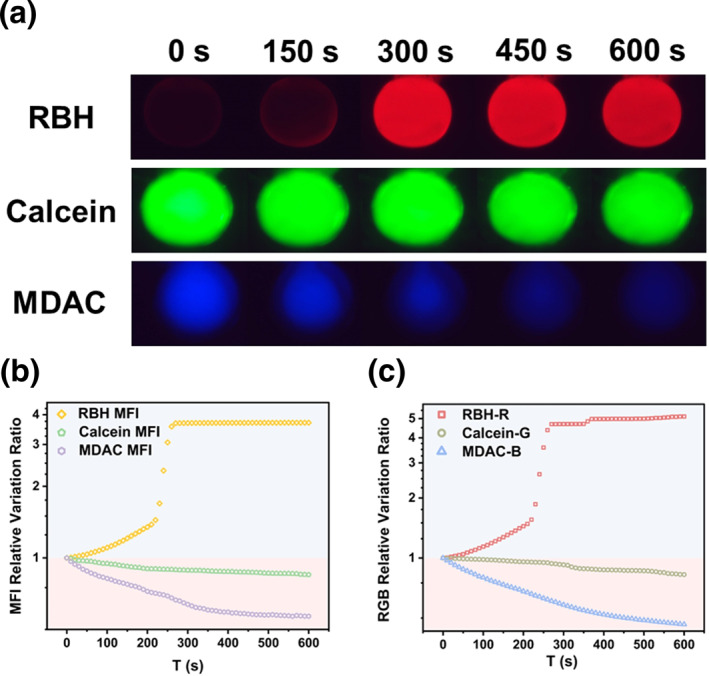
Results of 1 × 10^−3^ mol/L Hg^2+^ solution. (a) Fluorescence microscope images of three types of functional microspheres during Hg^2+^ detection over time; (b) relationship between Mean Fluorescence Intensity (MFI) of three types of functional microspheres and detection time; (c) relationship between R, G, B channel values corresponding to three types of functional microspheres and detection time.

Functional microspheres were immobilized in the microchannel and utilized for detecting various concentrations of metal ions. The outcome of detecting 1 × 10^−3^ mol/L Hg^2+^ using three functional microspheres is depicted in Figure 4a. The MFI variations of the functional microspheres were measured through Image J software, presented in Figure 4b. In addition to this, the images of functional microspheres were identified using a program written by Python, and variations of corresponding R, G, and B channel values are displayed in Figure 4c. The characteristic curve of metal ions can be obtained from the variations of the MFI and the corresponding R, G, and B channel values of the functional microspheres to identify these ions. The characteristic curves for three different concentrations of the five metal ions are shown in Figure S5‐S9.

Apart from detecting single metal ions, three functional microspheres were also employed for detecting hybrid solutions of two diverse concentrations of metal ions. Al^3+^, Cu^2+^, and Hg^2+^ were selected as the ions to be detected, and the three concentrations of 1 × 10^−3^ mol/L, 5 × 10^−4^ mol/L, and 2 × 10^−4^ mol/L were set the same as single metal ion detection. The two solutions were mixed randomly and used a fluorescence microscope for in situ observation. Because of the amount of data, we processed only a mixture of the three metal ions at a concentration of 1 × 10^−3^ mol/L (Figure S10). We have developed an ML model that enables comprehensive analysis, learning, and accurate prediction of both metal ion species and concentrations using the extensive dataset we have obtained. See Section 3.5 for a detailed discussion.

We also investigated the Method Detection Limit (MDL) of Hg^2+^ by RBH microspheres due to the good detection of Hg^2+^ by RBH microspheres. Five distinct concentrations of Hg^2+^ solutions ranging from 1 × 10^−7^ mol/L to 1 × 10^−6^ mol/L were selected and reacted with RBH microspheres to fit a line about the variation of the MFI of RBH microspheres and Hg^2+^ concentration. Each group of experiments was conducted three times in parallel. Then, the MFI of Hg^2+^ solution having a concentration of 2 × 10^−7^ mol/L was detected 10 times in parallel. The corresponding concentration was obtained by substituting the fitting line, and the standard deviation and t‐distribution values of the samples were calculated to acquire the MDL. The MDL of RBH functional microspheres for Hg^2+^ was calculated to be 2.4 × 10^−7^ mol/L or 0.0482 ppm, lower than the national emission standard of 0.05 ppm for Hg^2+^ in industrial wastewater. The experimental results are presented in the Supporting Information (Figure S11 and Table S1). Thus, RBH microspheres can be utilized for the fluorescence detection of Hg^2+^ in industrial wastewater, and the microspheres can be recycled by magnetic control, which has vast application potential.

### 3.4 Processing experimental data using PCA

A large amount of data containing multiple variables is obtained using fluorescence microscopy in situ observation and image processing program recognition, and the variables are somewhat interrelated. If each variable is analyzed individually, the analysis is often isolated and cannot fully utilize the information contained in the data. Blindly reducing variables will result in the loss of useful information and lead to erroneous conclusions. Therefore, a reasonable method is needed to reduce variables while minimizing the loss of original data information to analyze the collected data comprehensively. Since there is a certain correlation between variables, it is possible to transform closely related variables into as few new variables as possible, making these new variables uncorrelated with each other. Thus, fewer PCs can represent various types of information present in each variable. Therefore, data are processed through the dimensionality reduction, that is, PCA. After preliminary screening, 13 variables were selected, including time, the three fluorescent probes' variation of MFI, and variations in R, G, and B channel values of each probe's corresponding image. The data from 61 groups of 1 × 10^−3^ mol/L Hg^2+^ solution detections were processed. PCA requires some correlation between multidimensional indicators, so the data is first subjected to correlation analysis. The correlation heatmap obtained shows that most correlation coefficients between variables are more significant than 0.3 (Figure S12). Therefore, the data is further processed to obtain a biplot. After data reduction, the R^2^ explained by the two PCs is over 95% for the entire data set.

**FIGURE 5:**
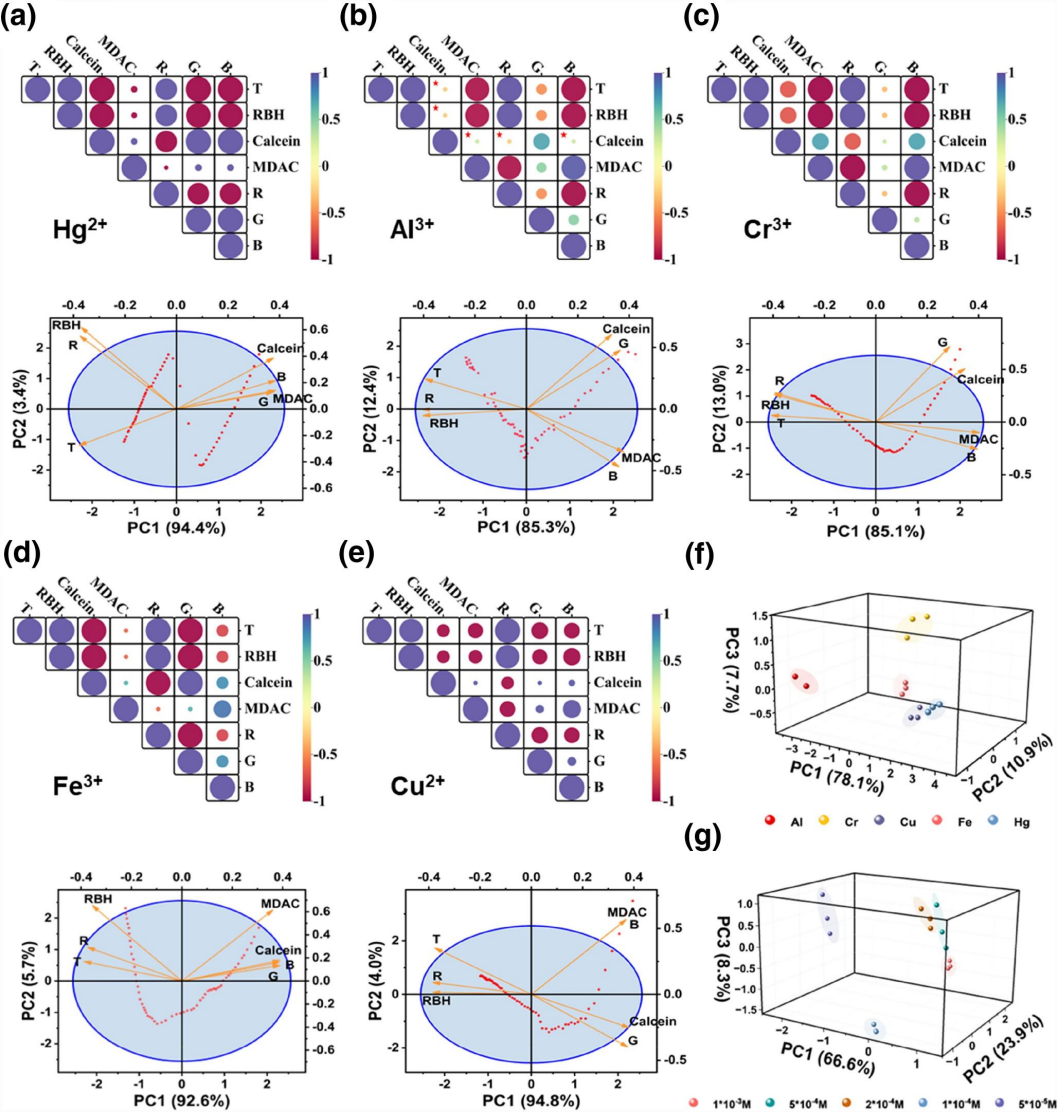
Correlation heat map and Principal Component Analysis (PCA) of five metal ions. (a) Hg^2+^; (b) Al^3+^; (c) Cr^3+^; (d) Fe^3+^; (e) Cu^2+^; (f) PCA of five different metal ions; (g) PCA of different concentrations of Hg^2+^ (Cells with correlation coefficients less than 0.3 are marked with a red pentagram).

Through the PCA biplot of Hg^2+^, it is found that multiple groups of data overlap. Data processing found that the variables that changed the most during the detection of metal ions were the R channel value of RBH, the G channel value of Calcein, and the B channel value of MDAC. Therefore, after further variable selection, 7 variables were obtained, including time T, the three fluorescent probes' variation of MFI, and the RBH‐R, Calcein‐G, and MDAC‐B channel values. After data simplification, PCA has performed again on the five metal ions at a 1 × 10^−3^ mol/L concentration. The data is first subjected to correlation analysis, and Kendall's tau‐c rank correlation analysis is used to process the correlation between two variables, as shown in Figure 5a‐e (RBH represents the variation of MFI, and R represents the R‐channel value variation of RBH, Calcein, and MDAC are similar to RBH). The size and color of the circle represent the correlation relationship between the two variables. The obtained correlation heatmap shows that the correlation coefficients between most variables of Hg^2+^ and Cu^2+^ are much greater than 0.3, and the correlation coefficients between variables of Cr^3+^ and Fe^3+^ are greater than 0.3, while the correlation coefficients between most variables of Al^3+^ are also greater than 0.3, which meets the requirements of PCA.

Additionally, KMO and Bartlett's sphericity tests were performed on the data. The KMO test checks the correlation between variables, and Bartlett's sphericity test determines whether the variables are independent. The results also proved that the collected data is suitable for PCA. After further processing the data, the biplot was obtained with two extracted PCs explaining over 95% of the R^2^ for the entire data set. Taking Hg^2+^ as an example (as shown in Figure 5a), the projection of loading on the x and y axes represents the correlation between the variable and the extracted PC (positive or negative correlation and relative size). For example, the MFI of MDAC is positively correlated with PC1, and the MFI of RBH contributes significantly to PC2. The smaller the angle between the loadings, the stronger the correlation between the two variables. By studying the PCA biplots of the five metal ions, it is found that variations in the MFI of the fluorescent probe and its corresponding R, G, and B channel values are closely related. The score plot of Hg^2+^ is similar to a sine wave function, while the score plots of the other four metal ions are similar to cosine wave functions. The score plots can easily classify Hg^2+^ and other metal ions.

In addition, the distance between points on the score plot reflects the difference in sample data. In the PCA score plot, if several sample points are clustered together, the similarity between these samples is very high. Conversely, if several sample points are widely scattered, it indicates that the similarity between these samples is relatively low. Therefore, to further cluster analysis of the metal ions, we measured the six variables of the five metal ions three times in parallel at 600 s. The results of the PCA are shown in Figure 5f. The scattered points corresponding to different metal ions show clustering with each other within the group, which indicates that the repeatability within the group is relatively good and the sample data are very similar. At the same time, there was good discrimination between the groups. Therefore, the method of PCA can be used for the clustering analysis of metal ions. We have also performed PCA on solutions of different concentrations of the same metal ions, and the results are shown in Figure 5g. PCA can classify the different concentration solutions of Hg^2+^.

### 3.5 ML model to predict metal ion species and concentrations

After obtaining a large amount of data, we established an ML model. Firstly, the results of single metal ion detection were imported. 61 Sets of data were detected each time, corresponding to three concentrations of five metal ions, for a total of 915 sets of data as shown in Supporting Information Sheet 1. 70% of the data were used to establish the ML model, and 30% of the data was used to test the learning results of the model. All data were randomly selected by code without selection bias. The model had seven descriptors, including time, the MFI of three fluorescent probes, the R channel value of RBH, the G channel value of Calcein, and the B channel value of MDAC. The output results were the species and concentration of the metal ion. Different metal ions were distinguished by their atomic number, and the concentration was directly output with a unit of 10^−4^ mol/L. We used the predicted value as the independent variable and the ground truth as the dependent variable, fitted the function curve and obtained the R^2^ and root mean square error (RMSE) of the fitted curve. The larger the R^2^ and the smaller the RMSE, the closer the curve was to the “y = x” plot, which indicated that the predicted value was closer to the ground truth and the better the prediction performance of the model.

**FIGURE 6:**
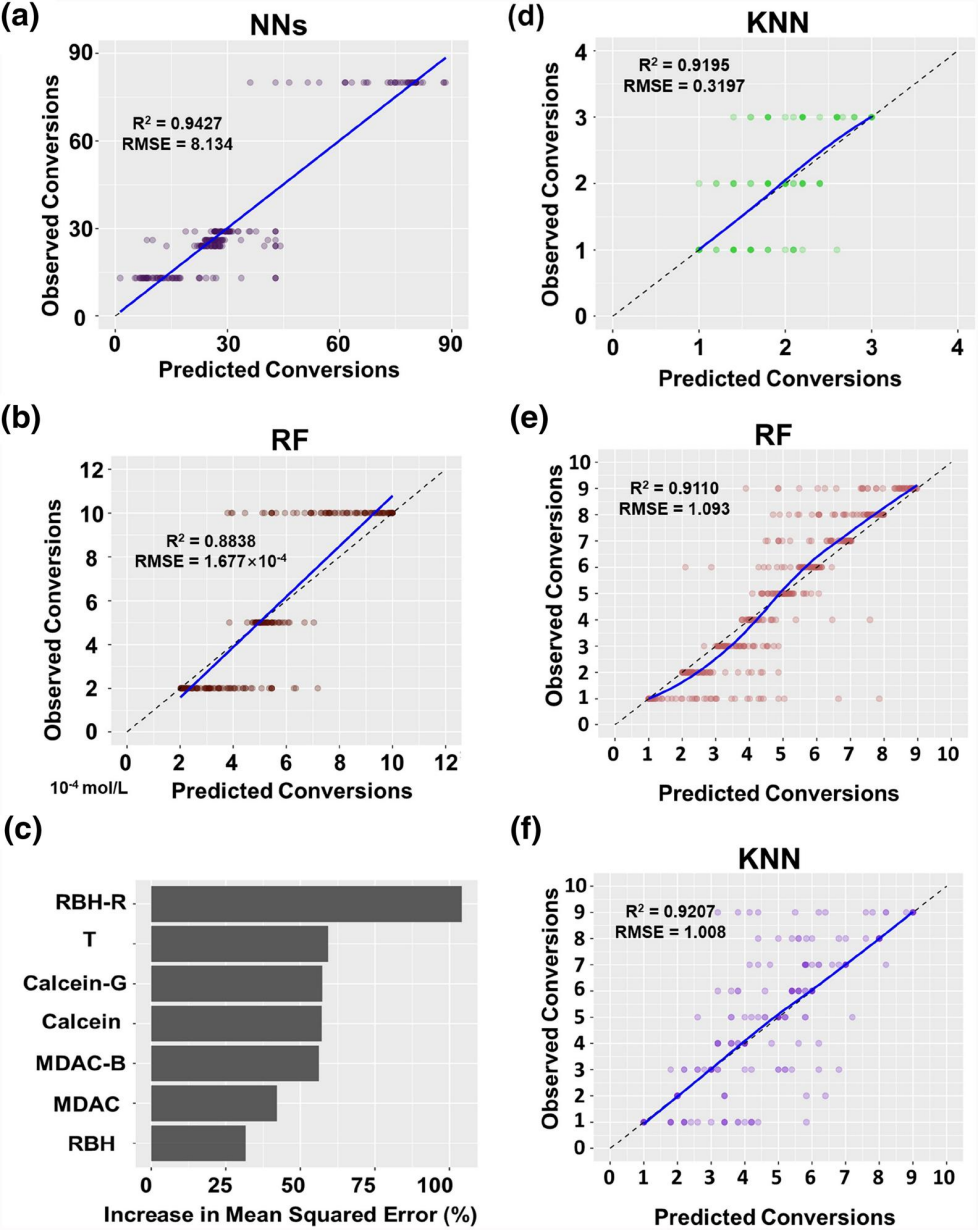
Optimal results of Machine Learning (ML) models predicting the species and concentration of metal ions. Prediction results of (a) species and (b) concentration of single metal ion; (c) importance analysis of the prediction of the concentration of a single metal ions by Random Forest (RF) model; prediction results of (d) species of hybrid metal ions; prediction results of hybrid metal ions' concentrations (e) before and (f) after differentiation of ion species.

To establish ML models, six algorithms, including RF, LR, KNN, SVM, Bys, and NN were used. Firstly, for studying metal ion species, the NN algorithm established the best model for predicting metal ion species with R^2^ of 0.94 and the smallest RMSE. Its function plot is shown in Figure 6a, and the color transparency of data points represents the degree of data overlap. The data points were concentrated on the “y = x” line, indicating a better‐fitting effect. Then, for predicting the metal ion concentration, we found that the KNN and RF algorithms established better models for predicting concentrations. The fitting line of the RF model is shown in Figure 6b, which revealed higher prediction results for low concentration and lower prediction results for high concentration, the units are 10^−4^ mol/L. The model needs more data for calibration. Figure 6c shows the percentage of importance of each feature descriptor in the RF model training after the model training, which is the percentage of RMSE increases when the variable's value is randomly shuffled, and the model is retrained. The model considers the R channel value of RBH to be the most important factor in model training. If the RBH‐R changes, the RMSE increases by more than 100%. The prediction results of ML models for the species and concentrations of single metal ions using different algorithms are shown in Table 1.

**TABLE 1:** Prediction of single metal ions by the Machine Learning (ML) model.

Algorithm	M‐R^2^	M‐RMSE	C‐R^2^	C‐RMSE
RF	0.8178	14.06	0.8838	1.677 × 10^−4^
LR	0.6433	18.73	0.5605	2.879 × 10^−4^
KNN	0.8453	13.15	0.8788	1.657 × 10^−4^
SVM	0.5840	20.07	0.5594	2.931 × 10^−4^
Bys	0.6432	18.73	0.5604	2.879 × 10^−4^
NN	0.9427	8.134	0.0931	3.472 × 10^−4^

Since there are not only single metal ions in metal ion detection, we also tested hybrid metal ion solutions. Three metal ions were selected with relatively high physiological toxicity, Al^3+^, Cu^2+^, and Hg^2+^, and set three concentrations for each metal ion to mix them in pairs, resulting in 27 mixing methods. When establishing the model, for convenience, the metal ion mixing types were replaced with numbers, where 1 represented Al^3+^ and Cu^2+^, 2 represented Al^3+^ and Hg^2+^, and 3 represented Cu^2+^ and Hg^2+^. As for the prediction of metal ion concentration, the situation was more complicated. Numbers were also used to represent the model output results, and information on the corresponding relationship between the metal ion concentration and the prediction results of the model can be found in Supporting Information Table S2. A total of 27 experiments were performed to detect hybrid metal ion solutions comprising a total of 1647 data sets which are presented in Supporting Information Sheet 2. After importing the data for model building, the prediction results of the ML models built by various algorithms for metal ion species and concentrations are shown in Table 2.

**TABLE 2:** Prediction of hybrid metal ions by the Machine Learning (ML) model.

Algorithm	M‐R^2^	M‐RMSE	C‐R^2^‐1	C‐RMSE‐1	C‐R^2^‐2	C‐RMSE‐2
RF	0.8870	0.3823	0.9110	1.093	0.9178	1.052
LR	0.4443	0.7305	0.5131	2.221	0.5130	2.221
KNN	0.9195	0.3197	0.8987	1.134	0.9207	1.008
SVM	0.4025	0.7729	0.5000	2.264	0.4960	2.283
Bys	0.4443	0.7305	0.5131	2.221	0.5130	2.221
NN	0.9100	0.3365	0.8584	1.330	0.9017	1.119

Firstly, the species of metal ions were predicted. The KNN and NN models have good prediction performances, with R^2^ exceeding 0.9 and RMSE less than 0.4, so the prediction accuracy is high. The fitting curve of the KNN model is shown in Figure 6d, which can satisfy the requirement for predicting metal ion species. Next, the concentration of metal ions was predicted. The RF model had the best prediction performance if the metal ions were not distinguished (Figure 6e). If the metal ions were distinguished, that is, if the metal ion species were added as descriptors, the prediction performance of the other models did not change much except for the KNN and NN models. The KNN model had the best prediction performance after distinguishing the metal ion with an R^2^ of 0.92 and the smallest RMSE (Figure 6f). The prediction performance of the NN model was also improved. The importance analysis comparison chart by the RF model before and after distinguishing the metal ions can be found in Figure S13. Adding the metal ion species as descriptors did not significantly impact the RMSE of the RF model. Therefore, it is recommended to distinguish the metal ions before importing the data into practical applications and establish a KNN model for predicting the metal ions. The prediction results of the optimal model for each case are shown in the Supporting Information Sheet 3.

After obtaining the optimal model for each case, the evaluation metrics of the optimal model concerning the prediction results except R^2^ and RMSE were also calculated. The Mean Absolute Error (MAE) and Median Absolute Error (MedAE) of the prediction results were calculated, that is, the mean and median of the prediction errors, and the results are shown in Table 3. Extreme values observably influence the MAE. The MedAE of all five models is smaller than the MAE, which indicates that there are some data errors in the model predictions larger. The MedAE of the prediction models for hybrid metal ion species and concentrations after differentiation of metal ion species are both 0, which indicates that the prediction effect of these two models is better. Mean Absolute Error and RMSE were used together, and the degree of dispersion of the sampling error could be seen. For example, when RMSE is much larger than MAE, it can be known that the errors of different samples differ significantly. The results show that the optimal model selected RMSE is slightly larger than MAE, and the dispersion of the sampling error is small. Overall, the prediction methods used in this study are effective for predicting metal ion species and concentrations. The KNN and NN models have high accuracy in predicting metal ion species, while the RF model performs well in predicting metal ion concentrations. The addition of metal ion species as descriptors improved the performance of the KNN and NN models. The evaluation metrics, such as R^2^, RMSE, MAE, and MedAE, demonstrate that the optimal models have good prediction accuracy with small errors and low dispersion of sampling error. Therefore, these models can be useful in practical applications for predicting metal ion species and concentrations.

**TABLE 3:** Evaluation indexes of the optimal model prediction result in each case.

Algorithm	MAE	MedAE
Single Metal‐M‐NN	4.526	1.857
Single Metal‐C‐RF	1.020 × 10^−4^	4.460 × 10^−5^
Hybrid Metal‐M‐KNN	0.1312	0.0000
Hybrid Metal‐C‐RF	0.6140	0.2404
Hybrid Metal‐C‐KNN	0.4013	0.0000

## 4 CONCLUSION

In this study, we developed a multifunctional microsphere that combined magnetic control and fluorescence detection using the droplet templating method. Different metal ions can be detected by controlling the fluorescent probe type. Three fluorescent probes, RBH, Calcein, and MDAC were selected and found that RBH functional microspheres' MDL for Hg^2+^ is 0.0482 ppm, lower than the national discharge standard for industrial wastewater. Through fluorescence microscopy and image processing program to obtain large amounts of data, which can be used to obtain characteristic curves for the five metal ions of Hg^2+^, Al^3+^, Cr^3+^, Fe^3+^, and Cu^2+^, and the “fingerprint spectrums” for the five metal ions were also provided by PCA. Machine Learning methods were used to analyze the data further and found that RF, KNN, and NN models had good predictive effects on the concentration and species of metal ions. With the help of ML algorithms and image analysis techniques, it is possible to perform a preliminary analysis of metal ion species and concentrations without the use of large instruments such as atomic spectroscopy. Overall, the study suggests that using microfluidic technology, functional polymer microspheres, and ML algorithms holds great promise for developing high‐performance metal ion detection and prediction systems.

## CONFLICT OF INTEREST STATEMENT

The authors declare no conflicts of interest.

## ETHICS STATEMENT

No animal or human experiments were involved in this study.

## Supporting information

Supporting Information S1

Supporting Information S2

## Data Availability

The data that support the findings of this study are available from the corresponding author upon reasonable request.
