# Supplementary material for: Magnetic‐optical dual functional Janus particles for the detection of metal ions assisted by machine learning
Source: Smart Mol. 2023 Sep 19;1(2):e20230006. doi: 10.1002/smo.20230006 (PMC12118306; doi:10.1002/smo.20230006)
Supplement: Supplementary file 1 — Supporting Information S1 [file SMO2-1-e20230006-s002.pdf]

Supporting Information

Magnetic-Optical Dual Functional Janus Particles for the Detection of Metal Ions Assisted by Machine Learning

*Jianhang Liu, Yingdi Lv, Xinghai Li, Shi Feng, Wenbo Yang, Yumeng Zhou, Shengyang Tao**

J. Liu, X. Li, S. Feng, W. Yang, S. Tao

State Key Laboratory of Fine Chemicals, Frontier Science Center for Smart Materials, School of Chemistry, Dalian University of Technology, Dalian 116024, China

Email: taosy@dlut.edu.cn

Y. Zhou

Instrumental Analysis Center, Dalian University of Technology, Dalian 116024, China

Y. Lv

School of Chemistry and Chemical Engineering, Northwestern Polytechnical University, Xi'an 710072, China

Xi'an Modern Chemistry Research Institute, Xi'an 710065, China

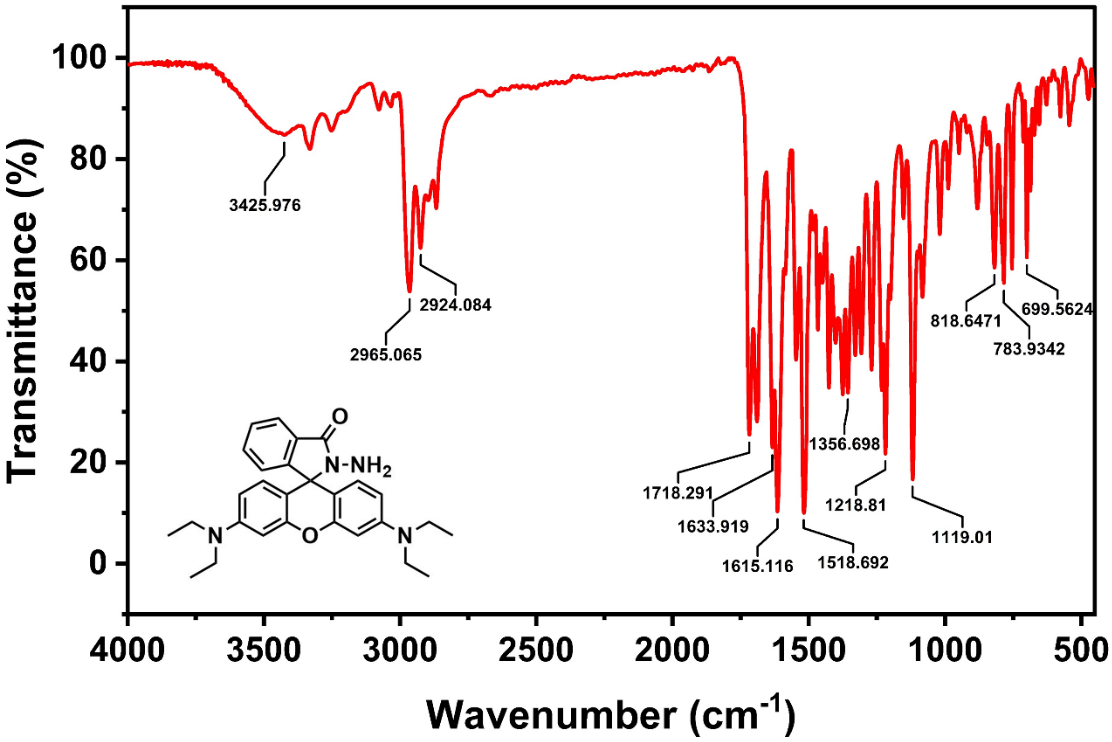

Figure S1. Infrared spectra analysis of RBH

The peak at 3425.98 cm^{-1} is the stretching vibration absorption peak of −NH. The peeks at 2965.07 cm^{-1}, 2924.08 cm^{-1} can be attributed to the stretching vibration of C−H attached to top of the parent ring with the nitrogen atom. The peak at 1718.29 cm^{-1} is the absorption peak of carbonyl C-O on the amide. The peeks at 1633.92 cm^{-1}, 1615.12 cm^{-1}, 1518.69 cm^{-1} are the characteristic absorption peaks of C=C in benzene. The peak at 1356.70 cm^{-1} is the vibrational absorption peak of the C-H. The peak at 1218.81 cm^{-1} is attributed to the tertiary amine substituent. The peak at 1119.01 cm^{-1} are attributed to −C−O−C−. The peaks at 818.64 cm^{-1}, 783.93 cm^{-1}, 699.56 cm^{-1} are the absorption peaks of the C−H deformation vibration of substituted benzene. The above data further confirmed the successful synthesis of Rhodamine B Hydrazide

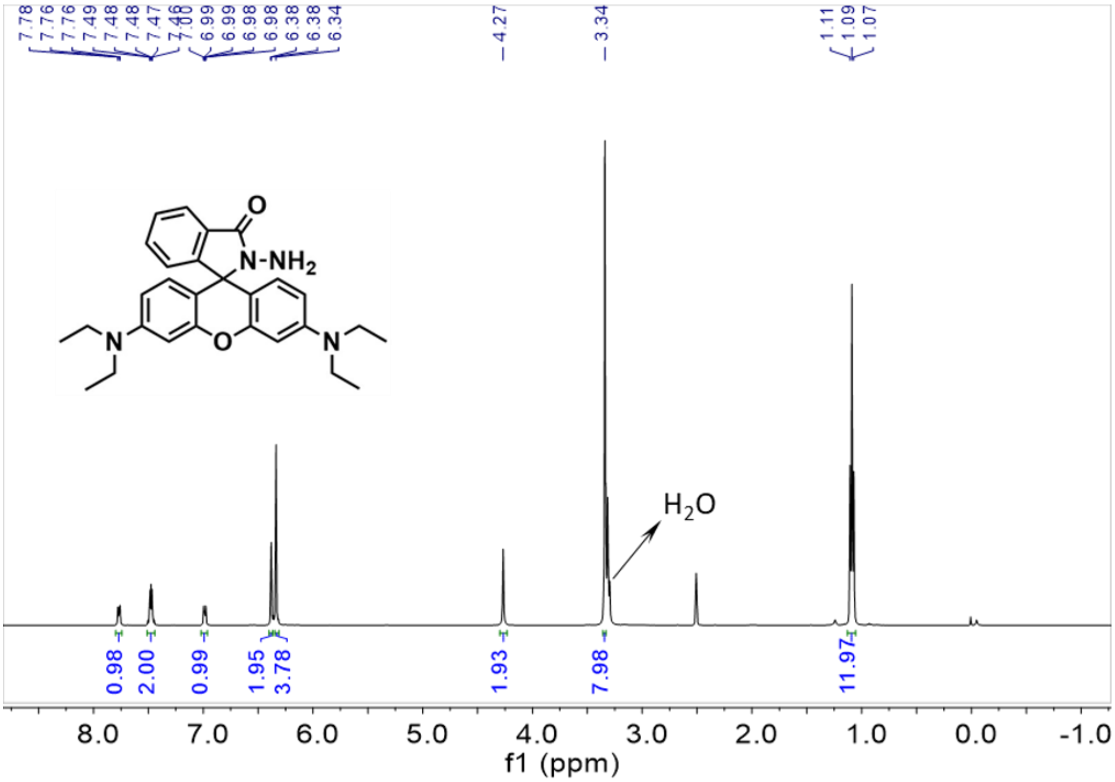

Figure S2. Nuclear magnetic resonance analysis of RBH

^{1}H NMR (400 MHz, DMSO-d_6) δ 7.81 - 7.72 (m, 1H), 7.51 - 7.44 (m, 2H), 7.02 - 6.94 (m, 1H), 6.38 (d, J = 1.7 Hz, 2H), 6.34 (m, 4H), 4.27 (s, 2H), 3.34 (m, 8H), 1.09 (t, J = 6.9 Hz, 12H).

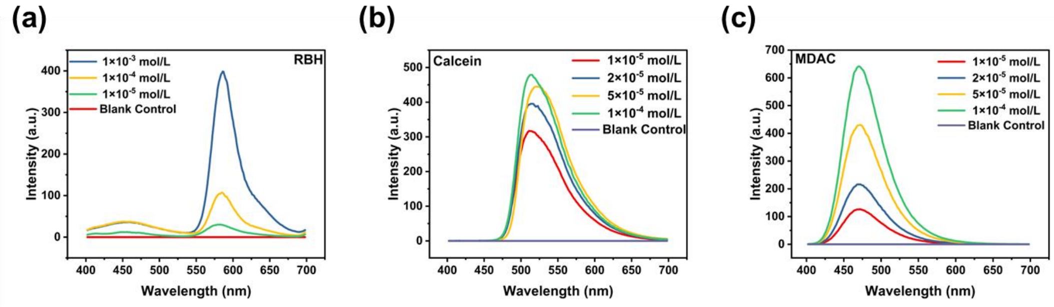

Figure S3. Optimal concentration of fluorescent probes. (a) RBH; (b) Calcein; (c) MDAC.

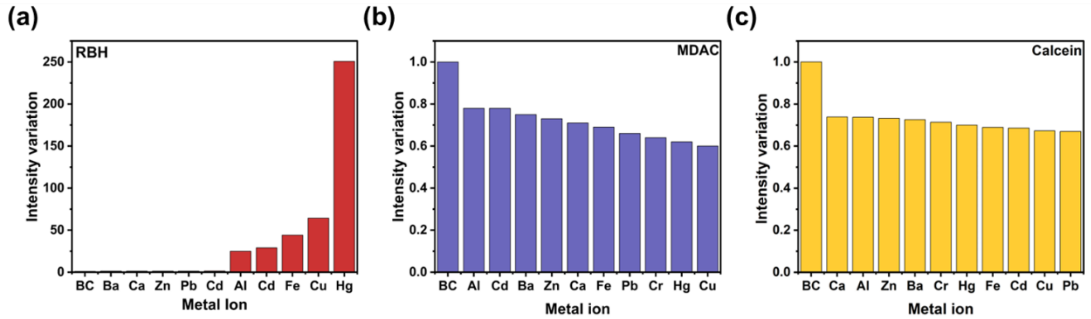

Figure S4. Screening of metal ions. (a) RBH; (b) Calcein; (c) MDAC.

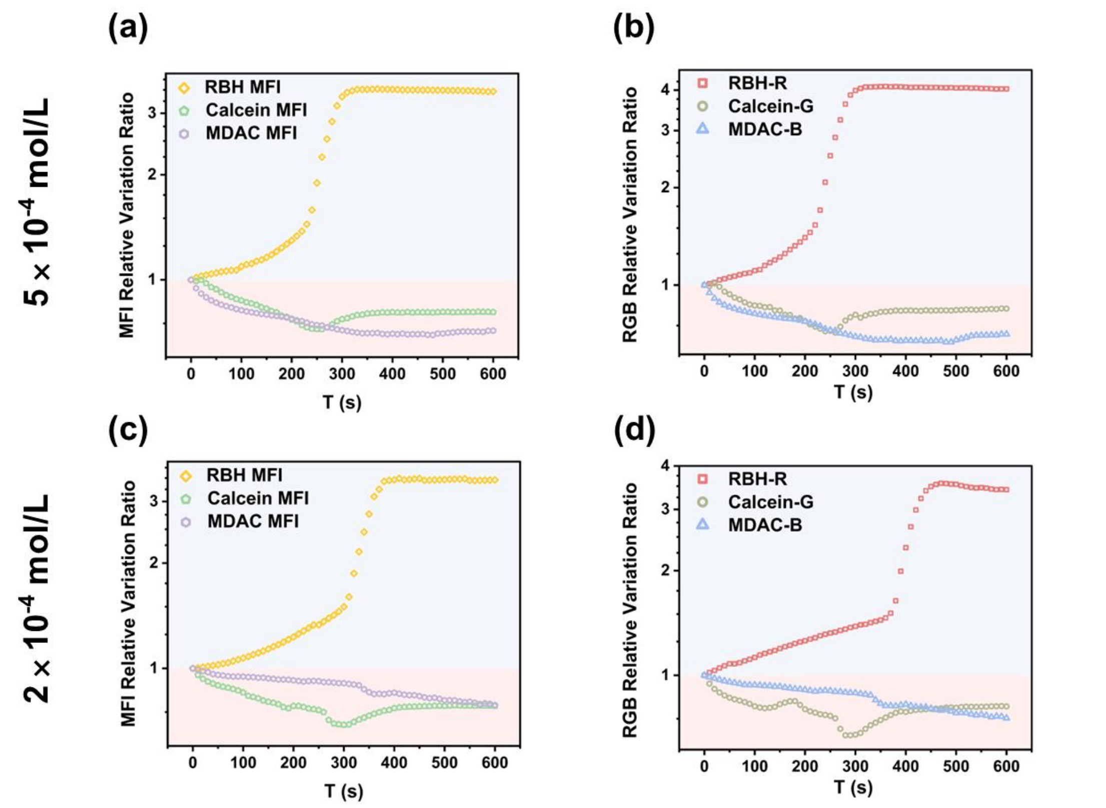

Figure S5. Characteristic curves of detecting Hg^{2+} using functional microspheres. (a), (c) show the varation of MFI at different concentration; (b), (d) show the variation of R, G, and B channel values at different concentration.

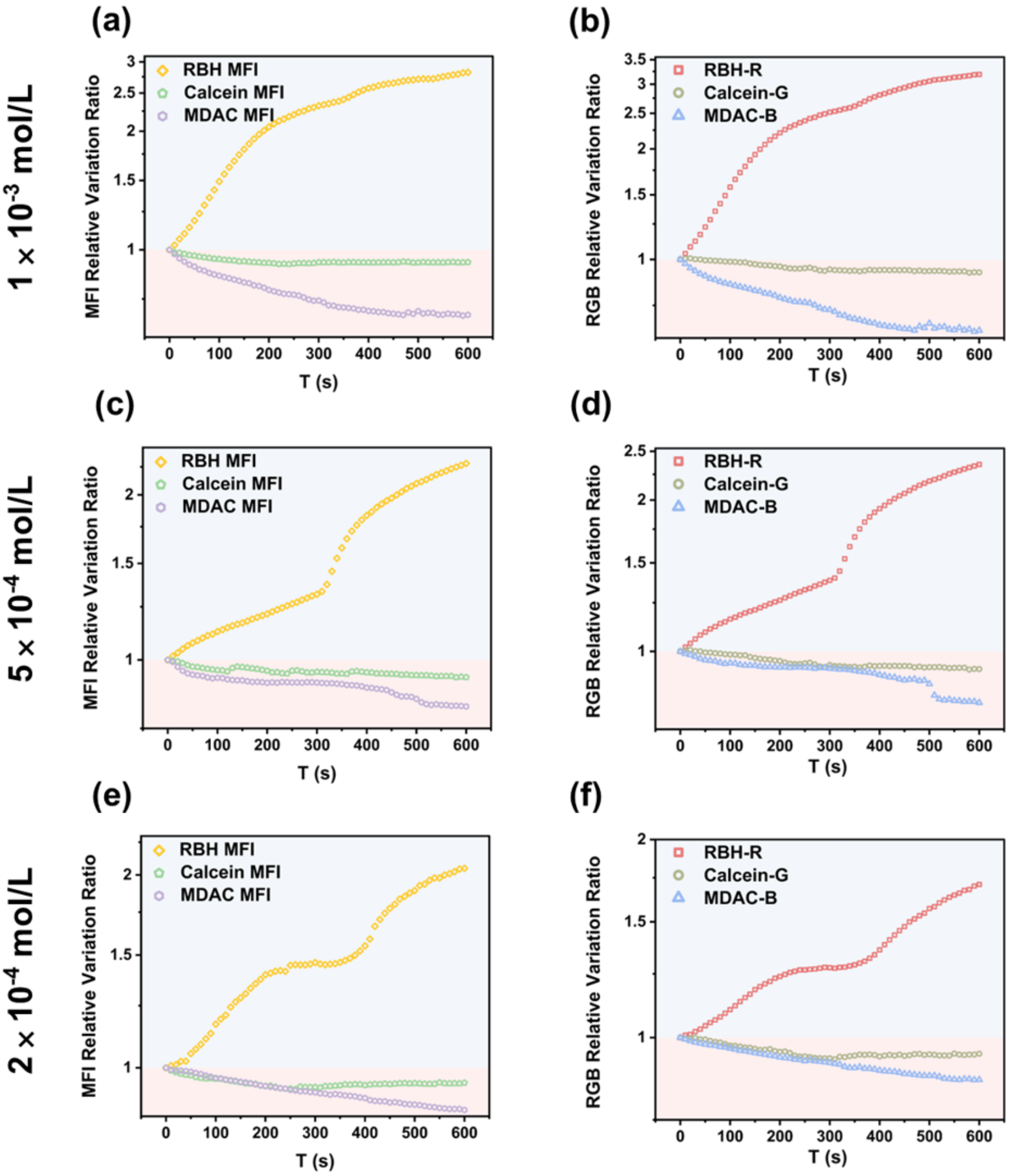

Figure S6. Characteristic curves of detecting Al^{3+} using functional microspheres. (a), (c), and (e) show the varation of MFI at different concentration; (b), (d), and (f) show the variation of R, G, and B channel values at different concentration.

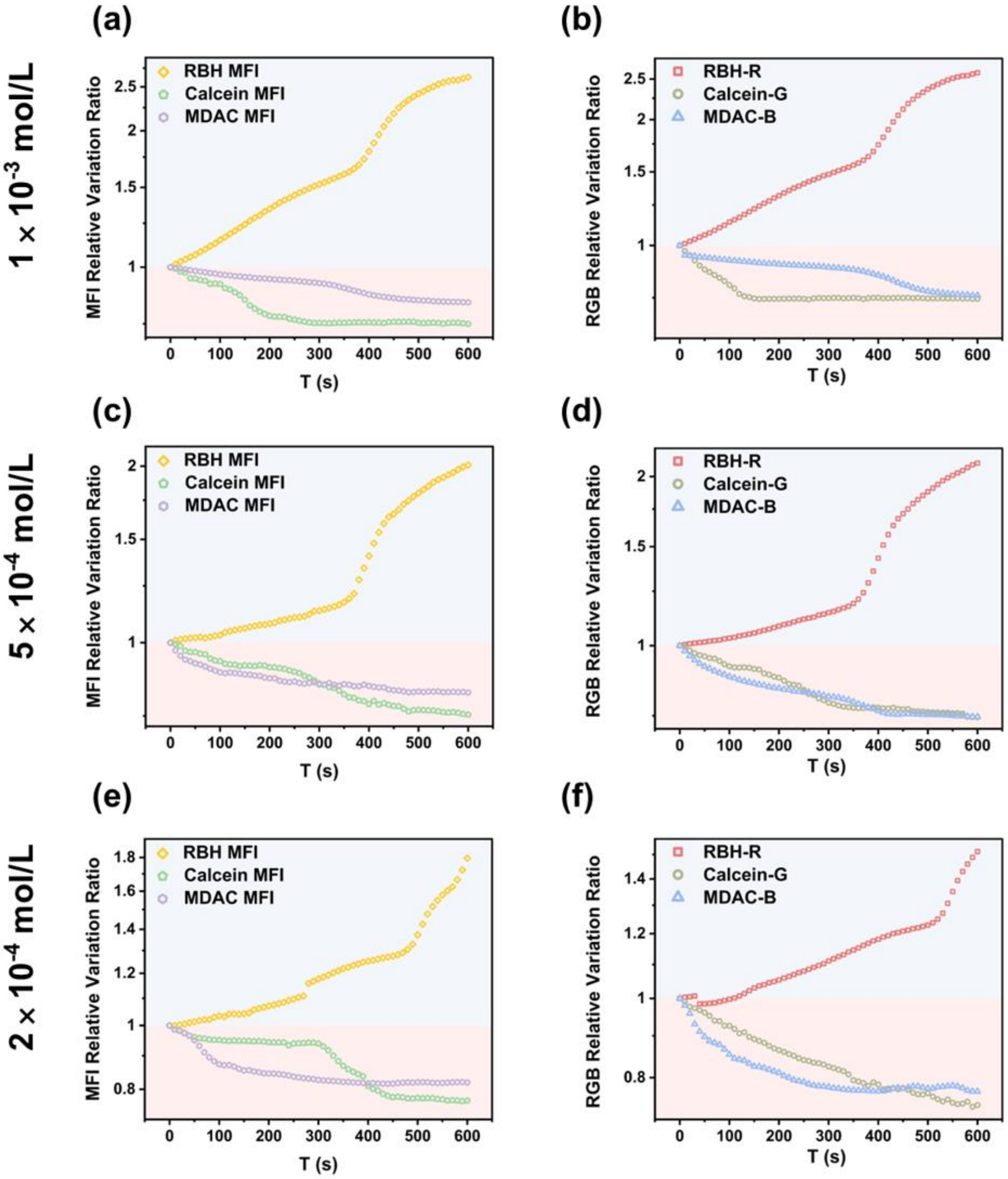

Figure S7. Characteristic curves of detecting Cr^{3+} using functional microspheres. (a), (c), and (e) show the varation of MFI at different concentration; (b), (d), and (f) show the variation of R, G, and B channel values at different concentration.

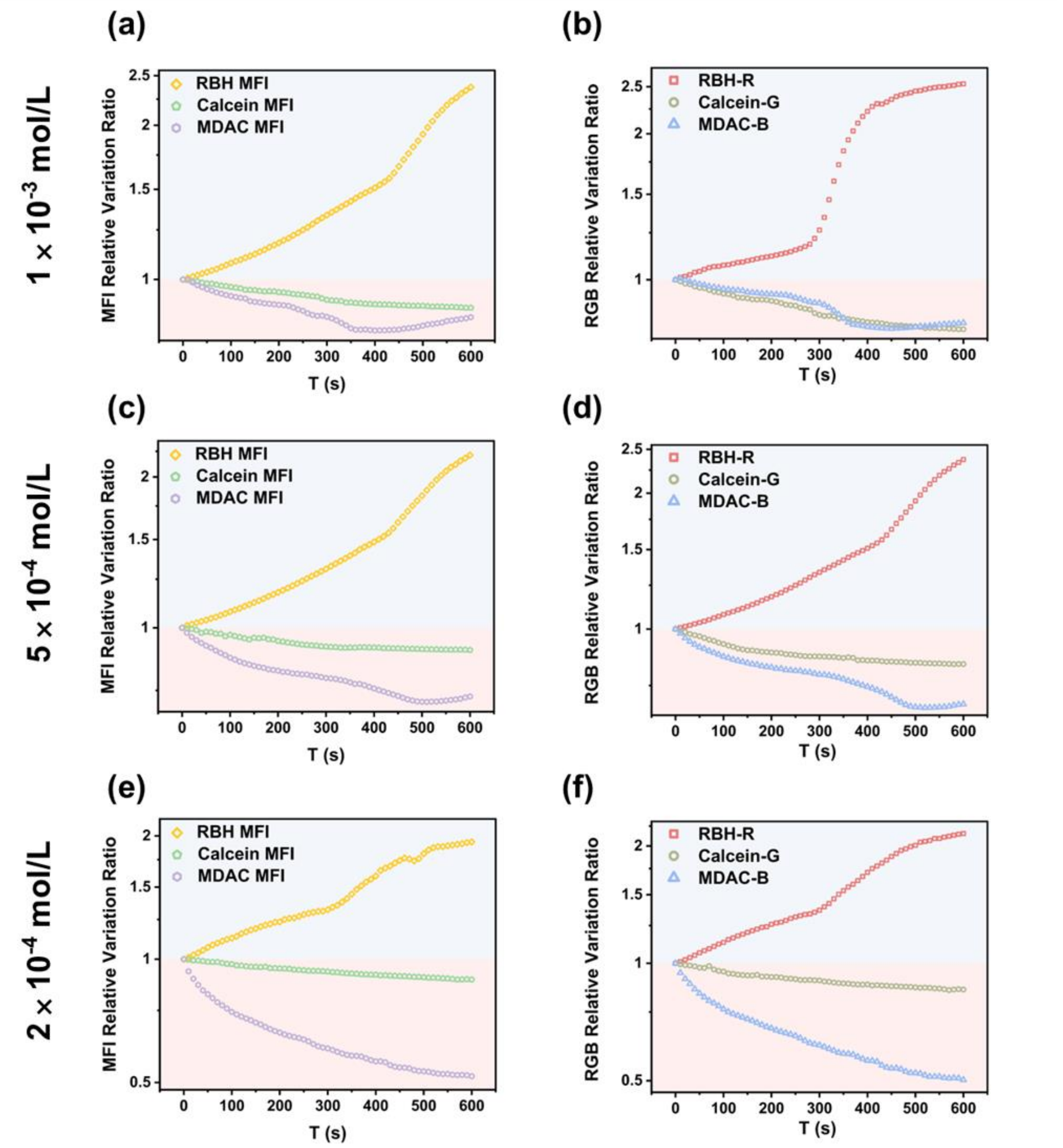

Figure S8. Characteristic curves of detecting Fe^{3+} using functional microspheres. (a), (c), and (e) show the varation of MFI at different concentration; (b), (d), and (f) show the variation of R, G, and B channel values at different concentration.

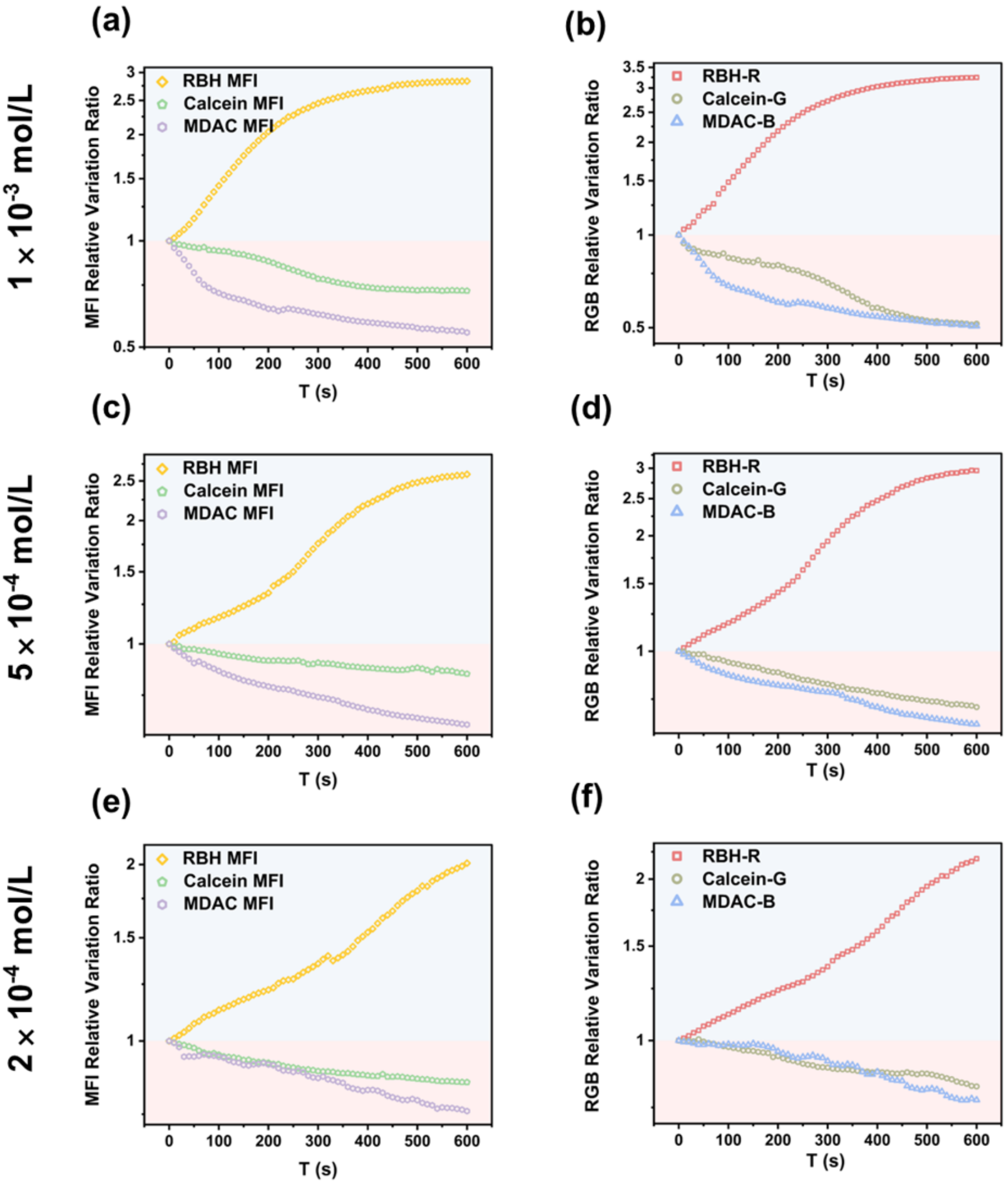

Figure S9. Characteristic curves of detecting Cu^{2+} using functional microspheres. (a), (c), and (e) show the varation of MFI at different concentration; (b), (d), and (f) show the variation of R, G, and B channel values at different concentration.

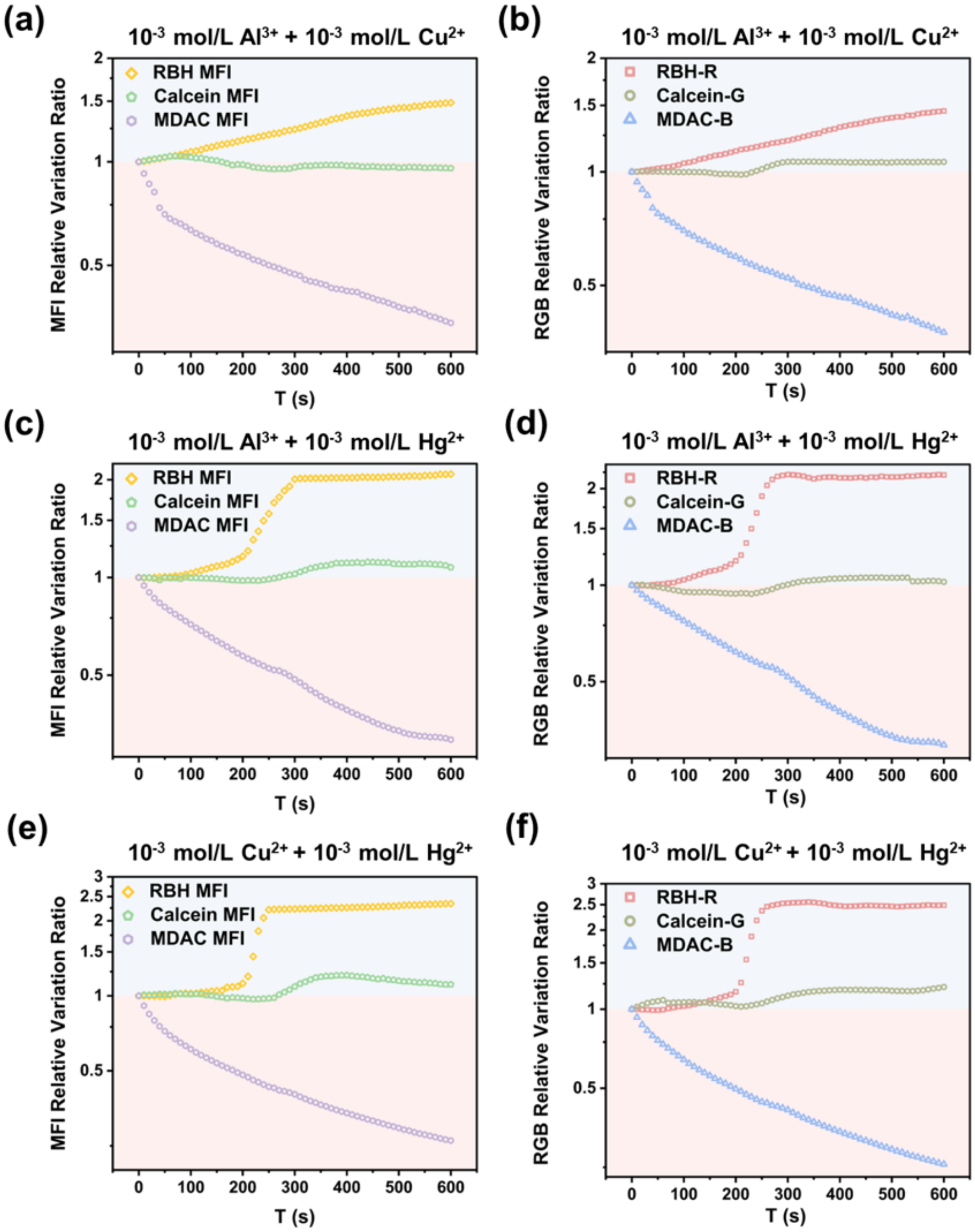

Figure S10. Characteristic curves of detecting hybrid metal ions using functional microspheres. (a), (c), and (e) show the varation of MFI; (b), (d), and (f) show the variation of R, G, and B channel values.

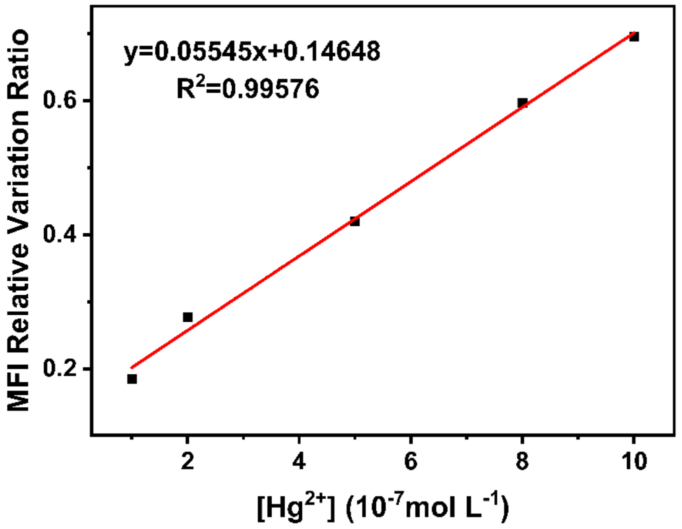

Figure S11. Fitted straight line for detection of mercury ions by RBH microspheres.

Table S1. Determination of MDL of RBH microspheres for Hg^{2+}.

MFI	1	2	3	4	5	6	7	8	9	10
Blank	13.71	18.29	15.53	15.05	14.28	17.82	16.47	15.94	16.27	17.71
Add Hg^{2+}	16.94	22.18	21.91	20.70	18.72	22.95	19.86	18.95	20.08	22.35
Variation	0.236	0.213	0.411	0.376	0.311	0.288	0.206	0.189	0.234	0.262
c (10^{-7} mol/L)	1.608	1.196	4.778	4.137	2.964	2.549	1.080	0.762	1.587	2.082

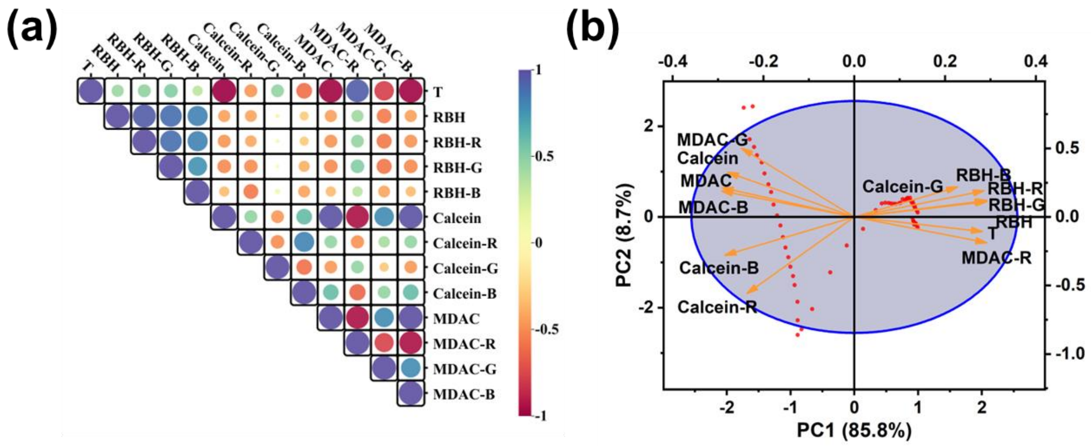

Figure S12. (a) Multivariate correlation heatmap and (b) PCA biplot.

Table S2. Correspondence between output results and concentration for prediction of hybrid metal ions.

	1	2	3	4	5	6	7	8	9
A [mol/L] [a)]	1×10^{-3}	1×10^{-3}	1×10^{-3}	5×10^{-4}	5×10^{-4}	5×10^{-4}	2×10^{-4}	2×10^{-4}	2×10^{-4}
B [mol/L] [b)]	1×10^{-3}	5×10^{-4}	2×10^{-4}	1×10^{-3}	5×10^{-4}	2×10^{-4}	1×10^{-3}	5×10^{-4}	2×10^{-4}

[a)] A is the metal ion with small atomic number; [b)] B is the metal ion with large atomic number.

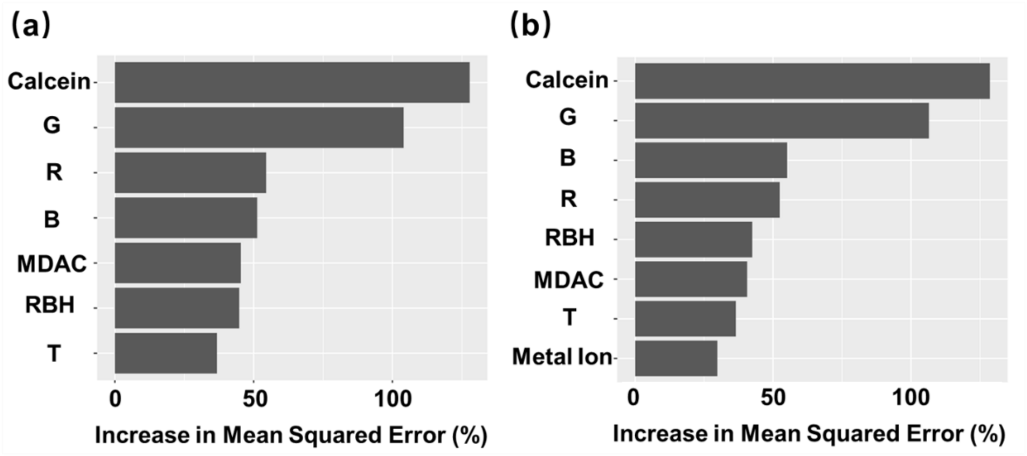

Figure S13. Comparison of importance analysis before and after differentiation of metal ions by RF model.
