# Supplementary material for: Magnetic‐optical dual functional Janus particles for the detection of metal ions assisted by machine learning
Source: Smart Mol. 2023 Sep 19;1(2):e20230006. doi: 10.1002/smo.20230006 (PMC12118306; doi:10.1002/smo.20230006)
Supplement: Supplementary file 2 — Supporting Information S2 [file SMO2-1-e20230006-s001.pdf]

Sheet 1

Hg^{2+}	1*10^{-3} mol/L					
t (s)	RBH-MFI	RBH-R	Calcein-MFI	Calcein-G	MDAC-MFI	MDAC-B
0.0000	1.0000	1.0000	1.0000	1.0000	1.0000	1.0000
10.0000	1.0033	1.0048	0.9940	0.9981	0.9664	0.9724
20.0000	1.0113	1.0145	0.9834	0.9961	0.9397	0.9465
30.0000	1.0192	1.0242	0.9757	0.9940	0.9208	0.9212
40.0000	1.0266	1.0338	0.9735	0.9925	0.8966	0.9001
50.0000	1.0352	1.0454	0.9705	0.9902	0.8807	0.8788
60.0000	1.0483	1.0647	0.9675	0.9887	0.8641	0.8611
70.0000	1.0612	1.0821	0.9609	0.9872	0.8523	0.8446
80.0000	1.0740	1.0986	0.9544	0.9863	0.8375	0.8270
90.0000	1.0884	1.1198	0.9487	0.9846	0.8294	0.8085
100.0000	1.1048	1.1401	0.9479	0.9831	0.8196	0.7988
110.0000	1.1215	1.1604	0.9423	0.9806	0.8105	0.7843
120.0000	1.1401	1.1874	0.9400	0.9791	0.7992	0.7706
130.0000	1.1622	1.2145	0.9323	0.9767	0.7910	0.7578
140.0000	1.1838	1.2416	0.9263	0.9729	0.7837	0.7473
150.0000	1.2095	1.2725	0.9227	0.9718	0.7750	0.7356
160.0000	1.2367	1.3053	0.9171	0.9671	0.7657	0.7240
170.0000	1.2636	1.3372	0.9133	0.9652	0.7593	0.7137
180.0000	1.2914	1.3710	0.9108	0.9617	0.7492	0.7038
190.0000	1.3230	1.4106	0.9044	0.9590	0.7369	0.6944
200.0000	1.3491	1.4435	0.9010	0.9571	0.7234	0.6807
210.0000	1.3823	1.4831	0.8999	0.9568	0.7161	0.6722
220.0000	1.4431	1.5575	0.8998	0.9547	0.7102	0.6599
230.0000	1.6943	1.8609	0.8978	0.9523	0.7047	0.6483
240.0000	2.3293	2.6309	0.8966	0.9513	0.6997	0.6400
250.0000	3.0602	3.5797	0.8955	0.9498	0.6917	0.6286
260.0000	3.5679	4.3778	0.8947	0.9472	0.6790	0.6195
270.0000	3.6863	4.6889	0.8921	0.9382	0.6657	0.6090
280.0000	3.6872	4.6899	0.8902	0.9348	0.6596	0.6016
290.0000	3.6894	4.6928	0.8897	0.9293	0.6477	0.5919
300.0000	3.6916	4.6937	0.8884	0.9252	0.6380	0.5831
310.0000	3.6923	4.6937	0.8876	0.9199	0.6284	0.5748
320.0000	3.6949	4.6957	0.8873	0.9022	0.6194	0.5674
330.0000	3.6960	4.6966	0.8868	0.8930	0.6155	0.5603
340.0000	3.6973	4.6966	0.8841	0.8866	0.6117	0.5524
350.0000	3.6973	4.7015	0.8826	0.8836	0.6042	0.5470
360.0000	3.6974	4.8454	0.8806	0.8829	0.6018	0.5410
370.0000	3.6975	4.9768	0.8789	0.8801	0.5991	0.5364
380.0000	3.6976	4.9768	0.8783	0.8769	0.5942	0.5307
390.0000	3.6992	4.9807	0.8767	0.8746	0.5921	0.5256
400.0000	3.6993	4.9807	0.8765	0.8729	0.5926	0.5211
410.0000	3.6993	4.9807	0.8758	0.8720	0.5866	0.5176
420.0000	3.6997	4.9816	0.8735	0.8710	0.5877	0.5131
430.0000	3.7000	4.9816	0.8725	0.8703	0.5837	0.5085
440.0000	3.7005	4.9816	0.8723	0.8701	0.5836	0.5057
450.0000	3.7009	4.9826	0.8717	0.8669	0.5808	0.5023
460.0000	3.7009	4.9845	0.8699	0.8661	0.5789	0.4989
470.0000	3.7015	4.9845	0.8689	0.8660	0.5767	0.4969
480.0000	3.7017	4.9894	0.8673	0.8656	0.5771	0.4932
490.0000	3.7024	4.9894	0.8644	0.8641	0.5750	0.4898
500.0000	3.7025	4.9903	0.8631	0.8631	0.5753	0.4878
510.0000	3.7028	5.0010	0.8619	0.8618	0.5775	0.4861
520.0000	3.7030	5.0116	0.8613	0.8581	0.5748	0.4829
530.0000	3.7030	5.0348	0.8607	0.8541	0.5734	0.4804

540.0000	3.7038	5.0358	0.8606	0.8472	0.5724	0.4775
550.0000	3.7038	5.0560	0.8605	0.8458	0.5711	0.4761
560.0000	3.7044	5.0715	0.8593	0.8363	0.5682	0.4733
570.0000	3.7045	5.1015	0.8581	0.8342	0.5726	0.4713
580.0000	3.7046	5.1034	0.8534	0.8319	0.5706	0.4696
590.0000	3.7047	5.1188	0.8517	0.8280	0.5689	0.4667
600.0000	3.7049	5.1198	0.8502	0.8261	0.5685	0.4651

Hg^{2+}	5*10^{-4} mol/L					
t (s)	RBH-MFI	RBH-R	Calcein-MFI	Calcein-G	MDAC-MFI	MDAC-B
0.0000	1.0000	1.0000	1.0000	1.0000	1.0000	1.0000
10.0000	1.0142	1.0101	0.9918	0.9931	0.9461	0.9472
20.0000	1.0252	1.0202	0.9997	1.0119	0.9126	0.9105
30.0000	1.0325	1.0377	0.9772	0.9887	0.8906	0.8854
40.0000	1.0390	1.0460	0.9526	0.9551	0.8728	0.8682
50.0000	1.0461	1.0570	0.9392	0.9376	0.8603	0.8560
60.0000	1.0533	1.0653	0.9207	0.9151	0.8489	0.8459
70.0000	1.0592	1.0763	0.9035	0.9030	0.8402	0.8370
80.0000	1.0643	1.0855	0.8920	0.8845	0.8331	0.8304
90.0000	1.0659	1.0919	0.8806	0.8709	0.8245	0.8226
100.0000	1.0910	1.1085	0.8724	0.8650	0.8178	0.8165
110.0000	1.1060	1.1158	0.8624	0.8628	0.8120	0.8109
120.0000	1.1120	1.1498	0.8557	0.8542	0.8070	0.8059
130.0000	1.1302	1.1737	0.8471	0.8551	0.8023	0.8015
140.0000	1.1401	1.1958	0.8346	0.8351	0.7982	0.7970
150.0000	1.1597	1.2206	0.8270	0.8347	0.7945	0.7931
160.0000	1.1795	1.2518	0.8138	0.8095	0.7907	0.7892
170.0000	1.2064	1.2868	0.8068	0.8087	0.7878	0.7870
180.0000	1.2353	1.3226	0.7892	0.7878	0.7837	0.7831
190.0000	1.2663	1.3585	0.7827	0.7915	0.7791	0.7786
200.0000	1.2970	1.4026	0.7686	0.7742	0.7733	0.7720
210.0000	1.3348	1.4540	0.7604	0.7564	0.7657	0.7642
220.0000	1.3781	1.5331	0.7416	0.7443	0.7610	0.7564
230.0000	1.4429	1.7040	0.7307	0.7337	0.7535	0.7469
240.0000	1.5851	2.0781	0.7237	0.7206	0.7452	0.7319
250.0000	1.8944	2.5083	0.7227	0.7226	0.7415	0.7264
260.0000	2.2475	2.8566	0.7216	0.7137	0.7398	0.7236
270.0000	2.5312	3.2371	0.7303	0.7438	0.7307	0.7108
280.0000	2.8416	3.6204	0.7449	0.7769	0.7270	0.7063
290.0000	3.1519	3.8621	0.7606	0.7957	0.7210	0.6997
300.0000	3.3472	3.9881	0.7712	0.8100	0.7174	0.6930
310.0000	3.4424	4.0487	0.7760	0.7932	0.7136	0.6902
320.0000	3.4811	4.0882	0.7848	0.8058	0.7085	0.6835
330.0000	3.5114	4.0892	0.7923	0.8147	0.7059	0.6813
340.0000	3.5114	4.0938	0.7946	0.8176	0.7026	0.6763
350.0000	3.5126	4.1020	0.8005	0.8260	0.7029	0.6769
360.0000	3.5171	4.1057	0.8025	0.8277	0.7029	0.6774
370.0000	3.5240	4.0947	0.8038	0.8305	0.7044	0.6802
380.0000	3.5151	4.0984	0.8066	0.8347	0.6997	0.6735
390.0000	3.5177	4.0938	0.8056	0.8332	0.6977	0.6719
400.0000	3.5139	4.0864	0.8074	0.8351	0.7007	0.6746
410.0000	3.5077	4.0754	0.8060	0.8351	0.6982	0.6741
420.0000	3.4985	4.0800	0.8055	0.8334	0.7016	0.6730
430.0000	3.5028	4.0800	0.8085	0.8324	0.6974	0.6763
440.0000	3.4995	4.0772	0.8060	0.8361	0.6984	0.6713
450.0000	3.5003	4.0754	0.8076	0.8337	0.6982	0.6719
460.0000	3.4977	4.0745	0.8072	0.8351	0.6991	0.6730
470.0000	3.4979	4.0708	0.8070	0.8356	0.6952	0.6741
480.0000	3.4952	4.0680	0.8058	0.8356	0.6945	0.6669

490.0000	3.4912	4.0699	0.8078	0.8344	0.7014	0.6669
500.0000	3.4941	4.0625	0.8088	0.8376	0.7014	0.6769
510.0000	3.4880	4.0643	0.8089	0.8381	0.7040	0.6830
520.0000	3.4899	4.0653	0.8072	0.8403	0.7063	0.6891
530.0000	3.4896	4.0570	0.8076	0.8406	0.7094	0.6969
540.0000	3.4817	4.0588	0.8081	0.8391	0.7109	0.6997
550.0000	3.4851	4.0478	0.8089	0.8406	0.7107	0.7002
560.0000	3.4760	4.0469	0.8079	0.8421	0.7097	0.7002
570.0000	3.4754	4.0377	0.8104	0.8435	0.7104	0.7019
580.0000	3.4690	4.0313	0.8092	0.8428	0.7088	0.7008
590.0000	3.4602	4.0340	0.8094	0.8472	0.7145	0.7058
600.0000	3.4647	4.0340	0.8081	0.8463	0.7148	0.7054

Hg^{2+}	$2*10^{-4}$ mol/L					
t (s)	RBH-MFI	RBH-R	Calcein-MFI	Calcein-G	MDAC-MFI	MDAC-B
0.0000	1.0000	1.0000	1.0000	1.0000	1.0000	1.0000
10.0000	1.0037	1.0187	0.9574	0.9463	0.9896	0.9883
20.0000	1.0080	1.0326	0.9326	0.9141	0.9827	0.9799
30.0000	1.0129	1.0497	0.9167	0.8925	0.9753	0.9716
40.0000	1.0191	1.0653	0.9042	0.8756	0.9631	0.9633
50.0000	1.0245	1.0800	0.8941	0.8622	0.9579	0.9564
60.0000	1.0313	1.0793	0.8865	0.8529	0.9545	0.9511
70.0000	1.0382	1.0870	0.8804	0.8460	0.9494	0.9462
80.0000	1.0494	1.1002	0.8730	0.8375	0.9478	0.9393
90.0000	1.0593	1.1119	0.8646	0.8282	0.9479	0.9378
100.0000	1.0705	1.1259	0.8529	0.8149	0.9471	0.9378
110.0000	1.0805	1.1438	0.8343	0.8077	0.9440	0.9359
120.0000	1.0946	1.1562	0.8283	0.8038	0.9440	0.9329
130.0000	1.1076	1.1686	0.8210	0.8064	0.9404	0.9325
140.0000	1.1209	1.1787	0.8094	0.8115	0.9407	0.9281
150.0000	1.1365	1.1943	0.8027	0.8259	0.9361	0.9285
160.0000	1.1544	1.2082	0.7976	0.8344	0.9333	0.9227
170.0000	1.1702	1.2199	0.7897	0.8419	0.9294	0.9188
180.0000	1.1888	1.2347	0.7749	0.8434	0.9294	0.9143
190.0000	1.2099	1.2471	0.7689	0.8218	0.9278	0.9124
200.0000	1.2298	1.2572	0.7812	0.7989	0.9244	0.9085
210.0000	1.2546	1.2704	0.7812	0.7881	0.9205	0.9041
220.0000	1.2792	1.2821	0.7748	0.7809	0.9164	0.8992
230.0000	1.3043	1.2992	0.7679	0.7730	0.9199	0.9031
240.0000	1.3314	1.3124	0.7644	0.7691	0.9210	0.9046
250.0000	1.3314	1.3232	0.7599	0.7642	0.9193	0.9026
260.0000	1.3611	1.3326	0.7501	0.7519	0.9137	0.8977
270.0000	1.3913	1.3465	0.7121	0.7017	0.9134	0.8972
280.0000	1.4201	1.3582	0.6956	0.6724	0.9107	0.8933
290.0000	1.4547	1.3722	0.6920	0.6722	0.9076	0.8909
300.0000	1.5000	1.3831	0.6896	0.6750	0.9068	0.8904
310.0000	1.6010	1.3971	0.6911	0.6827	0.9030	0.8855
320.0000	1.8680	1.4017	0.7028	0.7012	0.8991	0.8816
330.0000	2.1563	1.4180	0.7139	0.7187	0.8947	0.8762
340.0000	2.4548	1.4297	0.7228	0.7308	0.8717	0.8492
350.0000	2.7623	1.4421	0.7327	0.7436	0.8546	0.8297
360.0000	3.0987	1.4631	0.7414	0.7565	0.8444	0.8174
370.0000	3.2522	1.5097	0.7489	0.7686	0.8461	0.8174
380.0000	3.4251	1.6387	0.7573	0.7802	0.8438	0.8155
390.0000	3.4471	1.9922	0.7606	0.7874	0.8488	0.8213
400.0000	3.4597	2.3295	0.7697	0.7848	0.8522	0.8253
410.0000	3.4904	2.6752	0.7731	0.7899	0.8462	0.8174
420.0000	3.4586	2.9883	0.7757	0.7951	0.8408	0.8116
430.0000	3.4692	3.2277	0.7768	0.7969	0.8383	0.8096

440.0000	3.4799	3.3955	0.7777	0.7987	0.8381	0.8101
450.0000	3.4914	3.4926	0.7785	0.8002	0.8329	0.8032
460.0000	3.4465	3.5416	0.7799	0.8020	0.8263	0.7964
470.0000	3.4512	3.5688	0.7800	0.8036	0.8221	0.7925
480.0000	3.4606	3.5626	0.7821	0.8077	0.8243	0.7949
490.0000	3.4598	3.5486	0.7820	0.8082	0.8186	0.7886
500.0000	3.4648	3.5431	0.7805	0.8066	0.8104	0.7797
510.0000	3.4733	3.5136	0.7826	0.8102	0.8110	0.7802
520.0000	3.4738	3.4911	0.7840	0.8126	0.8103	0.7797
530.0000	3.4772	3.4709	0.7833	0.8120	0.8040	0.7729
540.0000	3.4857	3.4600	0.7820	0.8113	0.8002	0.7685
550.0000	3.4456	3.4662	0.7824	0.8128	0.8028	0.7719
560.0000	3.4417	3.4553	0.7819	0.8128	0.7972	0.7665
570.0000	3.4474	3.4343	0.7824	0.8144	0.7902	0.7582
580.0000	3.4531	3.4219	0.7824	0.8144	0.7943	0.7626
590.0000	3.4547	3.4274	0.7825	0.8156	0.7920	0.7607
600.0000	3.4582	3.4196	0.7808	0.8138	0.7851	0.7533
Al^{3+}	$1*10^{-3}$ mol/L					
t (s)	RBH-MFI	RBH-R	Calcein-MFI	Calcein-G	MDAC-MFI	MDAC-B
0.0000	1.0000	1.0000	1.0000	1.0000	1.0000	1.0000
10.0000	1.0279	1.0378	0.9853	0.9770	0.9914	0.9894
20.0000	1.0650	1.0821	0.9825	0.9738	0.9715	0.9846
30.0000	1.0988	1.1215	0.9761	0.9623	0.9553	0.9769
40.0000	1.1397	1.1700	0.9694	0.9518	0.9451	0.9675
50.0000	1.1856	1.2258	0.9663	0.9466	0.9403	0.9616
60.0000	1.2366	1.2833	0.9613	0.9387	0.9373	0.9592
70.0000	1.2980	1.3539	0.9565	0.9324	0.9349	0.9562
80.0000	1.3562	1.4195	0.9538	0.9272	0.9294	0.9503
90.0000	1.4186	1.4926	0.9492	0.9214	0.9255	0.9456
100.0000	1.4902	1.5739	0.9472	0.9178	0.9277	0.9486
110.0000	1.5569	1.6519	0.9436	0.9125	0.9264	0.9474
120.0000	1.6203	1.7250	0.9411	0.9068	0.9216	0.9415
130.0000	1.6792	1.7923	0.9396	0.9057	0.9194	0.9385
140.0000	1.7420	1.8654	0.9341	0.8958	0.9182	0.9379
150.0000	1.7999	1.9302	0.9353	0.9000	0.9180	0.9379
160.0000	1.8587	1.9959	0.9327	0.8963	0.9146	0.9344
170.0000	1.9125	2.0567	0.9305	0.8926	0.9118	0.9314
180.0000	1.9635	2.1158	0.9293	0.8900	0.9120	0.9320
190.0000	2.0085	2.1650	0.9265	0.8853	0.9108	0.9308
200.0000	2.0507	2.2126	0.9266	0.8858	0.9082	0.9296
210.0000	2.0887	2.2570	0.9238	0.8774	0.9104	0.9308
220.0000	2.1239	2.2956	0.9199	0.8680	0.9086	0.9284
230.0000	2.1474	2.3243	0.9219	0.8711	0.9115	0.9308
240.0000	2.1779	2.3580	0.9194	0.8680	0.9108	0.9296
250.0000	2.2029	2.3859	0.9226	0.8722	0.9098	0.9290
260.0000	2.2353	2.4212	0.9252	0.8748	0.9100	0.9279
270.0000	2.2519	2.4392	0.9251	0.8701	0.9089	0.9255
280.0000	2.2827	2.4737	0.9237	0.8654	0.9109	0.9279
290.0000	2.2982	2.4926	0.9258	0.8680	0.9106	0.9273
300.0000	2.3206	2.5172	0.9294	0.8738	0.9092	0.9255
310.0000	2.3345	2.5320	0.9297	0.8722	0.9082	0.9237
320.0000	2.3496	2.5501	0.9291	0.8711	0.9065	0.9214
330.0000	2.3682	2.5723	0.9308	0.8748	0.9068	0.9214
340.0000	2.3829	2.5887	0.9285	0.8717	0.9051	0.9190
350.0000	2.4077	2.6174	0.9280	0.8711	0.9034	0.9160
360.0000	2.4392	2.6535	0.9298	0.8738	0.9004	0.9125
370.0000	2.4770	2.6946	0.9301	0.8759	0.8993	0.9101
380.0000	2.5141	2.7381	0.9292	0.8753	0.8981	0.9078

390.0000	2.5448	2.7718	0.9288	0.8743	0.8952	0.9048
400.0000	2.5723	2.8054	0.9288	0.8743	0.8902	0.8983
410.0000	2.5879	2.8292	0.9280	0.8722	0.8891	0.8947
420.0000	2.6105	2.8588	0.9285	0.8738	0.8858	0.8894
430.0000	2.6270	2.8875	0.9290	0.8743	0.8838	0.8876
440.0000	2.6403	2.9146	0.9294	0.8748	0.8812	0.8817
450.0000	2.6505	2.9384	0.9288	0.8743	0.8728	0.8770
460.0000	2.6649	2.9688	0.9296	0.8748	0.8637	0.8794
470.0000	2.6812	2.9943	0.9328	0.8806	0.8580	0.8811
480.0000	2.6941	3.0189	0.9318	0.8769	0.8578	0.8740
490.0000	2.6998	3.0386	0.9273	0.8706	0.8564	0.8758
500.0000	2.7115	3.0608	0.9281	0.8717	0.8490	0.8616
510.0000	2.7184	3.0821	0.9302	0.8753	0.8370	0.8179
520.0000	2.7159	3.0928	0.9290	0.8727	0.8308	0.8043
530.0000	2.7177	3.1035	0.9291	0.8727	0.8275	0.8001
540.0000	2.7321	3.1174	0.9291	0.8722	0.8301	0.8031
550.0000	2.7525	3.1322	0.9285	0.8706	0.8274	0.7983
560.0000	2.7663	3.1404	0.9300	0.8738	0.8274	0.7978
570.0000	2.7806	3.1552	0.9308	0.8738	0.8246	0.7948
580.0000	2.7927	3.1650	0.9290	0.8706	0.8252	0.7954
590.0000	2.8131	3.1847	0.9293	0.8727	0.8241	0.7948
600.0000	2.8219	3.1913	0.9301	0.8743	0.8224	0.7907

Al^{3+}	$5*10^{-4}$ mol/L					
t (s)	RBH-MFI	RBH-R	Calcein-MFI	Calcein-G	MDAC-MFI	MDAC-B
0.0000	1.0000	1.0000	1.0000	1.0000	1.0000	1.0000
10.0000	1.0161	1.0197	0.9977	1.0047	0.9750	0.9745
20.0000	1.0305	1.0376	0.9937	1.0075	0.9508	0.9485
30.0000	1.0469	1.0582	0.9865	1.0014	0.9328	0.9310
40.0000	1.0622	1.0780	0.9783	1.0005	0.9165	0.9140
50.0000	1.0742	1.0923	0.9722	0.9949	0.9063	0.9028
60.0000	1.0847	1.1084	0.9704	0.9944	0.8911	0.8874
70.0000	1.0961	1.1201	0.9667	0.9921	0.8850	0.8810
80.0000	1.1077	1.1362	0.9635	0.9902	0.8736	0.8699
90.0000	1.1147	1.1452	0.9607	0.9874	0.8658	0.8625
100.0000	1.1265	1.1595	0.9584	0.9860	0.8598	0.8566
110.0000	1.1355	1.1694	0.9568	0.9851	0.8527	0.8492
120.0000	1.1453	1.1819	0.9529	0.9823	0.8465	0.8433
130.0000	1.1540	1.1927	0.9670	0.9828	0.8405	0.8375
140.0000	1.1616	1.2016	0.9724	0.9749	0.8340	0.8311
150.0000	1.1685	1.2097	0.9704	0.9707	0.8263	0.8232
160.0000	1.1772	1.2213	0.9679	0.9669	0.8227	0.8194
170.0000	1.1871	1.2330	0.9648	0.9641	0.8123	0.8094
180.0000	1.1960	1.2446	0.9631	0.9632	0.8089	0.8062
190.0000	1.2049	1.2545	0.9592	0.9595	0.8009	0.7977
200.0000	1.2131	1.2643	0.9556	0.9572	0.7908	0.7876
210.0000	1.2242	1.2787	0.9489	0.9502	0.7844	0.7801
220.0000	1.2349	1.2903	0.9496	0.9455	0.7792	0.7738
230.0000	1.2446	1.3029	0.9458	0.9441	0.7734	0.7679
240.0000	1.2553	1.3136	0.9443	0.9441	0.7715	0.7663
250.0000	1.2656	1.3262	0.9562	0.9469	0.7689	0.7642
260.0000	1.2743	1.3351	0.9572	0.9497	0.7652	0.7610
270.0000	1.2866	1.3495	0.9527	0.9422	0.7568	0.7515
280.0000	1.2973	1.3602	0.9490	0.9339	0.7480	0.7387
290.0000	1.3082	1.3728	0.9483	0.9311	0.7424	0.7313
300.0000	1.3177	1.3844	0.9512	0.9399	0.7429	0.7297
310.0000	1.3332	1.3996	0.9499	0.9357	0.7353	0.7212
320.0000	1.3731	1.4444	0.9493	0.9348	0.7226	0.7069
330.0000	1.4513	1.5287	0.9476	0.9334	0.7212	0.7037

340.0000	1.5327	1.6156	0.9442	0.9311	0.7154	0.6936
350.0000	1.6013	1.6891	0.9447	0.9325	0.7144	0.6893
360.0000	1.6635	1.7527	0.9429	0.9287	0.7121	0.6851
370.0000	1.7178	1.8082	0.9524	0.9320	0.7095	0.6792
380.0000	1.7618	1.8522	0.9542	0.9376	0.7039	0.6739
390.0000	1.8011	1.8907	0.9514	0.9348	0.7044	0.6707
400.0000	1.8341	1.9229	0.9494	0.9357	0.6993	0.6633
410.0000	1.8626	1.9516	0.9484	0.9367	0.6980	0.6601
420.0000	1.8974	1.9848	0.9453	0.9353	0.6950	0.6564
430.0000	1.9251	2.0134	0.9448	0.9362	0.6944	0.6559
440.0000	1.9509	2.0394	0.9439	0.9353	0.6900	0.6506
450.0000	1.9736	2.0618	0.9432	0.9357	0.6883	0.6479
460.0000	2.0000	2.0896	0.9427	0.9348	0.6860	0.6468
470.0000	2.0252	2.1147	0.9396	0.9297	0.6830	0.6415
480.0000	2.0536	2.1407	0.9408	0.9329	0.6936	0.6580
490.0000	2.0737	2.1604	0.9381	0.9292	0.6876	0.6516
500.0000	2.0981	2.1819	0.9380	0.9306	0.6988	0.6697
510.0000	2.1145	2.1971	0.9375	0.9306	0.6888	0.6495
520.0000	2.1331	2.2142	0.9378	0.9311	0.6917	0.6548
530.0000	2.1559	2.2348	0.9360	0.9292	0.6914	0.6564
540.0000	2.1777	2.2554	0.9338	0.9264	0.6842	0.6458
550.0000	2.1951	2.2724	0.9346	0.9273	0.6845	0.6431
560.0000	2.2121	2.2885	0.9342	0.9278	0.6884	0.6522
570.0000	2.2318	2.3056	0.9346	0.9283	0.6855	0.6421
580.0000	2.2487	2.3226	0.9296	0.9204	0.6856	0.6463
590.0000	2.2652	2.3378	0.9316	0.9232	0.6805	0.6368
600.0000	2.2823	2.3548	0.9300	0.9222	0.6838	0.6410

Al^{3+}	$2*10^{-4}$ mol/L					
t (s)	RBH-MFI	RBH-R	Calcein-MFI	Calcein-G	MDAC-MFI	MDAC-B
0.0000	1.0000	1.0000	1.0000	1.0000	1.0000	1.0000
10.0000	1.0074	1.0088	0.9904	0.9963	0.9933	0.9947
20.0000	1.0113	1.0113	0.9840	0.9988	0.9918	0.9888
30.0000	1.0232	1.0214	0.9790	0.9992	0.9903	0.9840
40.0000	1.0243	1.0302	0.9750	0.9942	0.9884	0.9808
50.0000	1.0523	1.0427	0.9733	0.9938	0.9860	0.9771
60.0000	1.0697	1.0540	0.9673	0.9888	0.9814	0.9744
70.0000	1.0878	1.0641	0.9638	0.9851	0.9779	0.9728
80.0000	1.1098	1.0754	0.9623	0.9830	0.9726	0.9686
90.0000	1.1316	1.0892	0.9632	0.9780	0.9684	0.9670
100.0000	1.1697	1.1030	0.9613	0.9726	0.9643	0.9632
110.0000	1.1912	1.1181	0.9607	0.9718	0.9628	0.9600
120.0000	1.2083	1.1344	0.9575	0.9693	0.9589	0.9558
130.0000	1.2438	1.1508	0.9543	0.9660	0.9567	0.9542
140.0000	1.2672	1.1646	0.9519	0.9652	0.9545	0.9515
150.0000	1.2860	1.1834	0.9490	0.9619	0.9509	0.9483
160.0000	1.3053	1.1960	0.9455	0.9581	0.9490	0.9457
170.0000	1.3309	1.2073	0.9447	0.9585	0.9462	0.9430
180.0000	1.3534	1.2173	0.9439	0.9610	0.9419	0.9398
190.0000	1.3742	1.2312	0.9390	0.9544	0.9384	0.9377
200.0000	1.3976	1.2387	0.9366	0.9515	0.9366	0.9339
210.0000	1.4053	1.2475	0.9376	0.9527	0.9343	0.9318
220.0000	1.4147	1.2563	0.9338	0.9478	0.9315	0.9302
230.0000	1.4182	1.2613	0.9295	0.9391	0.9294	0.9270
240.0000	1.4145	1.2676	0.9275	0.9362	0.9280	0.9260
250.0000	1.4452	1.2676	0.9278	0.9366	0.9240	0.9217
260.0000	1.4488	1.2701	0.9254	0.9345	0.9220	0.9244
270.0000	1.4478	1.2714	0.9349	0.9316	0.9191	0.9217
280.0000	1.4478	1.2751	0.9328	0.9308	0.9191	0.9201

290.0000	1.4521	1.2789	0.9330	0.9304	0.9165	0.9180
300.0000	1.4597	1.2776	0.9332	0.9304	0.9155	0.9158
310.0000	1.4525	1.2751	0.9313	0.9270	0.9122	0.9132
320.0000	1.4485	1.2789	0.9364	0.9353	0.9114	0.9105
330.0000	1.4517	1.2802	0.9362	0.9341	0.9077	0.9036
340.0000	1.4542	1.2852	0.9377	0.9374	0.9104	0.9004
350.0000	1.4608	1.2902	0.9391	0.9391	0.9079	0.8998
360.0000	1.4708	1.2977	0.9421	0.9424	0.9055	0.9025
370.0000	1.4827	1.3065	0.9412	0.9415	0.9040	0.8977
380.0000	1.4986	1.3204	0.9429	0.9428	0.9024	0.8977
390.0000	1.5217	1.3367	0.9421	0.9411	0.8989	0.8951
400.0000	1.5497	1.3593	0.9395	0.9362	0.8970	0.8919
410.0000	1.5887	1.3844	0.9417	0.9391	0.8906	0.8897
420.0000	1.6632	1.4083	0.9433	0.9415	0.8879	0.8887
430.0000	1.7043	1.4322	0.9429	0.9391	0.8868	0.8876
440.0000	1.7426	1.4548	0.9447	0.9428	0.8864	0.8855
450.0000	1.7742	1.4749	0.9428	0.9382	0.8828	0.8812
460.0000	1.8000	1.5000	0.9443	0.9403	0.8840	0.8807
470.0000	1.8342	1.5151	0.9460	0.9440	0.8817	0.8785
480.0000	1.8522	1.5327	0.9466	0.9449	0.8786	0.8769
490.0000	1.8744	1.5465	0.9452	0.9411	0.8770	0.8753
500.0000	1.8909	1.5716	0.9463	0.9432	0.8761	0.8753
510.0000	1.9258	1.5817	0.9453	0.9420	0.8747	0.8753
520.0000	1.9349	1.6030	0.9441	0.9395	0.8717	0.8721
530.0000	1.9621	1.6194	0.9438	0.9395	0.8693	0.8668
540.0000	1.9786	1.6344	0.9439	0.9391	0.8680	0.8657
550.0000	1.9786	1.6470	0.9486	0.9457	0.8656	0.8641
560.0000	1.9994	1.6583	0.9461	0.9424	0.8644	0.8631
570.0000	2.0095	1.6746	0.9454	0.9411	0.8626	0.8652
580.0000	2.0230	1.6797	0.9461	0.9424	0.8625	0.8652
590.0000	2.0428	1.6998	0.9456	0.9428	0.8625	0.8626
600.0000	2.0481	1.7098	0.9475	0.9453	0.8594	0.8631
Cr^{3+}	$1*10^{-3}$ mol/L					
t (s)	RBH-MFI	RBH-R	Calcein-MFI	Calcein-G	MDAC-MFI	MDAC-B
0.0000	1.0000	1.0000	1.0000	1.0000	1.0000	1.0000
10.0000	1.0153	1.0109	0.9933	0.9705	0.9972	0.9484
20.0000	1.0291	1.0238	0.9783	0.9470	0.9933	0.9463
30.0000	1.0410	1.0348	0.9730	0.9210	0.9894	0.9435
40.0000	1.0530	1.0467	0.9451	0.8957	0.9854	0.9387
50.0000	1.0654	1.0596	0.9442	0.8768	0.9814	0.9366
60.0000	1.0812	1.0725	0.9372	0.8639	0.9780	0.9339
70.0000	1.0958	1.0874	0.9334	0.8503	0.9749	0.9304
80.0000	1.1136	1.1043	0.9191	0.8368	0.9708	0.9277
90.0000	1.1285	1.1182	0.9205	0.8226	0.9683	0.9249
100.0000	1.1489	1.1380	0.9185	0.8050	0.9642	0.9229
110.0000	1.1655	1.1519	0.8990	0.7896	0.9613	0.9201
120.0000	1.1848	1.1678	0.8932	0.7661	0.9591	0.9180
130.0000	1.2029	1.1867	0.8799	0.7566	0.9558	0.9153
140.0000	1.2231	1.2046	0.8674	0.7531	0.9545	0.9139
150.0000	1.2443	1.2244	0.8475	0.7472	0.9509	0.9119
160.0000	1.2662	1.2433	0.8236	0.7448	0.9504	0.9091
170.0000	1.2860	1.2622	0.8099	0.7454	0.9469	0.9077
180.0000	1.3028	1.2781	0.7980	0.7454	0.9454	0.9050
190.0000	1.3266	1.2989	0.7887	0.7454	0.9442	0.9057
200.0000	1.3451	1.3158	0.7814	0.7454	0.9421	0.9022
210.0000	1.3664	1.3357	0.7780	0.7460	0.9401	0.9008
220.0000	1.3880	1.3545	0.7775	0.7466	0.9390	0.8995
230.0000	1.4059	1.3724	0.7765	0.7466	0.9370	0.8981

240.0000	1.4231	1.3873	0.7695	0.7466	0.9362	0.8967
250.0000	1.4414	1.4052	0.7663	0.7454	0.9340	0.8953
260.0000	1.4604	1.4221	0.7619	0.7431	0.9317	0.8939
270.0000	1.4766	1.4369	0.7586	0.7478	0.9290	0.8926
280.0000	1.4934	1.4518	0.7552	0.7490	0.9270	0.8919
290.0000	1.5111	1.4687	0.7535	0.7484	0.9255	0.8898
300.0000	1.5240	1.4806	0.7537	0.7484	0.9225	0.8871
310.0000	1.5390	1.4955	0.7529	0.7496	0.9197	0.8857
320.0000	1.5546	1.5104	0.7525	0.7496	0.9165	0.8836
330.0000	1.5704	1.5253	0.7536	0.7478	0.9124	0.8822
340.0000	1.5862	1.5392	0.7542	0.7484	0.9065	0.8788
350.0000	1.6041	1.5571	0.7542	0.7496	0.9001	0.8760
360.0000	1.6230	1.5750	0.7547	0.7484	0.8941	0.8726
370.0000	1.6498	1.5998	0.7551	0.7448	0.8881	0.8678
380.0000	1.6829	1.6316	0.7555	0.7484	0.8828	0.8616
390.0000	1.7326	1.6783	0.7555	0.7496	0.8776	0.8561
400.0000	1.8009	1.7408	0.7561	0.7502	0.8714	0.8499
410.0000	1.8806	1.8143	0.7549	0.7478	0.8671	0.8430
420.0000	1.9615	1.8928	0.7548	0.7484	0.8636	0.8347
430.0000	2.0442	1.9742	0.7534	0.7490	0.8608	0.8251
440.0000	2.1168	2.0487	0.7568	0.7502	0.8575	0.8154
450.0000	2.1824	2.1182	0.7577	0.7490	0.8554	0.8072
460.0000	2.2471	2.1847	0.7574	0.7496	0.8524	0.8010
470.0000	2.2968	2.2373	0.7576	0.7484	0.8503	0.7927
480.0000	2.3398	2.2840	0.7580	0.7496	0.8491	0.7865
490.0000	2.3782	2.3247	0.7580	0.7484	0.8472	0.7817
500.0000	2.4148	2.3654	0.7542	0.7472	0.8461	0.7769
510.0000	2.4446	2.3982	0.7531	0.7478	0.8444	0.7741
520.0000	2.4757	2.4290	0.7539	0.7478	0.8441	0.7727
530.0000	2.5045	2.4598	0.7547	0.7478	0.8416	0.7700
540.0000	2.5332	2.4876	0.7536	0.7472	0.8407	0.7672
550.0000	2.5556	2.5124	0.7508	0.7472	0.8399	0.7658
560.0000	2.5726	2.5283	0.7536	0.7478	0.8390	0.7638
570.0000	2.5865	2.5452	0.7543	0.7466	0.8386	0.7624
580.0000	2.5885	2.5492	0.7543	0.7472	0.8383	0.7617
590.0000	2.6100	2.5710	0.7525	0.7454	0.8373	0.7596
600.0000	2.6271	2.5889	0.7501	0.7460	0.8364	0.7596

Cr^{3+}	$5*10^{-4}$ mol/L					
t (s)	RBH-MFI	RBH-R	Calcein-MFI	Calcein-G	MDAC-MFI	MDAC-B
0.0000	1.0000	1.0000	1.0000	1.0000	1.0000	1.0000
10.0000	1.0090	1.0046	0.9951	0.9944	0.9704	0.9786
20.0000	1.0125	1.0081	0.9869	0.9807	0.9498	0.9585
30.0000	1.0154	1.0104	0.9686	0.9695	0.9355	0.9424
40.0000	1.0176	1.0128	0.9635	0.9614	0.9302	0.9294
50.0000	1.0193	1.0151	0.9636	0.9571	0.9219	0.9181
60.0000	1.0223	1.0185	0.9574	0.9502	0.9181	0.9104
70.0000	1.0184	1.0209	0.9551	0.9481	0.9106	0.9015
80.0000	1.0228	1.0232	0.9395	0.9390	0.9030	0.8944
90.0000	1.0273	1.0278	0.9323	0.9266	0.8961	0.8884
100.0000	1.0301	1.0313	0.9299	0.9184	0.8904	0.8807
110.0000	1.0416	1.0348	0.9205	0.9146	0.8872	0.8760
120.0000	1.0466	1.0406	0.9183	0.9137	0.8893	0.8694
130.0000	1.0503	1.0440	0.9173	0.9150	0.8876	0.8647
140.0000	1.0544	1.0487	0.9117	0.9133	0.8853	0.8605
150.0000	1.0585	1.0533	0.9148	0.9090	0.8825	0.8570
160.0000	1.0643	1.0591	0.9140	0.9064	0.8817	0.8522
170.0000	1.0682	1.0649	0.9125	0.8922	0.8789	0.8499
180.0000	1.0719	1.0707	0.9152	0.8879	0.8776	0.8451

190.0000	1.0735	1.0765	0.9137	0.8806	0.8718	0.8415
200.0000	1.0776	1.0834	0.9079	0.8769	0.8695	0.8398
210.0000	1.0817	1.0904	0.9075	0.8659	0.8692	0.8356
220.0000	1.0899	1.0950	0.9048	0.8544	0.8616	0.8344
230.0000	1.0942	1.1020	0.9021	0.8514	0.8581	0.8297
240.0000	1.0989	1.1089	0.8970	0.8484	0.8561	0.8285
250.0000	1.1034	1.1147	0.8920	0.8338	0.8587	0.8261
260.0000	1.1056	1.1194	0.8874	0.8205	0.8535	0.8261
270.0000	1.1089	1.1240	0.8773	0.8167	0.8515	0.8214
280.0000	1.1178	1.1333	0.8713	0.8055	0.8537	0.8202
290.0000	1.1317	1.1379	0.8618	0.7999	0.8558	0.8154
300.0000	1.1344	1.1448	0.8517	0.7913	0.8484	0.8113
310.0000	1.1392	1.1506	0.8485	0.7879	0.8473	0.8101
320.0000	1.1456	1.1588	0.8396	0.7827	0.8536	0.8083
330.0000	1.1525	1.1669	0.8369	0.7789	0.8471	0.8036
340.0000	1.1608	1.1750	0.8280	0.7772	0.8449	0.8000
350.0000	1.1717	1.1889	0.8163	0.7750	0.8494	0.7941
360.0000	1.1889	1.2097	0.8071	0.7742	0.8480	0.7875
370.0000	1.2122	1.2422	0.8047	0.7742	0.8430	0.7828
380.0000	1.2800	1.2932	0.7973	0.7754	0.8429	0.7769
390.0000	1.3405	1.3569	0.7947	0.7767	0.8479	0.7715
400.0000	1.4052	1.4311	0.7857	0.7754	0.8436	0.7656
410.0000	1.4773	1.5099	0.7955	0.7733	0.8408	0.7602
420.0000	1.5419	1.5759	0.7842	0.7729	0.8385	0.7561
430.0000	1.5968	1.6362	0.7883	0.7754	0.8386	0.7555
440.0000	1.6375	1.6825	0.7835	0.7716	0.8321	0.7543
450.0000	1.6578	1.7173	0.7801	0.7699	0.8295	0.7561
460.0000	1.6864	1.7509	0.7791	0.7712	0.8287	0.7561
470.0000	1.7218	1.7903	0.7714	0.7643	0.8261	0.7555
480.0000	1.7471	1.8192	0.7630	0.7639	0.8232	0.7549
490.0000	1.7746	1.8505	0.7672	0.7630	0.8238	0.7543
500.0000	1.7995	1.8795	0.7680	0.7613	0.8256	0.7537
510.0000	1.8246	1.9073	0.7677	0.7608	0.8239	0.7537
520.0000	1.8497	1.9363	0.7665	0.7604	0.8253	0.7531
530.0000	1.8769	1.9664	0.7662	0.7596	0.8254	0.7525
540.0000	1.8973	1.9896	0.7639	0.7596	0.8260	0.7519
550.0000	1.9135	2.0093	0.7612	0.7578	0.8232	0.7507
560.0000	1.9324	2.0301	0.7603	0.7583	0.8235	0.7496
570.0000	1.9508	2.0498	0.7612	0.7570	0.8244	0.7496
580.0000	1.9733	2.0753	0.7593	0.7471	0.8234	0.7484
590.0000	1.9940	2.0985	0.7560	0.7462	0.8241	0.7472
600.0000	2.0085	2.1136	0.7539	0.7458	0.8223	0.7472

Cr^{3+}	$2*10^{-4}$ mol/L					
t (s)	RBH-MFI	RBH-R	Calcein-MFI	Calcein-G	MDAC-MFI	MDAC-B
0.0000	1.0000	1.0000	1.0000	1.0000	1.0000	1.0000
10.0000	1.0002	1.0035	0.9857	0.9911	0.9873	0.9807
20.0000	1.0021	1.0046	0.9813	0.9772	0.9840	0.9585
30.0000	1.0050	1.0070	0.9736	0.9728	0.9749	0.9303
40.0000	1.0097	0.9849	0.9649	0.9673	0.9653	0.9111
50.0000	1.0136	0.9849	0.9603	0.9609	0.9499	0.8977
60.0000	1.0171	0.9861	0.9569	0.9520	0.9310	0.8873
70.0000	1.0206	0.9873	0.9555	0.9396	0.9106	0.8821
80.0000	1.0211	0.9907	0.9522	0.9381	0.8947	0.8777
90.0000	1.0297	0.9942	0.9508	0.9262	0.8828	0.8666
100.0000	1.0341	0.9977	0.9508	0.9257	0.8735	0.8540
110.0000	1.0315	1.0012	0.9487	0.9178	0.8702	0.8443
120.0000	1.0413	1.0070	0.9475	0.9064	0.8707	0.8421
130.0000	1.0415	1.0139	0.9490	0.9089	0.8663	0.8391

140.0000	1.0401	1.0220	0.9480	0.8970	0.8595	0.8325
150.0000	1.0423	1.0301	0.9480	0.8916	0.8546	0.8258
160.0000	1.0454	1.0359	0.9474	0.8871	0.8556	0.8251
170.0000	1.0567	1.0394	0.9465	0.8826	0.8518	0.8221
180.0000	1.0615	1.0452	0.9467	0.8750	0.8495	0.8184
190.0000	1.0661	1.0487	0.9457	0.8688	0.8460	0.8147
200.0000	1.0708	1.0533	0.9432	0.8643	0.8459	0.8110
210.0000	1.0752	1.0591	0.9430	0.8603	0.8452	0.8043
220.0000	1.0798	1.0637	0.9428	0.8549	0.8440	0.7999
230.0000	1.0844	1.0695	0.9427	0.8509	0.8423	0.7947
240.0000	1.0916	1.0753	0.9333	0.8450	0.8396	0.7924
250.0000	1.0962	1.0800	0.9390	0.8405	0.8363	0.7902
260.0000	1.1036	1.0858	0.9401	0.8385	0.8341	0.7858
270.0000	1.1092	1.0915	0.9405	0.8346	0.8321	0.7828
280.0000	1.1584	1.0973	0.9419	0.8331	0.8306	0.7813
290.0000	1.1671	1.1031	0.9416	0.8301	0.8293	0.7806
300.0000	1.1770	1.1112	0.9389	0.8252	0.8268	0.7791
310.0000	1.1847	1.1182	0.9298	0.8222	0.8255	0.7769
320.0000	1.1929	1.1251	0.9168	0.8182	0.8247	0.7761
330.0000	1.2028	1.1309	0.8999	0.8148	0.8236	0.7739
340.0000	1.2106	1.1402	0.8831	0.8073	0.8229	0.7732
350.0000	1.2207	1.1460	0.8684	0.7945	0.8210	0.7724
360.0000	1.2264	1.1530	0.8580	0.7915	0.8211	0.7717
370.0000	1.2341	1.1611	0.8504	0.7880	0.8195	0.7717
380.0000	1.2404	1.1680	0.8436	0.7816	0.8190	0.7709
390.0000	1.2478	1.1761	0.8373	0.7895	0.8181	0.7702
400.0000	1.2524	1.1808	0.8093	0.7846	0.8172	0.7702
410.0000	1.2565	1.1889	0.8012	0.7742	0.8179	0.7702
420.0000	1.2617	1.1935	0.7960	0.7761	0.8167	0.7724
430.0000	1.2667	1.2005	0.7874	0.7766	0.8160	0.7761
440.0000	1.2709	1.2039	0.7821	0.7742	0.8159	0.7776
450.0000	1.2747	1.2086	0.7784	0.7766	0.8161	0.7761
460.0000	1.2809	1.2121	0.7795	0.7732	0.8178	0.7784
470.0000	1.2898	1.2167	0.7780	0.7667	0.8189	0.7806
480.0000	1.3053	1.2202	0.7751	0.7633	0.8195	0.7806
490.0000	1.3287	1.2248	0.7747	0.7628	0.8189	0.7776
500.0000	1.3732	1.2294	0.7766	0.7657	0.8202	0.7761
510.0000	1.4259	1.2375	0.7753	0.7583	0.8201	0.7761
520.0000	1.4767	1.2503	0.7758	0.7529	0.8201	0.7791
530.0000	1.5159	1.2712	0.7756	0.7479	0.8184	0.7798
540.0000	1.5491	1.3071	0.7734	0.7524	0.8181	0.7813
550.0000	1.5785	1.3511	0.7727	0.7454	0.8182	0.7821
560.0000	1.6028	1.3940	0.7693	0.7425	0.8193	0.7806
570.0000	1.6242	1.4299	0.7701	0.7434	0.8196	0.7769
580.0000	1.6657	1.4600	0.7695	0.7474	0.8204	0.7717
590.0000	1.7236	1.4878	0.7678	0.7370	0.8203	0.7695
600.0000	1.7953	1.5122	0.7692	0.7405	0.8193	0.7695

Fe^{3+}	$1*10^{-3}$ mol/L					
t (s)	RBH-MFI	RBH-R	Calcein-MFI	Calcein-G	MDAC-MFI	MDAC-B
0.0000	1.0000	1.0000	1.0000	1.0000	1.0000	1.0000
10.0000	1.0075	1.0115	0.9970	0.9921	1.0003	1.0056
20.0000	1.0137	1.0161	0.9926	0.9820	0.9869	0.9979
30.0000	1.0199	1.0242	0.9920	0.9786	0.9752	0.9902
40.0000	1.0274	1.0357	0.9860	0.9684	0.9665	0.9847
50.0000	1.0336	1.0403	0.9825	0.9628	0.9577	0.9791
60.0000	1.0410	1.0506	0.9816	0.9605	0.9519	0.9749
70.0000	1.0485	1.0587	0.9771	0.9527	0.9436	0.9686
80.0000	1.0585	1.0644	0.9728	0.9448	0.9385	0.9651

90.0000	1.0672	1.0656	0.9706	0.9414	0.9331	0.9616
100.0000	1.0771	1.0714	0.9671	0.9357	0.9284	0.9574
110.0000	1.0858	1.0737	0.9644	0.9335	0.9244	0.9546
120.0000	1.0933	1.0794	0.9609	0.9279	0.9211	0.9526
130.0000	1.1032	1.0852	0.9548	0.9188	0.9182	0.9505
140.0000	1.1119	1.0875	0.9544	0.9177	0.9075	0.9484
150.0000	1.1219	1.0944	0.9531	0.9155	0.9042	0.9428
160.0000	1.1331	1.0990	0.9503	0.9109	0.9021	0.9407
170.0000	1.1430	1.1047	0.9481	0.9098	0.8985	0.9393
180.0000	1.1567	1.1105	0.9485	0.9098	0.8969	0.9365
190.0000	1.1679	1.1139	0.9483	0.9087	0.8944	0.9351
200.0000	1.1791	1.1185	0.9446	0.9042	0.8926	0.9337
210.0000	1.1928	1.1243	0.9433	0.9008	0.8906	0.9323
220.0000	1.2040	1.1300	0.9408	0.8952	0.8883	0.9309
230.0000	1.2202	1.1358	0.9369	0.8884	0.8820	0.9295
240.0000	1.2338	1.1427	0.9353	0.8861	0.8745	0.9253
250.0000	1.2500	1.1519	0.9337	0.8839	0.8686	0.9177
260.0000	1.2649	1.1600	0.9306	0.8760	0.8593	0.9135
270.0000	1.2836	1.1703	0.9279	0.8715	0.8530	0.9058
280.0000	1.3022	1.1841	0.9259	0.8681	0.8489	0.9002
290.0000	1.3197	1.2163	0.9184	0.8533	0.8500	0.8960
300.0000	1.3358	1.2658	0.9126	0.8467	0.8458	0.8953
310.0000	1.3532	1.3441	0.9106	0.8422	0.8399	0.8877
320.0000	1.3694	1.4626	0.9095	0.8422	0.8324	0.8765
330.0000	1.3868	1.5972	0.9078	0.8410	0.8228	0.8639
340.0000	1.4042	1.7261	0.9014	0.8320	0.8137	0.8486
350.0000	1.4216	1.8435	0.9021	0.8332	0.8036	0.8339
360.0000	1.4415	1.9425	0.8999	0.8298	0.7981	0.8172
370.0000	1.4602	2.0265	0.8991	0.8264	0.7981	0.8088
380.0000	1.4764	2.1036	0.8970	0.8230	0.7988	0.8067
390.0000	1.4925	2.1703	0.8959	0.8207	0.7968	0.8039
400.0000	1.5100	2.2244	0.8947	0.8174	0.7953	0.7997
410.0000	1.5299	2.2762	0.8940	0.8162	0.7958	0.7983
420.0000	1.5510	2.3073	0.8939	0.8151	0.7959	0.7962
430.0000	1.5771	2.3027	0.8934	0.8129	0.7970	0.7948
440.0000	1.6144	2.3245	0.8925	0.8095	0.7976	0.7934
450.0000	1.6629	2.3544	0.8906	0.8061	0.7986	0.7927
460.0000	1.7139	2.3867	0.8903	0.8050	0.8018	0.7941
470.0000	1.7624	2.4039	0.8897	0.8050	0.8039	0.7948
480.0000	1.8147	2.4189	0.8886	0.8027	0.8057	0.7955
490.0000	1.8657	2.4304	0.8889	0.8016	0.8087	0.7969
500.0000	1.9216	2.4488	0.8886	0.8005	0.8127	0.7990
510.0000	1.9813	2.4546	0.8872	0.7993	0.8170	0.8018
520.0000	2.0373	2.4695	0.8853	0.7948	0.8189	0.8018
530.0000	2.0945	2.4810	0.8859	0.7959	0.8215	0.8032
540.0000	2.1418	2.4902	0.8853	0.7959	0.8243	0.8046
550.0000	2.1903	2.4971	0.8841	0.7937	0.8299	0.8074
560.0000	2.2326	2.5006	0.8844	0.7948	0.8323	0.8095
570.0000	2.2649	2.5109	0.8830	0.7926	0.8347	0.8102
580.0000	2.3072	2.5167	0.8822	0.7914	0.8375	0.8116
590.0000	2.3396	2.5293	0.8812	0.7903	0.8403	0.8130
600.0000	2.3744	2.5351	0.8807	0.7903	0.8444	0.8151
Fe^{3+}	5*10^{-4} mol/L					
t (s)	RBH-MFI	RBH-R	Calcein-MFI	Calcein-G	MDAC-MFI	MDAC-B
0.0000	1.0000	1.0000	1.0000	1.0000	1.0000	1.0000
10.0000	1.0108	1.0075	0.9971	0.9920	0.9776	0.9784
20.0000	1.0166	1.0137	0.9949	0.9855	0.9588	0.9557
30.0000	1.0229	1.0199	0.9899	0.9754	0.9450	0.9387

40.0000	1.0298	1.0274	0.9767	0.9694	0.9325	0.9245
50.0000	1.0362	1.0336	0.9816	0.9639	0.9211	0.9126
60.0000	1.0436	1.0410	0.9804	0.9559	0.9124	0.9047
70.0000	1.0506	1.0485	0.9735	0.9473	0.9007	0.8944
80.0000	1.0594	1.0585	0.9732	0.9418	0.8910	0.8865
90.0000	1.0675	1.0672	0.9617	0.9328	0.8820	0.8786
100.0000	1.0762	1.0771	0.9682	0.9267	0.8716	0.8695
110.0000	1.0858	1.0858	0.9626	0.9192	0.8643	0.8638
120.0000	1.0940	1.0933	0.9581	0.9137	0.8560	0.8564
130.0000	1.1040	1.1032	0.9529	0.9062	0.8500	0.8513
140.0000	1.1126	1.1119	0.9479	0.9017	0.8451	0.8462
150.0000	1.1229	1.1219	0.9555	0.8976	0.8395	0.8411
160.0000	1.1331	1.1331	0.9529	0.8971	0.8352	0.8366
170.0000	1.1436	1.1430	0.9555	0.8941	0.8321	0.8337
180.0000	1.1554	1.1567	0.9489	0.8911	0.8266	0.8280
190.0000	1.1664	1.1679	0.9479	0.8901	0.8239	0.8252
200.0000	1.1770	1.1791	0.9411	0.8876	0.8214	0.8229
210.0000	1.1884	1.1928	0.9386	0.8861	0.8165	0.8178
220.0000	1.2005	1.2040	0.9346	0.8846	0.8158	0.8173
230.0000	1.2129	1.2202	0.9323	0.8801	0.8111	0.8122
240.0000	1.2262	1.2338	0.9289	0.8786	0.8097	0.8110
250.0000	1.2397	1.2500	0.9262	0.8761	0.8072	0.8082
260.0000	1.2535	1.2649	0.9243	0.8731	0.8069	0.8082
270.0000	1.2677	1.2836	0.9220	0.8711	0.8026	0.8031
280.0000	1.2814	1.3022	0.9211	0.8701	0.8012	0.8019
290.0000	1.2958	1.3197	0.9187	0.8706	0.7973	0.7968
300.0000	1.3110	1.3358	0.9173	0.8695	0.7939	0.7934
310.0000	1.3271	1.3532	0.9159	0.8690	0.7926	0.7923
320.0000	1.3427	1.3694	0.9155	0.8680	0.7905	0.7894
330.0000	1.3590	1.3868	0.9132	0.8675	0.7868	0.7849
340.0000	1.3757	1.4042	0.9125	0.8650	0.7833	0.7809
350.0000	1.3925	1.4216	0.9135	0.8630	0.7784	0.7753
360.0000	1.4104	1.4415	0.9129	0.8660	0.7728	0.7684
370.0000	1.4329	1.4602	0.9146	0.8635	0.7728	0.7628
380.0000	1.4495	1.4764	0.9147	0.8530	0.7680	0.7571
390.0000	1.4657	1.4925	0.9138	0.8540	0.7622	0.7509
400.0000	1.4836	1.5100	0.9139	0.8520	0.7569	0.7452
410.0000	1.5017	1.5299	0.9132	0.8520	0.7514	0.7395
420.0000	1.5219	1.5510	0.9105	0.8515	0.7463	0.7327
430.0000	1.5463	1.5771	0.9109	0.8490	0.7412	0.7259
440.0000	1.5795	1.6144	0.9099	0.8470	0.7362	0.7174
450.0000	1.6228	1.6629	0.9100	0.8475	0.7311	0.7072
460.0000	1.6655	1.7139	0.9091	0.8460	0.7253	0.6975
470.0000	1.7079	1.7624	0.9081	0.8460	0.7200	0.6884
480.0000	1.7517	1.8147	0.9082	0.8445	0.7162	0.6799
490.0000	1.7917	1.8657	0.9076	0.8425	0.7129	0.6742
500.0000	1.8347	1.9216	0.9070	0.8425	0.7118	0.6720
510.0000	1.8818	1.9813	0.9061	0.8420	0.7113	0.6708
520.0000	1.9270	2.0373	0.9059	0.8399	0.7123	0.6697
530.0000	1.9739	2.0945	0.9058	0.8399	0.7123	0.6703
540.0000	2.0144	2.1418	0.9050	0.8389	0.7141	0.6708
550.0000	2.0545	2.1903	0.9048	0.8394	0.7155	0.6720
560.0000	2.0907	2.2326	0.9040	0.8379	0.7177	0.6725
570.0000	2.1176	2.2649	0.9046	0.8374	0.7194	0.6742
580.0000	2.1534	2.3072	0.9044	0.8364	0.7216	0.6782
590.0000	2.1811	2.3396	0.9042	0.8369	0.7261	0.6805
600.0000	2.2115	2.3744	0.9027	0.8369	0.7294	0.6822

Fe^{3+} $2*10^{-4}$ mol/L

t (s)	RBH-MFI	RBH-R	Calcein-MFI	Calcein-G	MDAC-MFI	MDAC-B
0.0000	1.0000	1.0000	1.0000	1.0000	1.0000	1.0000
10.0000	1.0103	1.0103	0.9975	0.9944	0.9345	0.9457
20.0000	1.0247	1.0215	0.9937	0.9897	0.8960	0.9127
30.0000	1.0359	1.0346	0.9931	0.9873	0.8653	0.8864
40.0000	1.0516	1.0487	0.9899	0.9821	0.8395	0.8588
50.0000	1.0686	1.0646	0.9854	0.9746	0.8214	0.8371
60.0000	1.0838	1.0777	0.9847	0.9732	0.8040	0.8191
70.0000	1.0952	1.0899	0.9847	0.9840	0.7850	0.8020
80.0000	1.1071	1.1049	0.9789	0.9647	0.7716	0.7903
90.0000	1.1180	1.1208	0.9747	0.9558	0.7577	0.7774
100.0000	1.1257	1.1320	0.9729	0.9525	0.7437	0.7632
110.0000	1.1374	1.1508	0.9661	0.9421	0.7331	0.7502
120.0000	1.1549	1.1620	0.9648	0.9403	0.7247	0.7419
130.0000	1.1658	1.1760	0.9611	0.9356	0.7169	0.7343
140.0000	1.1783	1.1882	0.9593	0.9327	0.7095	0.7272
150.0000	1.1884	1.2004	0.9582	0.9313	0.7006	0.7197
160.0000	1.2009	1.2126	0.9566	0.9309	0.6932	0.7130
170.0000	1.2111	1.2238	0.9586	0.9346	0.6836	0.7038
180.0000	1.2194	1.2369	0.9514	0.9243	0.6753	0.6955
190.0000	1.2311	1.2416	0.9509	0.9247	0.6679	0.6871
200.0000	1.2336	1.2594	0.9498	0.9224	0.6622	0.6817
210.0000	1.2509	1.2697	0.9488	0.9224	0.6551	0.6734
220.0000	1.2585	1.2753	0.9475	0.9200	0.6499	0.6675
230.0000	1.2617	1.2884	0.9431	0.9144	0.6458	0.6625
240.0000	1.2732	1.3034	0.9420	0.9135	0.6419	0.6575
250.0000	1.2854	1.3155	0.9401	0.9106	0.6371	0.6516
260.0000	1.2944	1.3249	0.9375	0.9064	0.6302	0.6429
270.0000	1.3014	1.3343	0.9367	0.9059	0.6209	0.6328
280.0000	1.3083	1.3390	0.9364	0.9064	0.6127	0.6245
290.0000	1.3110	1.3521	0.9350	0.9045	0.6085	0.6199
300.0000	1.3214	1.3689	0.9334	0.9036	0.6057	0.6165
310.0000	1.3392	1.3961	0.9303	0.8984	0.6002	0.6103
320.0000	1.3545	1.4242	0.9293	0.8970	0.5941	0.6036
330.0000	1.3791	1.4635	0.9262	0.8928	0.5887	0.5973
340.0000	1.4063	1.5038	0.9241	0.8899	0.5838	0.5902
350.0000	1.4423	1.5365	0.9230	0.8885	0.5815	0.5869
360.0000	1.4805	1.5702	0.9214	0.8871	0.5808	0.5861
370.0000	1.5112	1.5955	0.9193	0.8838	0.5787	0.5831
380.0000	1.5442	1.6302	0.9178	0.8824	0.5726	0.5756
390.0000	1.5681	1.6704	0.9170	0.8815	0.5672	0.5685
400.0000	1.5973	1.7116	0.9167	0.8829	0.5631	0.5631
410.0000	1.6473	1.7509	0.9130	0.8772	0.5634	0.5622
420.0000	1.6700	1.7772	0.9138	0.8791	0.5602	0.5593
430.0000	1.6892	1.8081	0.9122	0.8768	0.5515	0.5476
440.0000	1.7170	1.8408	0.9108	0.8758	0.5464	0.5405
450.0000	1.7429	1.8802	0.9092	0.8735	0.5442	0.5380
460.0000	1.7662	1.9139	0.9088	0.8735	0.5437	0.5384
470.0000	1.7567	1.9485	0.9074	0.8716	0.5414	0.5351
480.0000	1.7379	1.9672	0.9058	0.8707	0.5363	0.5276
490.0000	1.7605	1.9935	0.9053	0.8702	0.5335	0.5234
500.0000	1.8111	2.0084	0.9037	0.8678	0.5325	0.5230
510.0000	1.8486	2.0459	0.9014	0.8655	0.5311	0.5213
520.0000	1.8725	2.0552	0.9012	0.8660	0.5279	0.5163
530.0000	1.8831	2.0655	0.9002	0.8650	0.5255	0.5121
540.0000	1.8838	2.0899	0.8985	0.8622	0.5245	0.5121
550.0000	1.8930	2.0899	0.8978	0.8612	0.5246	0.5121
560.0000	1.8998	2.1086	0.8950	0.8584	0.5223	0.5088

570.0000	1.9073	2.1189	0.8923	0.8537	0.5213	0.5071
580.0000	1.9153	2.1320	0.8930	0.8565	0.5219	0.5084
590.0000	1.9275	2.1442	0.8939	0.8584	0.5209	0.5071
600.0000	1.9344	2.1536	0.8921	0.8565	0.5181	0.5025
Cu^{2+}	$1*10^{-3}$ mol/L					
t (s)	RBH-MFI	RBH-R	Calcein-MFI	Calcein-G	MDAC-MFI	MDAC-B
0.0000	1.0000	1.0000	1.0000	1.0000	1.0000	1.0000
10.0000	1.0195	1.0436	0.9802	0.9397	0.9554	0.9534
20.0000	1.0483	1.0637	0.9771	0.9036	0.9219	0.9180
30.0000	1.0767	1.0994	0.9712	0.9018	0.8855	0.8798
40.0000	1.1132	1.1520	0.9636	0.8861	0.8494	0.8412
50.0000	1.1582	1.1989	0.9590	0.8759	0.8122	0.8018
60.0000	1.2069	1.2279	0.9540	0.8730	0.7764	0.7628
70.0000	1.2657	1.2626	0.9598	0.8654	0.7520	0.7346
80.0000	1.3196	1.3587	0.9428	0.8593	0.7366	0.7139
90.0000	1.3760	1.4168	0.9411	0.8715	0.7221	0.6956
100.0000	1.4341	1.4838	0.9365	0.8430	0.7123	0.6840
110.0000	1.4928	1.5441	0.9348	0.8386	0.7029	0.6733
120.0000	1.5527	1.6101	0.9317	0.8316	0.6950	0.6657
130.0000	1.6155	1.6793	0.9218	0.8217	0.6895	0.6602
140.0000	1.6780	1.7475	0.9182	0.8220	0.6835	0.6530
150.0000	1.7429	1.8168	0.9150	0.8156	0.6792	0.6470
160.0000	1.8058	1.8894	0.9059	0.8281	0.6716	0.6375
170.0000	1.8635	1.9609	0.9001	0.8011	0.6645	0.6287
180.0000	1.9237	2.0369	0.8918	0.7990	0.6567	0.6196
190.0000	1.9817	2.1073	0.8841	0.7938	0.6496	0.6124
200.0000	2.0383	2.1743	0.8756	0.7987	0.6421	0.6041
210.0000	2.0896	2.2447	0.8664	0.7900	0.6414	0.6025
220.0000	2.1416	2.3117	0.8551	0.7760	0.6332	0.5945
230.0000	2.1894	2.3743	0.8457	0.7705	0.6383	0.5993
240.0000	2.2459	2.4358	0.8355	0.7661	0.6430	0.6045
250.0000	2.2700	2.4950	0.8266	0.7527	0.6396	0.6017
260.0000	2.3155	2.5531	0.8181	0.7466	0.6356	0.5977
270.0000	2.3531	2.5911	0.8090	0.7346	0.6323	0.5945
280.0000	2.3818	2.6436	0.7998	0.7253	0.6280	0.5901
290.0000	2.4214	2.6849	0.7931	0.7131	0.6234	0.5846
300.0000	2.4532	2.7207	0.7812	0.7000	0.6194	0.5794
310.0000	2.4822	2.7665	0.7790	0.6863	0.6173	0.5762
320.0000	2.5080	2.8067	0.7738	0.6741	0.6129	0.5714
330.0000	2.5291	2.8425	0.7665	0.6601	0.6091	0.5671
340.0000	2.5537	2.8749	0.7616	0.6496	0.6058	0.5627
350.0000	2.5730	2.9039	0.7568	0.6315	0.6009	0.5575
360.0000	2.5977	2.9330	0.7534	0.6193	0.5981	0.5547
370.0000	2.6160	2.9598	0.7491	0.6076	0.5950	0.5507
380.0000	2.6337	2.9877	0.7439	0.5989	0.5921	0.5480
390.0000	2.6495	3.0112	0.7417	0.5797	0.5900	0.5456
400.0000	2.6637	3.0335	0.7392	0.5808	0.5881	0.5436
410.0000	2.6746	3.0514	0.7361	0.5744	0.5862	0.5408
420.0000	2.6901	3.0715	0.7336	0.5645	0.5839	0.5388
430.0000	2.6995	3.0860	0.7332	0.5590	0.5822	0.5364
440.0000	2.7108	3.1062	0.7300	0.5540	0.5801	0.5340
450.0000	2.7540	3.1196	0.7310	0.5491	0.5776	0.5312
460.0000	2.7625	3.1330	0.7293	0.5433	0.5764	0.5308
470.0000	2.7710	3.1441	0.7286	0.5366	0.5742	0.5281
480.0000	2.7831	3.1564	0.7274	0.5339	0.5724	0.5261
490.0000	2.7864	3.1710	0.7259	0.5313	0.5710	0.5241
500.0000	2.7949	3.1777	0.7242	0.5272	0.5666	0.5205
510.0000	2.8047	3.1877	0.7260	0.5237	0.5635	0.5189

520.0000	2.8123	3.2011	0.7258	0.5249	0.5641	0.5161
530.0000	2.8116	3.2134	0.7260	0.5197	0.5629	0.5169
540.0000	2.8183	3.2145	0.7248	0.5208	0.5586	0.5157
550.0000	2.8230	3.2224	0.7225	0.5188	0.5614	0.5113
560.0000	2.8259	3.2279	0.7239	0.5182	0.5571	0.5133
570.0000	2.8299	3.2335	0.7247	0.5170	0.5588	0.5094
580.0000	2.8284	3.2402	0.7238	0.5153	0.5540	0.5102
590.0000	2.8331	3.2402	0.7232	0.5121	0.5550	0.5058
600.0000	2.8351	3.2458	0.7221	0.5150	0.5500	0.5062

Cu^{2+}	$5*10^{-4}$ mol/L					
t (s)	RBH-MFI	RBH-R	Calcein-MFI	Calcein-G	MDAC-MFI	MDAC-B
0.0000	1.0000	1.0000	1.0000	1.0000	1.0000	1.0000
10.0000	1.0122	1.0205	0.9842	0.9988	0.9841	0.9794
20.0000	1.0501	1.0410	0.9868	0.9857	0.9734	0.9658
30.0000	1.0651	1.0615	0.9726	0.9814	0.9629	0.9520
40.0000	1.0799	1.0763	0.9712	0.9826	0.9520	0.9381
50.0000	1.0912	1.1025	0.9709	0.9820	0.9463	0.9302
60.0000	1.1121	1.1201	0.9642	0.9627	0.9386	0.9201
70.0000	1.1259	1.1343	0.9631	0.9553	0.9303	0.9103
80.0000	1.1358	1.1505	0.9565	0.9546	0.9253	0.9024
90.0000	1.1481	1.1682	0.9534	0.9453	0.9228	0.8979
100.0000	1.1614	1.1852	0.9463	0.9360	0.9159	0.8896
110.0000	1.1753	1.2014	0.9425	0.9298	0.9175	0.8900
120.0000	1.1879	1.2233	0.9373	0.9254	0.9114	0.8818
130.0000	1.2029	1.2481	0.9337	0.9181	0.9097	0.8791
140.0000	1.2217	1.2678	0.9300	0.9127	0.9073	0.8750
150.0000	1.2369	1.2905	0.9263	0.9091	0.9031	0.8701
160.0000	1.2540	1.3152	0.9233	0.9062	0.9021	0.8679
170.0000	1.2729	1.3378	0.9183	0.8937	0.9011	0.8664
180.0000	1.2907	1.3668	0.9150	0.8875	0.8996	0.8641
190.0000	1.3117	1.3929	0.9121	0.8819	0.8978	0.8611
200.0000	1.3321	1.4240	0.9107	0.8813	0.8942	0.8566
210.0000	1.3863	1.4523	0.9104	0.8726	0.8941	0.8559
220.0000	1.4099	1.4876	0.9114	0.8633	0.8924	0.8532
230.0000	1.4389	1.5230	0.9100	0.8583	0.8914	0.8517
240.0000	1.4667	1.5661	0.9082	0.8502	0.8913	0.8514
250.0000	1.5014	1.6290	0.9095	0.8421	0.8900	0.8491
260.0000	1.5507	1.6841	0.9108	0.8409	0.8878	0.8461
270.0000	1.5957	1.7569	0.9026	0.8353	0.8889	0.8465
280.0000	1.6545	1.8198	0.8927	0.8303	0.8854	0.8423
290.0000	1.7054	1.8869	0.8940	0.8241	0.8811	0.8375
300.0000	1.7597	1.9357	0.9004	0.8210	0.8764	0.8318
310.0000	1.7985	2.0134	0.8959	0.8160	0.8737	0.8285
320.0000	1.8606	2.0622	0.8961	0.8067	0.8684	0.8217
330.0000	1.8995	2.1307	0.8935	0.8086	0.8672	0.8191
340.0000	1.9521	2.1880	0.8892	0.7980	0.8632	0.8142
350.0000	1.9978	2.2495	0.8867	0.7949	0.8581	0.8082
360.0000	2.0485	2.2792	0.8816	0.7930	0.8548	0.8044
370.0000	2.0724	2.3463	0.8809	0.7912	0.8509	0.8003
380.0000	2.1280	2.4007	0.8784	0.7868	0.8476	0.7962
390.0000	2.1700	2.4360	0.8757	0.7800	0.8439	0.7920
400.0000	2.1977	2.4728	0.8749	0.7769	0.8452	0.7932
410.0000	2.2277	2.5131	0.8730	0.7763	0.8340	0.7812
420.0000	2.2592	2.5484	0.8699	0.7700	0.8331	0.7800
430.0000	2.2883	2.5901	0.8689	0.7645	0.8240	0.7703
440.0000	2.3207	2.6481	0.8676	0.7620	0.8192	0.7643
450.0000	2.3654	2.6862	0.8653	0.7564	0.8131	0.7579
460.0000	2.3945	2.7131	0.8637	0.7539	0.8096	0.7530

470.0000	2.4155	2.7435	0.8640	0.7526	0.8032	0.7459
480.0000	2.4378	2.7838	0.8658	0.7489	0.7982	0.7399
490.0000	2.4664	2.8014	0.8712	0.7446	0.7970	0.7384
500.0000	2.4781	2.8297	0.8749	0.7427	0.7912	0.7320
510.0000	2.4987	2.8438	0.8704	0.7396	0.7874	0.7275
520.0000	2.5079	2.8650	0.8641	0.7390	0.7814	0.7207
530.0000	2.5242	2.8785	0.8548	0.7352	0.7790	0.7181
540.0000	2.5344	2.9039	0.8678	0.7303	0.7755	0.7147
550.0000	2.5535	2.9145	0.8603	0.7259	0.7710	0.7091
560.0000	2.5616	2.9180	0.8609	0.7278	0.7665	0.7038
570.0000	2.5651	2.9385	0.8586	0.7259	0.7643	0.7020
580.0000	2.5790	2.9406	0.8523	0.7228	0.7609	0.6975
590.0000	2.5796	2.9633	0.8496	0.7209	0.7558	0.6918
600.0000	2.5957	2.9590	0.8455	0.7147	0.7517	0.6881
Cu^{2+}	$2*10^{-4}$ mol/L					
t (s)	RBH-MFI	RBH-R	Calcein-MFI	Calcein-G	MDAC-MFI	MDAC-B
0.0000	1.0000	1.0000	1.0000	1.0000	1.0000	1.0000
10.0000	1.0214	1.0245	0.9912	0.9877	0.9922	0.9938
20.0000	1.0413	1.0482	0.9811	0.9812	0.9764	0.9928
30.0000	1.0633	1.0743	0.9751	0.9740	0.9402	0.9887
40.0000	1.0873	1.1041	0.9749	0.9690	0.9414	0.9820
50.0000	1.1127	1.1355	0.9662	0.9661	0.9406	0.9820
60.0000	1.1384	1.1669	0.9602	0.9646	0.9433	0.9861
70.0000	1.1613	1.1968	0.9559	0.9610	0.9486	0.9830
80.0000	1.1871	1.2282	0.9557	0.9581	0.9449	0.9784
90.0000	1.2097	1.2573	0.9426	0.9574	0.9440	0.9815
100.0000	1.2349	1.2894	0.9377	0.9567	0.9398	0.9846
110.0000	1.2578	1.3201	0.9361	0.9531	0.9364	0.9799
120.0000	1.2828	1.3507	0.9352	0.9524	0.9271	0.9810
130.0000	1.3066	1.3836	0.9281	0.9480	0.9208	0.9794
140.0000	1.3276	1.4104	0.9273	0.9451	0.9181	0.9815
150.0000	1.3516	1.4403	0.9249	0.9408	0.9103	0.9871
160.0000	1.3762	1.4717	0.9237	0.9336	0.9081	0.9815
170.0000	1.3985	1.4992	0.9182	0.9271	0.9123	0.9784
180.0000	1.4221	1.5291	0.9138	0.9256	0.9132	0.9727
190.0000	1.4440	1.5551	0.9107	0.9264	0.9171	0.9676
200.0000	1.4635	1.5796	0.9051	0.9227	0.9118	0.9547
210.0000	1.4821	1.6034	0.9029	0.9213	0.9090	0.9460
220.0000	1.5076	1.6355	0.9023	0.9220	0.8988	0.9414
230.0000	1.5374	1.6731	0.9009	0.9235	0.8906	0.9311
240.0000	1.5539	1.6945	0.8986	0.9242	0.8904	0.9270
250.0000	1.5687	1.7221	0.8959	0.9256	0.8854	0.9316
260.0000	1.5919	1.7435	0.8942	0.9256	0.8891	0.9336
270.0000	1.6093	1.7649	0.8922	0.9148	0.8840	0.9383
280.0000	1.6266	1.7871	0.8870	0.9098	0.8719	0.9311
290.0000	1.6447	1.8070	0.8891	0.9119	0.8668	0.9280
300.0000	1.6622	1.8300	0.8835	0.9098	0.8645	0.9156
310.0000	1.6799	1.8476	0.8833	0.9119	0.8694	0.9018
320.0000	1.6945	1.8783	0.8820	0.9177	0.8604	0.9023
330.0000	1.7196	1.8943	0.8857	0.9061	0.8573	0.8971
340.0000	1.7334	1.9112	0.8830	0.8931	0.8518	0.9033
350.0000	1.7468	1.9334	0.8796	0.8809	0.8485	0.8997
360.0000	1.7647	1.9472	0.8786	0.8960	0.8348	0.8935
370.0000	1.7758	1.9640	0.8779	0.8852	0.8298	0.8740
380.0000	1.7904	1.9778	0.8751	0.8859	0.8269	0.8652
390.0000	1.8025	1.9962	0.8726	0.8816	0.8226	0.8647
400.0000	1.8176	2.0054	0.8715	0.8715	0.8250	0.8740
410.0000	1.8263	2.0314	0.8695	0.8376	0.8254	0.8601

420.0000	1.8475	2.0536	0.8687	0.8253	0.8224	0.8524
430.0000	1.8657	2.0750	0.8679	0.8173	0.8161	0.8452
440.0000	1.8846	2.0927	0.8662	0.8224	0.8065	0.8426
450.0000	1.9000	2.1018	0.8639	0.8173	0.8018	0.8411
460.0000	1.9063	2.1294	0.8639	0.8029	0.7978	0.8231
470.0000	1.9272	2.1577	0.8623	0.8014	0.7934	0.8158
480.0000	1.9442	2.1876	0.8625	0.7986	0.7928	0.8128
490.0000	1.9579	2.2190	0.8601	0.7964	0.7963	0.8086
500.0000	1.9710	2.2580	0.8574	0.7935	0.7924	0.8117
510.0000	1.9886	2.3017	0.8557	0.7957	0.7842	0.8133
520.0000	2.0096	2.3591	0.8570	0.7913	0.7799	0.8102
530.0000	2.0412	2.4066	0.8561	0.7935	0.7767	0.8030
540.0000	2.0716	2.4610	0.8548	0.7928	0.7668	0.7891
550.0000	2.1108	2.5161	0.8539	0.7906	0.7706	0.7824
560.0000	2.1533	2.5704	0.8511	0.7870	0.7688	0.7788
570.0000	2.1995	2.6172	0.8475	0.7856	0.7684	0.7742
580.0000	2.2414	2.6731	0.8492	0.7863	0.7659	0.7742
590.0000	2.2910	2.7152	0.8464	0.7885	0.7637	0.7793
600.0000	2.3284	2.7389	0.8457	0.7882	0.7594	0.7747

Sheet 2

Al+Cu t (s)	RBH-MFI	RBH-R	Calcein-MFI	Calcein-G	MDAC-MFI	MDAC-B	TYPE
0.0000	1.0000	1.0000	1.0000	1.0000	1.0000	1.0000	1.0000
10.0000	1.0033	1.0011	1.0064	1.0011	0.9247	0.9400	1.0000
20.0000	1.0096	1.0066	1.0144	1.0017	0.8624	0.8980	1.0000
30.0000	1.0178	1.0121	1.0203	1.0015	0.8166	0.8658	1.0000
40.0000	1.0222	1.0166	1.0261	1.0015	0.7353	0.8022	1.0000
50.0000	1.0301	1.0210	1.0316	1.0017	0.7034	0.7753	1.0000
60.0000	1.0376	1.0265	1.0379	1.0008	0.6839	0.7589	1.0000
70.0000	1.0417	1.0298	1.0383	1.0000	0.6711	0.7449	1.0000
80.0000	1.0491	1.0364	1.0370	1.0001	0.6610	0.7317	1.0000
90.0000	1.0577	1.0430	1.0360	0.9992	0.6477	0.7144	1.0000
100.0000	1.0691	1.0541	1.0310	0.9985	0.6335	0.6990	1.0000
110.0000	1.0744	1.0596	1.0258	0.9994	0.6202	0.6847	1.0000
120.0000	1.0841	1.0673	1.0252	0.9988	0.6106	0.6747	1.0000
130.0000	1.0964	1.0795	1.0172	0.9985	0.5975	0.6605	1.0000
140.0000	1.1038	1.0861	1.0118	0.9981	0.5902	0.6531	1.0000
150.0000	1.1130	1.0960	1.0078	0.9945	0.5775	0.6393	1.0000
160.0000	1.1228	1.1049	0.9987	0.9902	0.5687	0.6315	1.0000
170.0000	1.1287	1.1093	0.9893	0.9863	0.5616	0.6228	1.0000
180.0000	1.1366	1.1170	0.9781	0.9874	0.5542	0.6140	1.0000
190.0000	1.1459	1.1258	0.9823	0.9866	0.5430	0.6011	1.0000
200.0000	1.1565	1.1358	0.9810	0.9850	0.5383	0.5962	1.0000
210.0000	1.1651	1.1457	0.9741	0.9828	0.5308	0.5867	1.0000
220.0000	1.1709	1.1501	0.9649	0.9859	0.5202	0.5742	1.0000
230.0000	1.1809	1.1578	0.9609	0.9964	0.5151	0.5686	1.0000
240.0000	1.1893	1.1656	0.9576	1.0062	0.5079	0.5605	1.0000
250.0000	1.1994	1.1744	0.9548	1.0170	0.5004	0.5521	1.0000
260.0000	1.2080	1.1821	0.9529	1.0302	0.4954	0.5474	1.0000
270.0000	1.2125	1.1909	0.9546	1.0413	0.4894	0.5420	1.0000
280.0000	1.2262	1.1965	0.9536	1.0518	0.4828	0.5345	1.0000
290.0000	1.2346	1.2042	0.9554	1.0594	0.4762	0.5280	1.0000
300.0000	1.2426	1.2108	0.9632	1.0632	0.4713	0.5234	1.0000
310.0000	1.2534	1.2196	0.9694	1.0644	0.4665	0.5179	1.0000
320.0000	1.2646	1.2285	0.9742	1.0626	0.4543	0.5043	1.0000
330.0000	1.2774	1.2395	0.9723	1.0644	0.4494	0.4991	1.0000
340.0000	1.2880	1.2483	0.9762	1.0645	0.4466	0.4951	1.0000
350.0000	1.3014	1.2605	0.9771	1.0645	0.4422	0.4907	1.0000
360.0000	1.3085	1.2660	0.9790	1.0632	0.4383	0.4854	1.0000
370.0000	1.3264	1.2826	0.9777	1.0633	0.4300	0.4761	1.0000
380.0000	1.3363	1.2925	0.9780	1.0632	0.4270	0.4726	1.0000
390.0000	1.3442	1.3002	0.9779	1.0614	0.4255	0.4707	1.0000
400.0000	1.3591	1.3146	0.9731	1.0619	0.4212	0.4661	1.0000
410.0000	1.3680	1.3223	0.9738	1.0616	0.4195	0.4638	1.0000
420.0000	1.3766	1.3311	0.9723	1.0591	0.4179	0.4621	1.0000
430.0000	1.3843	1.3389	0.9670	1.0598	0.4103	0.4529	1.0000
440.0000	1.3881	1.3444	0.9675	1.0612	0.4063	0.4494	1.0000
450.0000	1.3990	1.3554	0.9698	1.0597	0.4014	0.4436	1.0000
460.0000	1.4064	1.3642	0.9667	1.0590	0.3963	0.4383	1.0000
470.0000	1.4143	1.3720	0.9644	1.0594	0.3934	0.4349	1.0000
480.0000	1.4212	1.3786	0.9644	1.0612	0.3885	0.4298	1.0000
490.0000	1.4280	1.3863	0.9666	1.0580	0.3839	0.4247	1.0000
500.0000	1.4330	1.3918	0.9608	1.0598	0.3782	0.4181	1.0000
510.0000	1.4380	1.3985	0.9626	1.0616	0.3748	0.4149	1.0000
520.0000	1.4376	1.3985	0.9656	1.0619	0.3704	0.4099	1.0000
530.0000	1.4511	1.4117	0.9645	1.0618	0.3717	0.4132	1.0000
540.0000	1.4503	1.4117	0.9638	1.0604	0.3656	0.4053	1.0000

550.0000	1.4623	1.4238	0.9603	1.0615	0.3615	0.4006	1.0000
560.0000	1.4679	1.4316	0.9609	1.0627	0.3557	0.3939	1.0000
570.0000	1.4719	1.4349	0.9621	1.0622	0.3531	0.3908	1.0000
580.0000	1.4764	1.4415	0.9601	1.0622	0.3487	0.3855	1.0000
590.0000	1.4817	1.4459	0.9598	1.0618	0.3441	0.3802	1.0000
600.0000	1.4847	1.4503	0.9580	1.0627	0.3399	0.3755	1.0000
0.0000	1.0000	1.0000	1.0000	1.0000	1.0000	1.0000	2.0000
10.0000	1.0050	1.0074	1.0011	1.0015	0.9417	0.9579	2.0000
20.0000	1.0102	1.0137	0.9982	1.0072	0.8985	0.9224	2.0000
30.0000	1.0161	1.0222	0.9978	1.0168	0.8575	0.8854	2.0000
40.0000	1.0225	1.0306	0.9994	1.0231	0.8288	0.8576	2.0000
50.0000	1.0284	1.0380	1.0018	1.0223	0.8020	0.8311	2.0000
60.0000	1.0366	1.0496	1.0014	1.0069	0.7760	0.8047	2.0000
70.0000	1.0419	1.0559	1.0024	0.9901	0.7522	0.7812	2.0000
80.0000	1.0484	1.0665	1.0025	0.9745	0.7286	0.7582	2.0000
90.0000	1.0544	1.0759	1.0009	0.9655	0.7031	0.7329	2.0000
100.0000	1.0590	1.0844	0.9973	0.9594	0.6837	0.7138	2.0000
110.0000	1.0650	1.0949	0.9901	0.9552	0.6658	0.6961	2.0000
120.0000	1.0732	1.1086	0.9797	0.9507	0.6485	0.6793	2.0000
130.0000	1.0816	1.1203	0.9696	0.9474	0.6343	0.6650	2.0000
140.0000	1.0914	1.1350	0.9586	0.9423	0.6196	0.6505	2.0000
150.0000	1.1008	1.1487	0.9530	0.9361	0.6074	0.6386	2.0000
160.0000	1.1116	1.1646	0.9478	0.9308	0.5961	0.6274	2.0000
170.0000	1.1218	1.1783	0.9388	0.9212	0.5845	0.6154	2.0000
180.0000	1.1338	1.1951	0.9293	0.9107	0.5716	0.6027	2.0000
190.0000	1.1473	1.2141	0.9233	0.9024	0.5648	0.5963	2.0000
200.0000	1.1589	1.2310	0.9230	0.8976	0.5548	0.5863	2.0000
210.0000	1.1692	1.2447	0.9203	0.8889	0.5454	0.5762	2.0000
220.0000	1.1827	1.2627	0.9185	0.8822	0.5369	0.5675	2.0000
230.0000	1.1972	1.2827	0.9166	0.8776	0.5294	0.5592	2.0000
240.0000	1.2114	1.3027	0.9143	0.8736	0.5218	0.5509	2.0000
250.0000	1.2251	1.3217	0.9130	0.8711	0.5137	0.5425	2.0000
260.0000	1.2419	1.3449	0.9121	0.8694	0.5061	0.5345	2.0000
270.0000	1.2563	1.3650	0.9116	0.8683	0.4992	0.5274	2.0000
280.0000	1.2716	1.3871	0.9104	0.8661	0.4925	0.5212	2.0000
290.0000	1.2890	1.4103	0.9102	0.8654	0.4826	0.5106	2.0000
300.0000	1.3053	1.4325	0.9096	0.8649	0.4761	0.5031	2.0000
310.0000	1.3242	1.4599	0.9083	0.8631	0.4672	0.4932	2.0000
320.0000	1.3423	1.4852	0.9087	0.8637	0.4584	0.4838	2.0000
330.0000	1.3596	1.5105	0.9074	0.8614	0.4522	0.4778	2.0000
340.0000	1.3796	1.5390	0.9072	0.8614	0.4432	0.4691	2.0000
350.0000	1.4005	1.5675	0.9063	0.8599	0.4357	0.4618	2.0000
360.0000	1.4223	1.5981	0.9055	0.8590	0.4277	0.4537	2.0000
370.0000	1.4435	1.6266	0.9044	0.8575	0.4214	0.4479	2.0000
380.0000	1.4677	1.6614	0.9039	0.8570	0.4139	0.4408	2.0000
390.0000	1.4950	1.6994	0.9032	0.8580	0.4071	0.4340	2.0000
400.0000	1.5239	1.7384	0.9029	0.8441	0.4007	0.4271	2.0000
410.0000	1.5651	1.7954	0.9027	0.8441	0.3945	0.4211	2.0000
420.0000	1.6087	1.8555	0.9014	0.8426	0.3892	0.4157	2.0000
430.0000	1.6660	1.9357	0.9010	0.8423	0.3834	0.4101	2.0000
440.0000	1.7291	2.0222	0.9007	0.8419	0.3791	0.4058	2.0000
450.0000	1.7995	2.1234	0.9002	0.8413	0.3748	0.4016	2.0000
460.0000	1.8830	2.1835	0.8999	0.8413	0.3709	0.3979	2.0000
470.0000	1.9688	2.2859	0.8988	0.8399	0.3665	0.3937	2.0000
480.0000	2.0526	2.3935	0.8983	0.8394	0.3624	0.3896	2.0000
490.0000	2.1329	2.4947	0.8981	0.8396	0.3591	0.3865	2.0000
500.0000	2.2116	2.5918	0.8971	0.8381	0.3563	0.3838	2.0000
510.0000	2.2939	2.6962	0.8960	0.8369	0.3530	0.3802	2.0000

520.0000	2.3622	2.7816	0.8964	0.8374	0.3496	0.3771	2.0000
530.0000	2.4307	2.8671	0.8955	0.8367	0.3474	0.3748	2.0000
540.0000	2.5088	2.9568	0.8948	0.8359	0.3449	0.3724	2.0000
550.0000	2.5702	3.0295	0.8949	0.8361	0.3424	0.3699	2.0000
560.0000	2.6425	3.1234	0.8942	0.8354	0.3409	0.3686	2.0000
570.0000	2.7070	3.2036	0.8938	0.8351	0.3383	0.3657	2.0000
580.0000	2.7634	3.2764	0.8939	0.8356	0.3359	0.3632	2.0000
590.0000	2.8136	3.3365	0.8935	0.8349	0.3345	0.3618	2.0000
600.0000	2.7862	3.3270	0.8926	0.8339	0.3332	0.3605	2.0000
0.0000	1.0000	1.0000	1.0000	1.0000	1.0000	1.0000	3.0000
10.0000	1.0092	1.0093	1.0011	0.9876	0.9514	0.9420	3.0000
20.0000	1.0187	1.0175	1.0061	0.9756	0.9122	0.8974	3.0000
30.0000	1.0295	1.0268	1.0072	0.9667	0.8837	0.8537	3.0000
40.0000	1.0411	1.0373	1.0087	0.9594	0.8378	0.8142	3.0000
50.0000	1.0535	1.0501	1.0138	0.9512	0.8129	0.7826	3.0000
60.0000	1.0672	1.0640	1.0103	0.9414	0.7855	0.7559	3.0000
70.0000	1.0825	1.0803	1.0095	0.9305	0.7595	0.7313	3.0000
80.0000	1.1001	1.0978	1.0042	0.9241	0.7371	0.7110	3.0000
90.0000	1.1172	1.1153	1.0029	0.9210	0.7154	0.6903	3.0000
100.0000	1.1347	1.1339	0.9992	0.9177	0.6989	0.6749	3.0000
110.0000	1.1547	1.1537	0.9973	0.9157	0.6845	0.6603	3.0000
120.0000	1.1752	1.1735	0.9966	0.9141	0.6718	0.6478	3.0000
130.0000	1.1965	1.1944	0.9946	0.9123	0.6584	0.6351	3.0000
140.0000	1.2189	1.2165	0.9907	0.9103	0.6471	0.6239	3.0000
150.0000	1.2432	1.2410	0.9883	0.9090	0.6374	0.6141	3.0000
160.0000	1.2662	1.2631	0.9839	0.9070	0.6267	0.6041	3.0000
170.0000	1.2912	1.2887	0.9817	0.9057	0.6174	0.5944	3.0000
180.0000	1.3156	1.3132	0.9794	0.9044	0.6075	0.5847	3.0000
190.0000	1.3409	1.3388	0.9754	0.9026	0.5993	0.5765	3.0000
200.0000	1.3654	1.3632	0.9719	0.9012	0.5911	0.5686	3.0000
210.0000	1.3919	1.3877	0.9685	0.8993	0.5832	0.5607	3.0000
220.0000	1.4197	1.4133	0.9642	0.8966	0.5754	0.5531	3.0000
230.0000	1.4465	1.4389	0.9594	0.8932	0.5694	0.5471	3.0000
240.0000	1.4711	1.4645	0.9548	0.8895	0.5620	0.5398	3.0000
250.0000	1.4975	1.4901	0.9494	0.8851	0.5568	0.5346	3.0000
260.0000	1.5229	1.5157	0.9466	0.8824	0.5495	0.5273	3.0000
270.0000	1.5476	1.5402	0.9436	0.8797	0.5430	0.5209	3.0000
280.0000	1.5695	1.5634	0.9436	0.8766	0.5375	0.5155	3.0000
290.0000	1.5938	1.5891	0.9396	0.8750	0.5322	0.5100	3.0000
300.0000	1.6169	1.6112	0.9379	0.8717	0.5259	0.5036	3.0000
310.0000	1.6413	1.6368	0.9330	0.8708	0.5215	0.4994	3.0000
320.0000	1.6641	1.6601	0.9310	0.8700	0.5169	0.4942	3.0000
330.0000	1.6868	1.6834	0.9290	0.8693	0.5129	0.4903	3.0000
340.0000	1.7096	1.7055	0.9270	0.8700	0.5082	0.4854	3.0000
350.0000	1.7347	1.7299	0.9258	0.8730	0.5036	0.4806	3.0000
360.0000	1.7580	1.7532	0.9246	0.8753	0.4993	0.4763	3.0000
370.0000	1.7809	1.7765	0.9225	0.8799	0.4954	0.4724	3.0000
380.0000	1.8049	1.8009	0.9212	0.8859	0.4924	0.4687	3.0000
390.0000	1.8258	1.8242	0.9205	0.8908	0.4884	0.4651	3.0000
400.0000	1.8516	1.8510	0.9193	0.8961	0.4851	0.4614	3.0000
410.0000	1.8741	1.8766	0.9191	0.9012	0.4807	0.4569	3.0000
420.0000	1.8972	1.9010	0.9190	0.9052	0.4789	0.4548	3.0000
430.0000	1.9208	1.9267	0.9186	0.9090	0.4772	0.4532	3.0000
440.0000	1.9410	1.9523	0.9180	0.9128	0.4763	0.4520	3.0000
450.0000	1.9661	1.9849	0.9175	0.9166	0.4736	0.4496	3.0000
460.0000	1.9908	2.0116	0.9177	0.9197	0.4712	0.4466	3.0000
470.0000	2.0142	2.0454	0.9176	0.9221	0.4689	0.4444	3.0000
480.0000	2.0343	2.0768	0.9172	0.9256	0.4675	0.4432	3.0000

490.0000	2.0596	2.1164	0.9178	0.9279	0.4654	0.4411	3.0000
500.0000	2.0768	2.1455	0.9181	0.9297	0.4647	0.4399	3.0000
510.0000	2.1019	2.1839	0.9184	0.9307	0.4624	0.4378	3.0000
520.0000	2.1260	2.2200	0.9187	0.9308	0.4623	0.4372	3.0000
530.0000	2.1512	2.2549	0.9185	0.9321	0.4608	0.4344	3.0000
540.0000	2.1725	2.2829	0.9196	0.9297	0.4597	0.4329	3.0000
550.0000	2.1976	2.3132	0.9188	0.9299	0.4588	0.4317	3.0000
560.0000	2.2166	2.3364	0.9196	0.9283	0.4562	0.4296	3.0000
570.0000	2.2401	2.3644	0.9194	0.9276	0.4526	0.4262	3.0000
580.0000	2.2590	2.3865	0.9198	0.9257	0.4493	0.4223	3.0000
590.0000	2.2779	2.4098	0.9199	0.9241	0.4473	0.4208	3.0000
600.0000	2.2997	2.4354	0.9202	0.9221	0.4443	0.4180	3.0000
0.0000	1.0000	1.0000	1.0000	1.0000	1.0000	1.0000	4.0000
10.0000	1.0001	1.0000	1.0016	0.9982	0.9460	0.9239	4.0000
20.0000	1.0007	1.0000	1.0036	0.9948	0.9069	0.8557	4.0000
30.0000	1.0007	1.0012	0.9841	0.9849	0.8641	0.8038	4.0000
40.0000	1.0002	1.0024	0.9481	0.9686	0.8291	0.7645	4.0000
50.0000	1.0002	1.0036	0.9070	0.9496	0.7994	0.7318	4.0000
60.0000	1.0003	1.0059	0.8814	0.9353	0.7722	0.7047	4.0000
70.0000	0.9999	1.0071	0.8767	0.9299	0.7462	0.6820	4.0000
80.0000	0.9998	1.0095	0.8752	0.9256	0.7273	0.6638	4.0000
90.0000	0.9998	1.0118	0.8737	0.9228	0.7081	0.6474	4.0000
100.0000	0.9995	1.0142	0.8689	0.9192	0.6896	0.6319	4.0000
110.0000	1.0151	1.0166	0.8659	0.9172	0.6737	0.6189	4.0000
120.0000	1.0167	1.0201	0.8692	0.9158	0.6571	0.6048	4.0000
130.0000	1.0186	1.0237	0.8665	0.9172	0.6405	0.5908	4.0000
140.0000	1.0206	1.0273	0.8657	0.9126	0.6232	0.5761	4.0000
150.0000	1.0225	1.0320	0.8504	0.9033	0.6075	0.5633	4.0000
160.0000	1.0264	1.0355	0.8411	0.8934	0.5888	0.5471	4.0000
170.0000	1.0287	1.0391	0.8407	0.8884	0.5742	0.5349	4.0000
180.0000	1.0311	1.0438	0.8384	0.8898	0.5621	0.5248	4.0000
190.0000	1.0347	1.0474	0.8297	0.8918	0.5494	0.5139	4.0000
200.0000	1.0380	1.0521	0.8296	0.8952	0.5358	0.5027	4.0000
210.0000	1.0414	1.0581	0.8353	0.9003	0.5245	0.4930	4.0000
220.0000	1.0456	1.0628	0.8446	0.9039	0.5134	0.4837	4.0000
230.0000	1.0497	1.0675	0.8471	0.9037	0.5040	0.4750	4.0000
240.0000	1.0532	1.0723	0.8406	0.9037	0.4938	0.4657	4.0000
250.0000	1.0552	1.0746	0.8379	0.9055	0.4835	0.4560	4.0000
260.0000	1.0565	1.0782	0.8375	0.9063	0.4744	0.4475	4.0000
270.0000	1.0595	1.0818	0.8368	0.9061	0.4658	0.4398	4.0000
280.0000	1.0616	1.0889	0.8358	0.9031	0.4551	0.4270	4.0000
290.0000	1.0665	1.0936	0.8316	0.8986	0.4470	0.4218	4.0000
300.0000	1.0698	1.0995	0.8283	0.8928	0.4392	0.4144	4.0000
310.0000	1.0891	1.1031	0.8232	0.8856	0.4306	0.4055	4.0000
320.0000	1.0941	1.1090	0.8150	0.8819	0.4232	0.3989	4.0000
330.0000	1.0978	1.1137	0.8126	0.8779	0.4155	0.3916	4.0000
340.0000	1.1032	1.1197	0.8085	0.8751	0.4077	0.3842	4.0000
350.0000	1.1077	1.1232	0.8052	0.8727	0.4005	0.3786	4.0000
360.0000	1.1118	1.1303	0.8025	0.8704	0.3926	0.3710	4.0000
370.0000	1.1168	1.1410	0.7996	0.8690	0.3846	0.3639	4.0000
380.0000	1.1230	1.1600	0.7977	0.8682	0.3782	0.3581	4.0000
390.0000	1.1326	1.1919	0.7965	0.8666	0.3712	0.3522	4.0000
400.0000	1.1520	1.2393	0.7944	0.8654	0.3651	0.3468	4.0000
410.0000	1.1854	1.2927	0.7920	0.8652	0.3608	0.3433	4.0000
420.0000	1.2336	1.3448	0.7909	0.8656	0.3553	0.3387	4.0000
430.0000	1.2867	1.3969	0.7908	0.8656	0.3521	0.3360	4.0000
440.0000	1.3376	1.4419	0.7901	0.8654	0.3480	0.3325	4.0000
450.0000	1.3853	1.4870	0.7893	0.8660	0.3451	0.3302	4.0000

460.0000	1.4278	1.5320	0.7886	0.8662	0.3425	0.3282	4.0000
470.0000	1.4704	1.5735	0.7879	0.8670	0.3400	0.3257	4.0000
480.0000	1.5125	1.6137	0.7873	0.8672	0.3379	0.3240	4.0000
490.0000	1.5510	1.6552	0.7886	0.8690	0.3358	0.3222	4.0000
500.0000	1.5875	1.6908	0.7883	0.8692	0.3337	0.3203	4.0000
510.0000	1.6261	1.7275	0.7885	0.8702	0.3321	0.3189	4.0000
520.0000	1.6587	1.7630	0.7884	0.8706	0.3308	0.3178	4.0000
530.0000	1.6913	1.7974	0.7878	0.8706	0.3289	0.3162	4.0000
540.0000	1.7232	1.8341	0.7879	0.8716	0.3277	0.3151	4.0000
550.0000	1.7541	1.8709	0.7877	0.8721	0.3264	0.3139	4.0000
560.0000	1.7856	1.9040	0.7890	0.8725	0.3247	0.3124	4.0000
570.0000	1.8168	1.9348	0.7895	0.8739	0.3233	0.3112	4.0000
580.0000	1.8453	1.9704	0.7899	0.8749	0.3224	0.3104	4.0000
590.0000	1.8740	2.0012	0.7914	0.8761	0.3207	0.3091	4.0000
600.0000	1.9044	2.0284	0.7918	0.8769	0.3193	0.3077	4.0000
0.0000	1.0000	1.0000	1.0000	1.0000	1.0000	1.0000	5.0000
10.0000	1.0019	1.0012	1.0028	0.9891	0.9110	0.9081	5.0000
20.0000	1.0015	1.0012	1.0045	0.9847	0.8393	0.8388	5.0000
30.0000	1.0032	1.0023	1.0058	0.9788	0.7844	0.7856	5.0000
40.0000	1.0011	1.0046	1.0055	0.9746	0.7336	0.7364	5.0000
50.0000	1.0025	1.0058	1.0032	0.9712	0.6924	0.6957	5.0000
60.0000	1.0017	1.0081	1.0017	0.9692	0.6602	0.6641	5.0000
70.0000	1.0028	1.0092	1.0009	0.9659	0.6345	0.6396	5.0000
80.0000	1.0054	1.0127	1.0024	0.9639	0.6115	0.6171	5.0000
90.0000	1.0053	1.0161	1.0034	0.9619	0.5934	0.5990	5.0000
100.0000	1.0054	1.0173	1.0045	0.9601	0.5753	0.5816	5.0000
110.0000	1.0032	1.0196	1.0047	0.9581	0.5591	0.5661	5.0000
120.0000	0.9988	1.0219	1.0044	0.9563	0.5441	0.5514	5.0000
130.0000	1.0090	1.0219	0.9992	0.9514	0.5275	0.5355	5.0000
140.0000	0.9999	1.0253	0.9979	0.9492	0.5124	0.5211	5.0000
150.0000	1.0144	1.0253	0.9949	0.9468	0.4985	0.5069	5.0000
160.0000	1.0129	1.0300	0.9978	0.9470	0.4871	0.4966	5.0000
170.0000	1.0143	1.0311	0.9974	0.9460	0.4745	0.4833	5.0000
180.0000	1.0186	1.0357	0.9958	0.9435	0.4622	0.4708	5.0000
190.0000	1.0298	1.0415	0.9918	0.9401	0.4499	0.4581	5.0000
200.0000	1.0391	1.0472	0.9949	0.9405	0.4410	0.4488	5.0000
210.0000	1.0512	1.0576	0.9946	0.9391	0.4295	0.4373	5.0000
220.0000	1.0701	1.0714	0.9872	0.9333	0.4208	0.4280	5.0000
230.0000	1.0949	1.0910	0.9889	0.9339	0.4115	0.4179	5.0000
240.0000	1.1278	1.1187	0.9876	0.9313	0.4029	0.4084	5.0000
250.0000	1.1703	1.1532	0.9850	0.9278	0.3944	0.3996	5.0000
260.0000	1.2139	1.1970	0.9797	0.9220	0.3839	0.3885	5.0000
270.0000	1.2584	1.2431	0.9788	0.9188	0.3768	0.3814	5.0000
280.0000	1.3053	1.2903	0.9799	0.9159	0.3691	0.3738	5.0000
290.0000	1.3543	1.3410	0.9704	0.9065	0.3630	0.3677	5.0000
300.0000	1.3768	1.3952	0.9690	0.9034	0.3581	0.3628	5.0000
310.0000	1.4762	1.4470	0.9679	0.9054	0.3532	0.3579	5.0000
320.0000	1.5256	1.5023	0.9651	0.9030	0.3489	0.3537	5.0000
330.0000	1.5691	1.5588	0.9605	0.9006	0.3456	0.3503	5.0000
340.0000	1.6032	1.6071	0.9616	0.9095	0.3425	0.3476	5.0000
350.0000	1.6472	1.6452	0.9603	0.9202	0.3393	0.3449	5.0000
360.0000	1.6849	1.6970	0.9602	0.9254	0.3364	0.3420	5.0000
370.0000	1.7261	1.7431	0.9589	0.9198	0.3334	0.3388	5.0000
380.0000	1.7650	1.7915	0.9558	0.9097	0.3318	0.3368	5.0000
390.0000	1.7916	1.8376	0.9527	0.8982	0.3294	0.3341	5.0000
400.0000	1.8255	1.8710	0.9493	0.8927	0.3268	0.3315	5.0000
410.0000	1.9051	1.9147	0.9447	0.8875	0.3233	0.3280	5.0000
420.0000	1.9280	1.9597	0.9399	0.8833	0.3199	0.3244	5.0000

430.0000	1.9280	1.9885	0.9391	0.8849	0.3178	0.3217	5.0000
440.0000	1.9639	2.0334	0.9349	0.8813	0.3177	0.3207	5.0000
450.0000	1.9890	2.0645	0.9337	0.8823	0.3182	0.3197	5.0000
460.0000	2.0153	2.0968	0.9318	0.8825	0.3168	0.3182	5.0000
470.0000	2.0373	2.1233	0.9279	0.8806	0.3152	0.3168	5.0000
480.0000	2.0571	2.1475	0.9242	0.8794	0.3140	0.3153	5.0000
490.0000	2.0853	2.1820	0.9186	0.8750	0.3124	0.3138	5.0000
500.0000	2.1077	2.2097	0.9160	0.8732	0.3094	0.3109	5.0000
510.0000	2.1193	2.2281	0.9140	0.8720	0.3081	0.3097	5.0000
520.0000	2.1382	2.2535	0.9101	0.8679	0.3052	0.3072	5.0000
530.0000	2.1587	2.2800	0.9092	0.8685	0.3034	0.3055	5.0000
540.0000	2.1886	2.3157	0.9098	0.8708	0.3014	0.3038	5.0000
550.0000	2.2108	2.3422	0.9101	0.8720	0.3009	0.3035	5.0000
560.0000	2.2289	2.3641	0.9080	0.8700	0.2992	0.3025	5.0000
570.0000	2.2491	2.3894	0.9081	0.8710	0.2974	0.3013	5.0000
580.0000	2.2620	2.4067	0.9075	0.8706	0.2958	0.3006	5.0000
590.0000	2.2691	2.4147	0.9073	0.8704	0.2954	0.3003	5.0000
600.0000	2.2855	2.4355	0.9065	0.8685	0.2946	0.2994	5.0000
0.0000	1.0000	1.0000	1.0000	1.0000	1.0000	1.0000	6.0000
10.0000	0.9976	1.0049	1.0013	0.9893	0.9159	0.9276	6.0000
20.0000	0.9980	1.0074	0.9997	0.9887	0.8491	0.8711	6.0000
30.0000	0.9956	1.0110	1.0018	0.9891	0.7888	0.8180	6.0000
40.0000	0.9948	1.0135	1.0043	0.9907	0.7376	0.7716	6.0000
50.0000	1.0293	1.0172	1.0010	0.9907	0.6854	0.7242	6.0000
60.0000	1.0328	1.0208	1.0033	0.9911	0.6462	0.6826	6.0000
70.0000	1.0347	1.0257	1.0108	0.9931	0.6091	0.6453	6.0000
80.0000	1.0354	1.0319	1.0139	0.9947	0.5785	0.6152	6.0000
90.0000	1.0379	1.0368	1.0050	0.9978	0.5486	0.5898	6.0000
100.0000	1.0432	1.0453	1.0108	1.0022	0.5230	0.5671	6.0000
110.0000	1.0476	1.0515	1.0076	1.0102	0.5034	0.5447	6.0000
120.0000	1.0553	1.0600	1.0018	1.0220	0.4827	0.5259	6.0000
130.0000	1.0621	1.0686	0.9949	1.0310	0.4649	0.5099	6.0000
140.0000	1.0718	1.0797	0.9856	1.0384	0.4475	0.4932	6.0000
150.0000	1.0818	1.0895	0.9712	1.0468	0.4330	0.4771	6.0000
160.0000	1.0909	1.0993	0.9854	1.0532	0.4204	0.4635	6.0000
170.0000	1.0998	1.1078	0.9815	1.0586	0.4114	0.4537	6.0000
180.0000	1.1110	1.1189	0.9705	1.0674	0.3990	0.4403	6.0000
190.0000	1.1205	1.1275	0.9723	1.0789	0.3910	0.4321	6.0000
200.0000	1.1419	1.1385	0.9692	1.0901	0.3821	0.4232	6.0000
210.0000	1.1551	1.1471	0.9639	1.0993	0.3729	0.4134	6.0000
220.0000	1.1683	1.1593	0.9599	1.1051	0.3654	0.4059	6.0000
230.0000	1.1854	1.1703	0.9495	1.1067	0.3550	0.3950	6.0000
240.0000	1.1948	1.1863	0.9482	1.1085	0.3484	0.3883	6.0000
250.0000	1.2115	1.1949	0.9441	1.1082	0.3426	0.3820	6.0000
260.0000	1.2209	1.2120	0.9443	1.1109	0.3366	0.3757	6.0000
270.0000	1.2354	1.2194	0.9438	1.1133	0.3303	0.3686	6.0000
280.0000	1.2469	1.2328	0.9379	1.1127	0.3250	0.3627	6.0000
290.0000	1.2554	1.2426	0.9381	1.1166	0.3193	0.3569	6.0000
300.0000	1.2614	1.2512	0.9366	1.1195	0.3141	0.3510	6.0000
310.0000	1.2646	1.2549	0.9320	1.1195	0.3094	0.3463	6.0000
320.0000	1.2733	1.2574	0.9296	1.1202	0.3057	0.3421	6.0000
330.0000	1.2750	1.2659	0.9300	1.1235	0.2998	0.3361	6.0000
340.0000	1.2815	1.2672	0.9276	1.1280	0.2954	0.3317	6.0000
350.0000	1.2857	1.2721	0.9249	1.1249	0.2925	0.3287	6.0000
360.0000	1.2914	1.2757	0.9194	1.1197	0.2882	0.3244	6.0000
370.0000	1.3041	1.2806	0.9148	1.1153	0.2851	0.3213	6.0000
380.0000	1.3140	1.2929	0.9133	1.1151	0.2823	0.3185	6.0000
390.0000	1.3315	1.3027	0.9105	1.1135	0.2790	0.3153	6.0000

400.0000	1.3563	1.3174	0.9062	1.1102	0.2762	0.3122	6.0000
410.0000	1.3927	1.3407	0.9038	1.1082	0.2731	0.3092	6.0000
420.0000	1.4337	1.3713	0.9007	1.1054	0.2713	0.3077	6.0000
430.0000	1.4741	1.4081	0.8984	1.1044	0.2697	0.3060	6.0000
440.0000	1.5249	1.4424	0.8948	1.1003	0.2663	0.3027	6.0000
450.0000	1.5747	1.4865	0.8918	1.0974	0.2636	0.2999	6.0000
460.0000	1.6215	1.5294	0.8916	1.0983	0.2615	0.2977	6.0000
470.0000	1.6719	1.5686	0.8904	1.0982	0.2595	0.2958	6.0000
480.0000	1.7200	1.6152	0.8900	1.0989	0.2573	0.2936	6.0000
490.0000	1.7591	1.6593	0.8893	1.0989	0.2561	0.2925	6.0000
500.0000	1.8043	1.6949	0.8899	1.1009	0.2543	0.2906	6.0000
510.0000	1.8467	1.7390	0.8894	1.1013	0.2531	0.2895	6.0000
520.0000	1.8827	1.7806	0.8894	1.1025	0.2509	0.2871	6.0000
530.0000	1.9199	1.8223	0.8872	1.1002	0.2498	0.2862	6.0000
540.0000	1.9579	1.8591	0.8856	1.0991	0.2481	0.2845	6.0000
550.0000	2.0029	1.8995	0.8856	1.0998	0.2477	0.2841	6.0000
560.0000	2.0383	1.9473	0.8847	1.0996	0.2458	0.2819	6.0000
570.0000	2.0657	1.9828	0.8830	1.0983	0.2447	0.2813	6.0000
580.0000	2.1052	2.0098	0.8826	1.0987	0.2434	0.2800	6.0000
590.0000	2.1353	2.0502	0.8807	1.0969	0.2418	0.2784	6.0000
600.0000	2.1355	2.0931	0.8811	1.0983	0.2405	0.2769	6.0000
0.0000	1.0000	1.0000	1.0000	1.0000	1.0000	1.0000	7.0000
10.0000	1.0193	1.0077	1.0006	0.9991	0.9441	0.9463	7.0000
20.0000	1.0405	1.0129	1.0011	1.0004	0.9064	0.9085	7.0000
30.0000	1.0624	1.0193	0.9998	0.9991	0.8756	0.8769	7.0000
40.0000	1.0820	1.0257	0.9994	0.9994	0.8367	0.8505	7.0000
50.0000	1.0932	1.0322	0.9968	0.9994	0.7919	0.8286	7.0000
60.0000	1.1264	1.0399	0.9940	1.0002	0.7588	0.8119	7.0000
70.0000	1.1509	1.0476	0.9879	1.0009	0.7554	0.7968	7.0000
80.0000	1.1643	1.0553	0.9863	1.0050	0.7202	0.7798	7.0000
90.0000	1.1929	1.0644	0.9876	1.0117	0.6841	0.7636	7.0000
100.0000	1.2073	1.0721	0.9797	1.0175	0.6563	0.7449	7.0000
110.0000	1.2182	1.0798	0.9790	1.0158	0.6482	0.7274	7.0000
120.0000	1.2324	1.0875	0.9767	1.0112	0.6350	0.7091	7.0000
130.0000	1.2506	1.0952	0.9747	1.0076	0.6069	0.6927	7.0000
140.0000	1.2648	1.1030	0.9743	1.0067	0.5981	0.6783	7.0000
150.0000	1.2768	1.1107	0.9727	1.0039	0.5787	0.6642	7.0000
160.0000	1.2880	1.1197	0.9720	1.0021	0.5697	0.6506	7.0000
170.0000	1.3038	1.1274	0.9714	1.0004	0.5610	0.6387	7.0000
180.0000	1.3201	1.1351	0.9707	0.9976	0.5521	0.6274	7.0000
190.0000	1.3369	1.1429	0.9706	0.9955	0.5429	0.6161	7.0000
200.0000	1.3534	1.1506	0.9701	0.9940	0.5343	0.6061	7.0000
210.0000	1.3698	1.1583	0.9694	0.9920	0.5247	0.5940	7.0000
220.0000	1.3862	1.1660	0.9682	0.9892	0.5168	0.5850	7.0000
230.0000	1.4027	1.1725	0.9677	0.9873	0.5078	0.5740	7.0000
240.0000	1.4155	1.1789	0.9650	0.9802	0.5005	0.5647	7.0000
250.0000	1.4288	1.1853	0.9625	0.9743	0.4919	0.5542	7.0000
260.0000	1.4431	1.1918	0.9605	0.9700	0.4834	0.5452	7.0000
270.0000	1.4556	1.1982	0.9599	0.9674	0.4750	0.5344	7.0000
280.0000	1.4704	1.2046	0.9591	0.9653	0.4662	0.5249	7.0000
290.0000	1.4834	1.2098	0.9575	0.9627	0.4573	0.5157	7.0000
300.0000	1.5018	1.2149	0.9552	0.9595	0.4469	0.5046	7.0000
310.0000	1.5250	1.2201	0.9546	0.9581	0.4382	0.4956	7.0000
320.0000	1.5481	1.2239	0.9539	0.9573	0.4310	0.4879	7.0000
330.0000	1.5569	1.2291	0.9532	0.9566	0.4262	0.4833	7.0000
340.0000	1.5644	1.2329	0.9539	0.9573	0.4190	0.4761	7.0000
350.0000	1.5833	1.2368	0.9535	0.9573	0.4123	0.4692	7.0000
360.0000	1.5949	1.2407	0.9531	0.9571	0.4064	0.4633	7.0000

370.0000	1.6015	1.2445	0.9524	0.9562	0.4004	0.4571	7.0000
380.0000	1.6101	1.2484	0.9526	0.9571	0.3954	0.4522	7.0000
390.0000	1.6347	1.2523	0.9503	0.9540	0.3899	0.4466	7.0000
400.0000	1.6578	1.2574	0.9499	0.9534	0.3843	0.4406	7.0000
410.0000	1.6655	1.2625	0.9497	0.9538	0.3793	0.4355	7.0000
420.0000	1.6933	1.2664	0.9489	0.9530	0.3775	0.4337	7.0000
430.0000	1.7346	1.2716	0.9492	0.9534	0.3738	0.4301	7.0000
440.0000	1.7590	1.2716	0.9482	0.9528	0.3693	0.4252	7.0000
450.0000	1.7702	1.2754	0.9473	0.9517	0.3675	0.4232	7.0000
460.0000	1.7897	1.2819	0.9472	0.9525	0.3648	0.4201	7.0000
470.0000	1.7879	1.2870	0.9482	0.9545	0.3617	0.4170	7.0000
480.0000	1.7836	1.2921	0.9466	0.9521	0.3597	0.4150	7.0000
490.0000	1.8169	1.2973	0.9467	0.9530	0.3567	0.4116	7.0000
500.0000	1.8218	1.3012	0.9466	0.9538	0.3556	0.4103	7.0000
510.0000	1.8443	1.3063	0.9446	0.9510	0.3534	0.4078	7.0000
520.0000	1.8457	1.3102	0.9446	0.9495	0.3505	0.4047	7.0000
530.0000	1.8581	1.3153	0.9433	0.9526	0.3495	0.4036	7.0000
540.0000	1.8655	1.3192	0.9447	0.9517	0.3475	0.4011	7.0000
550.0000	1.8689	1.3127	0.9439	0.9517	0.3458	0.3995	7.0000
560.0000	1.8793	1.3166	0.9437	0.9530	0.3439	0.3975	7.0000
570.0000	1.8846	1.3218	0.9439	0.9530	0.3419	0.3949	7.0000
580.0000	1.8960	1.3256	0.9432	0.9530	0.3388	0.3916	7.0000
590.0000	1.9078	1.3295	0.9431	0.9528	0.3380	0.3908	7.0000
600.0000	1.9155	1.3308	0.9428	0.9540	0.3369	0.3893	7.0000
0.0000	1.0000	1.0000	1.0000	1.0000	1.0000	1.0000	8.0000
10.0000	1.0039	1.0038	0.9853	0.9919	1.0156	0.9636	8.0000
20.0000	1.0047	1.0063	0.9674	0.9843	0.9830	0.9331	8.0000
30.0000	1.0072	1.0075	0.9562	0.9787	0.9472	0.9083	8.0000
40.0000	1.0141	1.0113	0.9388	0.9703	0.9153	0.8953	8.0000
50.0000	1.0163	1.0138	0.9173	0.9610	0.9104	0.8804	8.0000
60.0000	1.0220	1.0151	0.9088	0.9573	0.8927	0.8644	8.0000
70.0000	1.0252	1.0189	0.8946	0.9506	0.8728	0.8525	8.0000
80.0000	1.0304	1.0214	0.8818	0.9445	0.8586	0.8437	8.0000
90.0000	1.0332	1.0252	0.8728	0.9408	0.8519	0.8325	8.0000
100.0000	1.0398	1.0289	0.8621	0.9379	0.8408	0.8213	8.0000
110.0000	1.0408	1.0314	0.8461	0.9332	0.8280	0.8260	8.0000
120.0000	1.0476	1.0365	0.8371	0.9299	0.8265	0.8135	8.0000
130.0000	1.0502	1.0390	0.8236	0.9242	0.8104	0.8036	8.0000
140.0000	1.0534	1.0428	0.8088	0.9149	0.8022	0.7988	8.0000
150.0000	1.0588	1.0465	0.7954	0.9045	0.7951	0.7890	8.0000
160.0000	1.0617	1.0503	0.7788	0.8904	0.7821	0.7774	8.0000
170.0000	1.0682	1.0528	0.7632	0.8754	0.7667	0.7727	8.0000
180.0000	1.0713	1.0566	0.7486	0.8613	0.7582	0.7587	8.0000
190.0000	1.0760	1.0616	0.7360	0.8488	0.7442	0.7516	8.0000
200.0000	1.0811	1.0667	0.7172	0.8334	0.7386	0.7448	8.0000
210.0000	1.0901	1.0755	0.7075	0.8243	0.7307	0.7356	8.0000
220.0000	1.1023	1.0868	0.6927	0.8124	0.7157	0.7299	8.0000
230.0000	1.1172	1.1044	0.6813	0.8035	0.7105	0.7238	8.0000
240.0000	1.1368	1.1258	0.6693	0.7940	0.7047	0.7159	8.0000
250.0000	1.1619	1.1509	0.6608	0.7872	0.6921	0.7068	8.0000
260.0000	1.1873	1.1748	0.6518	0.7792	0.6837	0.6962	8.0000
270.0000	1.2216	1.2101	0.6444	0.7719	0.6731	0.6871	8.0000
280.0000	1.2548	1.2415	0.6400	0.7661	0.6593	0.6792	8.0000
290.0000	1.2904	1.2755	0.6365	0.7601	0.6523	0.6721	8.0000
300.0000	1.3244	1.3082	0.6338	0.7548	0.6453	0.6636	8.0000
310.0000	1.3642	1.3472	0.6321	0.7514	0.6363	0.6565	8.0000
320.0000	1.3990	1.3862	0.6282	0.7439	0.6281	0.6517	8.0000
330.0000	1.4318	1.4214	0.6263	0.7405	0.6213	0.6446	8.0000

340.0000	1.4692	1.4579	0.6243	0.7361	0.6121	0.6361	8.0000
350.0000	1.5074	1.4969	0.6214	0.7308	0.6062	0.6323	8.0000
360.0000	1.5418	1.5333	0.6197	0.7272	0.6015	0.6279	8.0000
370.0000	1.5815	1.5723	0.6174	0.7224	0.5958	0.6205	8.0000
380.0000	1.6147	1.6075	0.6176	0.7220	0.5866	0.6188	8.0000
390.0000	1.6530	1.6465	0.6163	0.7192	0.5836	0.6113	8.0000
400.0000	1.6839	1.6780	0.6144	0.7148	0.5744	0.6079	8.0000
410.0000	1.7199	1.7145	0.6132	0.7124	0.5693	0.6045	8.0000
420.0000	1.7550	1.7509	0.6125	0.7110	0.5651	0.6014	8.0000
430.0000	1.7779	1.7736	0.6125	0.7110	0.5653	0.5994	8.0000
440.0000	1.8165	1.8126	0.6115	0.7088	0.5605	0.5953	8.0000
450.0000	1.8552	1.8541	0.6119	0.7098	0.5575	0.5912	8.0000
460.0000	1.8888	1.8881	0.6109	0.7081	0.5524	0.5895	8.0000
470.0000	1.9169	1.9195	0.6103	0.7077	0.5472	0.5875	8.0000
480.0000	1.9472	1.9497	0.6084	0.7045	0.5440	0.5838	8.0000
490.0000	1.9778	1.9811	0.6097	0.6974	0.5416	0.5793	8.0000
500.0000	2.0062	2.0101	0.6096	0.6973	0.5366	0.5787	8.0000
510.0000	2.0335	2.0377	0.6096	0.6978	0.5304	0.5756	8.0000
520.0000	2.0564	2.0591	0.6094	0.6980	0.5296	0.5736	8.0000
530.0000	2.0788	2.0780	0.6093	0.6981	0.5256	0.5719	8.0000
540.0000	2.1049	2.1044	0.6086	0.6973	0.5224	0.5688	8.0000
550.0000	2.1380	2.1371	0.6075	0.6961	0.5201	0.5637	8.0000
560.0000	2.1653	2.1610	0.6071	0.6960	0.5159	0.5620	8.0000
570.0000	2.1950	2.1912	0.6059	0.6941	0.5088	0.5603	8.0000
580.0000	2.2170	2.2101	0.6075	0.6971	0.5063	0.5579	8.0000
590.0000	2.2358	2.2277	0.6065	0.6960	0.5041	0.5549	8.0000
600.0000	2.2564	2.2465	0.6046	0.6930	0.5010	0.5532	8.0000
0.0000	1.0000	1.0000	1.0000	1.0000	1.0000	1.0000	9.0000
10.0000	1.0028	1.0037	0.9962	1.0006	0.9858	0.9237	9.0000
20.0000	1.0066	1.0087	0.9930	1.0016	0.9159	0.8658	9.0000
30.0000	1.0119	1.0136	0.9895	1.0039	0.8495	0.8188	9.0000
40.0000	1.0181	1.0211	0.9894	1.0142	0.7935	0.7785	9.0000
50.0000	1.0266	1.0310	0.9871	1.0353	0.7562	0.7418	9.0000
60.0000	1.0355	1.0434	0.9870	1.0676	0.7141	0.7118	9.0000
70.0000	1.0454	1.0582	0.9862	1.1142	0.6817	0.6832	9.0000
80.0000	1.0552	1.0706	0.9848	1.1515	0.6547	0.6573	9.0000
90.0000	1.0657	1.0843	0.9846	1.1812	0.6307	0.6334	9.0000
100.0000	1.0765	1.0967	0.9825	1.1906	0.6025	0.6076	9.0000
110.0000	1.0875	1.1103	0.9812	1.1981	0.5807	0.5934	9.0000
120.0000	1.0972	1.1202	0.9812	1.2055	0.5562	0.5746	9.0000
130.0000	1.1090	1.1314	0.9787	1.2087	0.5419	0.5574	9.0000
140.0000	1.1190	1.1413	0.9770	1.2133	0.5233	0.5404	9.0000
150.0000	1.1295	1.1537	0.9757	1.2172	0.5063	0.5271	9.0000
160.0000	1.1398	1.1636	0.9727	1.2201	0.4905	0.5136	9.0000
170.0000	1.1496	1.1760	0.9719	1.2227	0.4775	0.5058	9.0000
180.0000	1.1602	1.1859	0.9715	1.2249	0.4639	0.4937	9.0000
190.0000	1.1697	1.1970	0.9701	1.2265	0.4553	0.4825	9.0000
200.0000	1.1789	1.2082	0.9679	1.2265	0.4430	0.4735	9.0000
210.0000	1.1895	1.2193	0.9673	1.2294	0.4321	0.4641	9.0000
220.0000	1.1977	1.2305	0.9636	1.2252	0.4233	0.4589	9.0000
230.0000	1.2066	1.2429	0.9621	1.2252	0.4141	0.4545	9.0000
240.0000	1.2146	1.2528	0.9600	1.2230	0.4082	0.4472	9.0000
250.0000	1.2225	1.2615	0.9558	1.2159	0.4034	0.4405	9.0000
260.0000	1.2307	1.2714	0.9527	1.2094	0.3962	0.4314	9.0000
270.0000	1.2383	1.2800	0.9501	1.2045	0.3898	0.4250	9.0000
280.0000	1.2476	1.2912	0.9501	1.2016	0.3817	0.4192	9.0000
290.0000	1.2577	1.3036	0.9487	1.1981	0.3751	0.4144	9.0000
300.0000	1.2683	1.3172	0.9486	1.1919	0.3687	0.4108	9.0000

310.0000	1.2832	1.3358	0.9482	1.1832	0.3627	0.4060	9.0000
320.0000	1.2986	1.3556	0.9476	1.1874	0.3578	0.4018	9.0000
330.0000	1.3180	1.3779	0.9469	1.1854	0.3518	0.3975	9.0000
340.0000	1.3396	1.4027	0.9456	1.1838	0.3464	0.3950	9.0000
350.0000	1.3638	1.4300	0.9448	1.1851	0.3409	0.3908	9.0000
360.0000	1.3860	1.4560	0.9441	1.1832	0.3369	0.3874	9.0000
370.0000	1.4141	1.4895	0.9435	1.1828	0.3321	0.3833	9.0000
380.0000	1.4429	1.5242	0.9432	1.1864	0.3286	0.3798	9.0000
390.0000	1.4754	1.5675	0.9425	1.1819	0.3242	0.3759	9.0000
400.0000	1.5070	1.6084	0.9387	1.1867	0.3205	0.3721	9.0000
410.0000	1.5434	1.6518	0.9381	1.1858	0.3168	0.3682	9.0000
420.0000	1.5818	1.6976	0.9359	1.1819	0.3127	0.3652	9.0000
430.0000	1.6190	1.7447	0.9327	1.1825	0.3091	0.3622	9.0000
440.0000	1.6589	1.7968	0.9326	1.1825	0.3060	0.3590	9.0000
450.0000	1.6952	1.8439	0.9307	1.1835	0.3031	0.3556	9.0000
460.0000	1.7367	1.8959	0.9288	1.1854	0.3000	0.3530	9.0000
470.0000	1.7768	1.9467	0.9285	1.1854	0.2966	0.3498	9.0000
480.0000	1.8142	1.9938	0.9250	1.1851	0.2941	0.3471	9.0000
490.0000	1.8520	2.0397	0.9222	1.1890	0.2912	0.3425	9.0000
500.0000	1.8893	2.0880	0.9195	1.1874	0.2887	0.3409	9.0000
510.0000	1.9273	2.1351	0.9147	1.1417	0.2845	0.3388	9.0000
520.0000	1.9642	2.1772	0.9129	1.1443	0.2834	0.3372	9.0000
530.0000	2.0033	2.2255	0.9108	1.1427	0.2814	0.3349	9.0000
540.0000	2.0367	2.2652	0.9088	1.1456	0.2799	0.3324	9.0000
550.0000	2.0678	2.3024	0.9081	1.1456	0.2778	0.3297	9.0000
560.0000	2.0993	2.3383	0.9062	1.1437	0.2757	0.3278	9.0000
570.0000	2.1300	2.3767	0.9036	1.1450	0.2737	0.3260	9.0000
580.0000	2.1572	2.4077	0.9006	1.1466	0.2720	0.3239	9.0000
590.0000	2.1826	2.4387	0.8993	1.1524	0.2699	0.3212	9.0000
600.0000	2.2132	2.4758	0.8978	1.1528	0.2682	0.3205	9.0000

Al+Hg

t (s)	RBH-MFI	RBH-R	Calcein-MFI	Calcein-G	MDAC-MFI	MDAC-B	TYPE
0.0000	1.0000	1.0000	1.0000	1.0000	1.0000	1.0000	1.0000
10.0000	0.9964	0.9964	0.9995	1.0021	0.9436	0.9636	1.0000
20.0000	0.9979	0.9973	0.9981	1.0032	0.8999	0.9336	1.0000
30.0000	0.9999	0.9991	0.9909	0.9966	0.8630	0.9071	1.0000
40.0000	1.0024	1.0036	0.9814	0.9916	0.8347	0.8849	1.0000
50.0000	1.0046	1.0072	0.9968	0.9874	0.8111	0.8649	1.0000
60.0000	1.0046	1.0081	0.9942	0.9806	0.7908	0.8464	1.0000
70.0000	1.0108	1.0153	0.9955	0.9739	0.7706	0.8281	1.0000
80.0000	1.0119	1.0180	0.9902	0.9661	0.7528	0.8113	1.0000
90.0000	1.0214	1.0297	1.0002	0.9606	0.7341	0.7926	1.0000
100.0000	1.0295	1.0396	0.9972	0.9528	0.7165	0.7743	1.0000
110.0000	1.0397	1.0514	0.9968	0.9516	0.6994	0.7564	1.0000
120.0000	1.0517	1.0658	0.9950	0.9501	0.6844	0.7404	1.0000
130.0000	1.0623	1.0784	0.9936	0.9505	0.6680	0.7223	1.0000
140.0000	1.0741	1.0937	0.9925	0.9510	0.6531	0.7065	1.0000
150.0000	1.0829	1.1045	0.9899	0.9499	0.6378	0.6896	1.0000
160.0000	1.0904	1.1117	0.9873	0.9484	0.6246	0.6751	1.0000
170.0000	1.1011	1.1225	0.9838	0.9451	0.6116	0.6612	1.0000
180.0000	1.1184	1.1414	0.9814	0.9432	0.5985	0.6469	1.0000
190.0000	1.1376	1.1631	0.9792	0.9417	0.5842	0.6312	1.0000
200.0000	1.1626	1.1910	0.9774	0.9406	0.5731	0.6191	1.0000
210.0000	1.2070	1.2405	0.9794	0.9436	0.5622	0.6069	1.0000
220.0000	1.3065	1.3523	0.9788	0.9427	0.5531	0.5964	1.0000
230.0000	1.3875	1.5000	0.9771	0.9390	0.5431	0.5851	1.0000
240.0000	1.4027	1.6793	0.9822	0.9453	0.5345	0.5755	1.0000
250.0000	1.4560	1.8946	0.9877	0.9535	0.5248	0.5643	1.0000

260.0000	1.4947	2.0477	0.9931	0.9613	0.5186	0.5572	1.0000
270.0000	1.5725	2.1351	1.0005	0.9718	0.5157	0.5541	1.0000
280.0000	1.7001	2.1838	1.0107	0.9859	0.5077	0.5445	1.0000
290.0000	1.7675	2.1991	1.0179	0.9962	0.4972	0.5319	1.0000
300.0000	1.8499	2.2126	1.0250	1.0044	0.4856	0.5177	1.0000
310.0000	1.9082	2.2081	1.0392	1.0147	0.4748	0.5047	1.0000
320.0000	2.0064	2.2000	1.0523	1.0223	0.4626	0.4898	1.0000
330.0000	2.0176	2.1874	1.0667	1.0290	0.4506	0.4754	1.0000
340.0000	2.0183	2.1640	1.0746	1.0324	0.4407	0.4635	1.0000
350.0000	2.0203	2.1441	1.0845	1.0366	0.4303	0.4509	1.0000
360.0000	2.0217	2.1604	1.0878	1.0385	0.4203	0.4387	1.0000
370.0000	2.0222	2.1703	1.0965	1.0421	0.4125	0.4292	1.0000
380.0000	2.0225	2.1712	1.1076	1.0469	0.4046	0.4197	1.0000
390.0000	2.0246	2.1721	1.1066	1.0476	0.3986	0.4122	1.0000
400.0000	2.0253	2.1604	1.1092	1.0493	0.3906	0.4027	1.0000
410.0000	2.0285	2.1613	1.1087	1.0501	0.3838	0.3948	1.0000
420.0000	2.0293	2.1586	1.1128	1.0526	0.3777	0.3875	1.0000
430.0000	2.0293	2.1622	1.1076	1.0520	0.3711	0.3794	1.0000
440.0000	2.0317	2.1703	1.1151	1.0550	0.3660	0.3732	1.0000
450.0000	2.0339	2.1586	1.1143	1.0554	0.3594	0.3652	1.0000
460.0000	2.0347	2.1730	1.1121	1.0556	0.3535	0.3583	1.0000
470.0000	2.0349	2.1685	1.1111	1.0560	0.3489	0.3526	1.0000
480.0000	2.0368	2.1847	1.1111	1.0568	0.3444	0.3471	1.0000
490.0000	2.0368	2.1757	1.1013	1.0543	0.3396	0.3417	1.0000
500.0000	2.0428	2.1748	1.0999	1.0547	0.3370	0.3386	1.0000
510.0000	2.0449	2.1739	1.0977	1.0541	0.3322	0.3330	1.0000
520.0000	2.0451	2.1775	1.0987	1.0550	0.3289	0.3298	1.0000
530.0000	2.0457	2.1874	1.0981	1.0554	0.3271	0.3284	1.0000
540.0000	2.0526	2.1865	1.1029	1.0278	0.3245	0.3252	1.0000
550.0000	2.0552	2.1955	1.1008	1.0278	0.3242	0.3255	1.0000
560.0000	2.0588	2.2018	1.1001	1.0288	0.3222	0.3236	1.0000
570.0000	2.0601	2.1973	1.0966	1.0312	0.3225	0.3243	1.0000
580.0000	2.0617	2.2045	1.0954	1.0318	0.3211	0.3232	1.0000
590.0000	2.0658	2.2108	1.0888	1.0293	0.3197	0.3216	1.0000
600.0000	2.0783	2.2054	1.0743	1.0238	0.3164	0.3174	1.0000
0.0000	1.0000	1.0000	1.0000	1.0000	1.0000	1.0000	2.0000
10.0000	1.0815	1.0727	0.9923	0.9804	0.9192	0.8430	2.0000
20.0000	1.1842	1.1654	0.9896	0.9718	0.8764	0.7956	2.0000
30.0000	1.2415	1.2202	0.9891	0.9666	0.8164	0.7490	2.0000
40.0000	1.2841	1.2645	0.9860	0.9593	0.7443	0.6829	2.0000
50.0000	1.3242	1.3045	0.9827	0.9534	0.7063	0.6471	2.0000
60.0000	1.3521	1.3277	0.9774	0.9466	0.6754	0.6155	2.0000
70.0000	1.3705	1.3488	0.9723	0.9376	0.6506	0.5910	2.0000
80.0000	1.3754	1.3572	0.9652	0.9252	0.6277	0.5703	2.0000
90.0000	1.3735	1.3562	0.9567	0.9099	0.6076	0.5526	2.0000
100.0000	1.3724	1.3551	0.9500	0.8989	0.5915	0.5391	2.0000
110.0000	1.3707	1.3530	0.9500	0.8939	0.5748	0.5251	2.0000
120.0000	1.3694	1.3551	0.9492	0.8880	0.5610	0.5137	2.0000
130.0000	1.3686	1.3562	0.9485	0.8833	0.5490	0.5037	2.0000
140.0000	1.3676	1.3593	0.9477	0.8787	0.5372	0.4942	2.0000
150.0000	1.3667	1.3604	0.9484	0.8769	0.5275	0.4863	2.0000
160.0000	1.3707	1.3646	0.9481	0.8741	0.5158	0.4765	2.0000
170.0000	1.3931	1.3867	0.9481	0.8723	0.5051	0.4673	2.0000
180.0000	1.4125	1.4078	0.9474	0.8698	0.4933	0.4568	2.0000
190.0000	1.4364	1.4320	0.9475	0.8687	0.4824	0.4471	2.0000
200.0000	1.4566	1.4542	0.9463	0.8663	0.4724	0.4380	2.0000
210.0000	1.4692	1.4679	0.9456	0.8639	0.4618	0.4283	2.0000
220.0000	1.4776	1.4784	0.9461	0.8638	0.4523	0.4196	2.0000

230.0000	1.4793	1.4826	0.9468	0.8644	0.4426	0.4106	2.0000
240.0000	1.4930	1.5005	0.9472	0.8638	0.4321	0.4007	2.0000
250.0000	1.5049	1.5121	0.9467	0.8622	0.4202	0.3829	2.0000
260.0000	1.5206	1.5332	0.9483	0.8636	0.4104	0.3735	2.0000
270.0000	1.5340	1.5458	0.9505	0.8663	0.4006	0.3642	2.0000
280.0000	1.5471	1.5627	0.9291	0.8376	0.3910	0.3551	2.0000
290.0000	1.5610	1.5817	0.9322	0.8408	0.3829	0.3472	2.0000
300.0000	1.5722	1.5954	0.9339	0.8425	0.3748	0.3394	2.0000
310.0000	1.5959	1.6101	0.9368	0.8456	0.3666	0.3314	2.0000
320.0000	1.6113	1.6249	0.9382	0.8468	0.3587	0.3236	2.0000
330.0000	1.6235	1.6365	0.9412	0.8503	0.3506	0.3160	2.0000
340.0000	1.6464	1.6565	0.9430	0.8519	0.3426	0.3084	2.0000
350.0000	1.6796	1.6839	0.9456	0.8543	0.3346	0.3007	2.0000
360.0000	1.7193	1.7197	0.9465	0.8549	0.3272	0.2938	2.0000
370.0000	1.7795	1.7787	0.9493	0.8571	0.3201	0.2870	2.0000
380.0000	1.8604	1.8609	0.9500	0.8574	0.3132	0.2807	2.0000
390.0000	1.9599	1.9547	0.9516	0.8587	0.3064	0.2740	2.0000
400.0000	2.0844	2.0685	0.9549	0.8614	0.2997	0.2678	2.0000
410.0000	2.1857	2.1644	0.9545	0.8609	0.2938	0.2623	2.0000
420.0000	2.2734	2.2466	0.9558	0.8620	0.2875	0.2561	2.0000
430.0000	2.3205	2.2993	0.9576	0.8638	0.2819	0.2508	2.0000
440.0000	2.3560	2.3340	0.9583	0.8642	0.2762	0.2456	2.0000
450.0000	2.3673	2.3467	0.9596	0.8654	0.2716	0.2413	2.0000
460.0000	2.3764	2.3562	0.9591	0.8654	0.2661	0.2360	2.0000
470.0000	2.3760	2.3562	0.9590	0.8655	0.2611	0.2311	2.0000
480.0000	2.3775	2.3562	0.9606	0.8673	0.2565	0.2268	2.0000
490.0000	2.3686	2.3477	0.9601	0.8673	0.2518	0.2224	2.0000
500.0000	2.3609	2.3404	0.9602	0.8677	0.2471	0.2180	2.0000
510.0000	2.3548	2.3330	0.9611	0.8690	0.2419	0.2131	2.0000
520.0000	2.3508	2.3309	0.9612	0.8698	0.2394	0.2108	2.0000
530.0000	2.3390	2.3203	0.9611	0.8696	0.2351	0.2068	2.0000
540.0000	2.3409	2.3203	0.9607	0.8700	0.2306	0.2023	2.0000
550.0000	2.3320	2.3119	0.9594	0.8695	0.2281	0.2001	2.0000
560.0000	2.3261	2.3024	0.9602	0.8703	0.2241	0.1964	2.0000
570.0000	2.3250	2.3014	0.9579	0.8684	0.2212	0.1938	2.0000
580.0000	2.3196	2.2993	0.9591	0.8704	0.2176	0.1902	2.0000
590.0000	2.3164	2.2950	0.9581	0.8700	0.2148	0.1877	2.0000
600.0000	2.3144	2.2961	0.9577	0.8701	0.2111	0.1841	2.0000
0.0000	1.0000	1.0000	1.0000	1.0000	1.0000	1.0000	3.0000
10.0000	1.0008	0.9987	0.9981	0.9939	0.9480	0.9557	3.0000
20.0000	1.0031	0.9987	1.0076	0.9938	0.9053	0.9198	3.0000
30.0000	1.0025	1.0000	1.0098	0.9923	0.8728	0.8918	3.0000
40.0000	1.0045	1.0013	1.0056	0.9890	0.8464	0.8695	3.0000
50.0000	1.0050	1.0013	1.0108	0.9871	0.8201	0.8483	3.0000
60.0000	1.0066	1.0026	1.0101	0.9832	0.7998	0.8323	3.0000
70.0000	1.0027	1.0000	1.0087	0.9788	0.7825	0.8192	3.0000
80.0000	1.0055	1.0013	1.0076	0.9704	0.7644	0.8135	3.0000
90.0000	1.0090	1.0000	1.0057	0.9660	0.7545	0.8075	3.0000
100.0000	1.0105	1.0039	1.0042	0.9642	0.7294	0.8068	3.0000
110.0000	1.0139	1.0052	1.0029	0.9631	0.7226	0.8046	3.0000
120.0000	1.0162	1.0065	1.0014	0.9620	0.7182	0.7997	3.0000
130.0000	1.0195	1.0078	1.0003	0.9610	0.7120	0.7929	3.0000
140.0000	1.0231	1.0116	0.9990	0.9596	0.6934	0.7844	3.0000
150.0000	1.0270	1.0129	0.9983	0.9588	0.6844	0.7740	3.0000
160.0000	1.0303	1.0155	0.9982	0.9589	0.6749	0.7658	3.0000
170.0000	1.0346	1.0194	0.9969	0.9573	0.6675	0.7599	3.0000
180.0000	1.0400	1.0220	0.9958	0.9559	0.6564	0.7506	3.0000
190.0000	1.0442	1.0271	0.9944	0.9542	0.6554	0.7528	3.0000

200.0000	1.0532	1.0349	0.9942	0.9538	0.6485	0.7470	3.0000
210.0000	1.0665	1.0452	0.9924	0.9503	0.6405	0.7396	3.0000
220.0000	1.0913	1.0672	0.9917	0.9482	0.6336	0.7335	3.0000
230.0000	1.1451	1.1163	0.9903	0.9447	0.6219	0.7214	3.0000
240.0000	1.2502	1.2093	0.9900	0.9427	0.5885	0.6832	3.0000
250.0000	1.4197	1.3553	0.9896	0.9406	0.5811	0.6747	3.0000
260.0000	1.6187	1.5220	0.9885	0.9374	0.5734	0.6660	3.0000
270.0000	1.8090	1.6835	0.9868	0.9342	0.5670	0.6588	3.0000
280.0000	2.0262	1.8786	0.9871	0.9343	0.5602	0.6508	3.0000
290.0000	2.2274	2.0581	0.9876	0.9355	0.5506	0.6394	3.0000
300.0000	2.3777	2.2054	0.9885	0.9369	0.5429	0.6304	3.0000
310.0000	2.5128	2.3152	0.9890	0.9379	0.5335	0.6193	3.0000
320.0000	2.6620	2.4238	0.9893	0.9383	0.5235	0.6075	3.0000
330.0000	2.7357	2.4922	0.9902	0.9396	0.5129	0.5950	3.0000
340.0000	2.7959	2.5478	0.9918	0.9427	0.5042	0.5847	3.0000
350.0000	2.8305	2.5814	0.9918	0.9427	0.4913	0.5693	3.0000
360.0000	2.8665	2.6137	0.9926	0.9436	0.4819	0.5581	3.0000
370.0000	2.8921	2.6370	0.9934	0.9450	0.4613	0.5203	3.0000
380.0000	2.8941	2.6408	0.9938	0.9457	0.4541	0.5124	3.0000
390.0000	2.8990	2.6447	0.9943	0.9465	0.4465	0.5037	3.0000
400.0000	2.9113	2.6576	0.9938	0.9455	0.4376	0.4931	3.0000
410.0000	2.9063	2.6537	0.9939	0.9455	0.4310	0.4856	3.0000
420.0000	2.9017	2.6499	0.9944	0.9463	0.4222	0.4750	3.0000
430.0000	2.9049	2.6537	0.9949	0.9471	0.4141	0.4656	3.0000
440.0000	2.8985	2.6512	0.9957	0.9484	0.4071	0.4574	3.0000
450.0000	2.8988	2.6512	0.9957	0.9482	0.4006	0.4496	3.0000
460.0000	2.8889	2.6447	0.9959	0.9486	0.3949	0.4429	3.0000
470.0000	2.8949	2.6499	0.9957	0.9481	0.3876	0.4342	3.0000
480.0000	2.9103	2.6654	0.9958	0.9481	0.3786	0.4235	3.0000
490.0000	2.9132	2.6667	0.9957	0.9479	0.3732	0.4170	3.0000
500.0000	2.9125	2.6667	0.9964	0.9490	0.3660	0.4083	3.0000
510.0000	2.9208	2.6744	0.9964	0.9489	0.3602	0.4015	3.0000
520.0000	2.9100	2.6654	0.9966	0.9492	0.3544	0.3944	3.0000
530.0000	2.8962	2.6563	0.9966	0.9490	0.3489	0.3875	3.0000
540.0000	2.9090	2.6654	0.9962	0.9486	0.3411	0.3781	3.0000
550.0000	2.8996	2.6563	0.9968	0.9497	0.3352	0.3709	3.0000
560.0000	2.9099	2.6667	0.9965	0.9492	0.3294	0.3638	3.0000
570.0000	2.9035	2.6602	0.9967	0.9494	0.3245	0.3581	3.0000
580.0000	2.9012	2.6589	0.9971	0.9498	0.3183	0.3504	3.0000
590.0000	2.8922	2.6512	0.9969	0.9495	0.3143	0.3454	3.0000
600.0000	2.8815	2.6421	0.9973	0.9503	0.3085	0.3382	3.0000
0.0000	1.0000	1.0000	1.0000	1.0000	1.0000	1.0000	4.0000
10.0000	0.9985	1.0000	0.9976	0.9988	0.9429	0.8962	4.0000
20.0000	1.0014	1.0000	0.9978	0.9983	0.9108	0.8600	4.0000
30.0000	1.0050	1.0030	0.9979	0.9992	0.8812	0.8341	4.0000
40.0000	1.0078	1.0046	0.9980	0.9984	0.8579	0.8149	4.0000
50.0000	1.0094	1.0061	0.9973	0.9977	0.8353	0.7989	4.0000
60.0000	1.0127	1.0091	0.9960	0.9953	0.8171	0.7847	4.0000
70.0000	1.0160	1.0107	0.9952	0.9942	0.7999	0.7740	4.0000
80.0000	1.0189	1.0137	0.9944	0.9928	0.7851	0.7677	4.0000
90.0000	1.0259	1.0168	0.9942	0.9907	0.7694	0.7634	4.0000
100.0000	1.0281	1.0183	0.9945	0.9907	0.7559	0.7594	4.0000
110.0000	1.0330	1.0229	0.9953	0.9924	0.7423	0.7589	4.0000
120.0000	1.0365	1.0259	0.9942	0.9925	0.7293	0.7625	4.0000
130.0000	1.0418	1.0305	0.9929	0.9916	0.7156	0.7630	4.0000
140.0000	1.0441	1.0351	0.9913	0.9907	0.7045	0.7664	4.0000
150.0000	1.0464	1.0381	0.9882	0.9862	0.6956	0.7688	4.0000
160.0000	1.0494	1.0427	0.9867	0.9848	0.6852	0.7664	4.0000

170.0000	1.0542	1.0457	0.9851	0.9830	0.6786	0.7634	4.0000
180.0000	1.0570	1.0503	0.9850	0.9835	0.6748	0.7556	4.0000
190.0000	1.0613	1.0534	0.9822	0.9771	0.6709	0.7447	4.0000
200.0000	1.0677	1.0610	0.9803	0.9715	0.6665	0.7306	4.0000
210.0000	1.0831	1.0747	0.9785	0.9656	0.6605	0.7147	4.0000
220.0000	1.1522	1.1296	0.9784	0.9633	0.6552	0.7009	4.0000
230.0000	1.5016	1.4665	0.9802	0.9650	0.6498	0.6885	4.0000
240.0000	2.3031	2.2348	0.9833	0.9692	0.6418	0.6753	4.0000
250.0000	3.0911	2.9863	0.9872	0.9750	0.6343	0.6652	4.0000
260.0000	3.7478	3.6479	0.9916	0.9866	0.6254	0.6560	4.0000
270.0000	4.2267	4.1524	0.9953	0.9980	0.6130	0.6442	4.0000
280.0000	4.4928	4.4649	0.9987	1.0053	0.5995	0.6325	4.0000
290.0000	4.7048	4.6220	1.0024	1.0103	0.5833	0.6174	4.0000
300.0000	4.7219	4.6555	1.0110	1.0151	0.5639	0.5994	4.0000
310.0000	4.6677	4.6189	1.0192	1.0149	0.5430	0.5797	4.0000
320.0000	4.5472	4.5107	1.0160	1.0107	0.5210	0.5578	4.0000
330.0000	4.4078	4.3613	1.0085	1.0104	0.4979	0.5345	4.0000
340.0000	4.2675	4.2287	0.9994	1.0104	0.4763	0.5121	4.0000
350.0000	4.0931	4.0732	0.9983	1.0078	0.4585	0.4929	4.0000
360.0000	4.2131	4.1845	1.0000	1.0067	0.4414	0.4749	4.0000
370.0000	4.1825	4.1570	1.0003	1.0121	0.4278	0.4604	4.0000
380.0000	4.1471	4.1204	1.0030	1.0058	0.4169	0.4488	4.0000
390.0000	4.1138	4.0899	1.0158	1.0146	0.4057	0.4370	4.0000
400.0000	4.0861	4.0625	1.0162	1.0171	0.3953	0.4260	4.0000
410.0000	4.0610	4.0366	1.0167	1.0179	0.3860	0.4158	4.0000
420.0000	4.0403	4.0183	1.0167	1.0180	0.3755	0.4043	4.0000
430.0000	4.0087	3.9863	1.0175	1.0188	0.3658	0.3935	4.0000
440.0000	3.9916	3.9710	1.0160	1.0182	0.3553	0.3818	4.0000
450.0000	3.9600	3.9390	1.0138	1.0176	0.3462	0.3714	4.0000
460.0000	3.9500	3.9284	1.0107	1.0160	0.3383	0.3624	4.0000
470.0000	3.9351	3.9131	1.0080	1.0148	0.3304	0.3536	4.0000
480.0000	3.9261	3.9070	1.0076	1.0149	0.3223	0.3443	4.0000
490.0000	3.9261	3.8887	1.0052	1.0135	0.3158	0.3368	4.0000
500.0000	3.9081	3.8796	1.0038	1.0129	0.3092	0.3291	4.0000
510.0000	3.8970	3.8659	1.0028	1.0123	0.3021	0.3209	4.0000
520.0000	3.8858	3.8659	1.0018	1.0120	0.2959	0.3138	4.0000
530.0000	3.8828	3.8537	1.0005	1.0115	0.2894	0.3062	4.0000
540.0000	3.8723	3.8506	0.9994	1.0107	0.2838	0.2999	4.0000
550.0000	3.8697	3.8460	0.9986	1.0103	0.2771	0.2919	4.0000
560.0000	3.8628	3.8430	0.9984	1.0107	0.2709	0.2849	4.0000
570.0000	3.8606	3.8354	0.9974	1.0100	0.2663	0.2795	4.0000
580.0000	3.8526	3.8125	0.9960	1.0087	0.2620	0.2744	4.0000
590.0000	3.8302	3.8140	0.9959	1.0089	0.2568	0.2681	4.0000
600.0000	3.8298	3.8125	0.9953	1.0081	0.2521	0.2628	4.0000
0.0000	1.0000	1.0000	1.0000	1.0000	1.0000	1.0000	5.0000
10.0000	1.0346	1.0081	0.9987	0.9985	0.9171	0.9439	5.0000
20.0000	1.0387	1.0162	0.9984	0.9992	0.8515	0.8953	5.0000
30.0000	1.0437	1.0260	0.9970	0.9941	0.7786	0.8357	5.0000
40.0000	1.0490	1.0341	0.9977	0.9938	0.7075	0.7726	5.0000
50.0000	1.0566	1.0422	0.9961	0.9911	0.6430	0.7106	5.0000
60.0000	1.0653	1.0503	0.9957	0.9906	0.5892	0.6562	5.0000
70.0000	1.0774	1.0601	0.9950	0.9874	0.5457	0.6112	5.0000
80.0000	1.0909	1.0698	0.9953	0.9857	0.5070	0.5704	5.0000
90.0000	1.1057	1.0812	0.9915	0.9787	0.4748	0.5368	5.0000
100.0000	1.1213	1.0958	0.9899	0.9762	0.4620	0.5079	5.0000
110.0000	1.1355	1.1120	0.9890	0.9713	0.4344	0.4778	5.0000
120.0000	1.1500	1.1282	0.9898	0.9711	0.4094	0.4501	5.0000
130.0000	1.1683	1.1477	0.9858	0.9642	0.3847	0.4229	5.0000

140.0000	1.1824	1.1672	0.9876	0.9670	0.3600	0.3959	5.0000
150.0000	1.2030	1.1932	0.9852	0.9636	0.3417	0.3755	5.0000
160.0000	1.2233	1.2159	0.9813	0.9590	0.3248	0.3570	5.0000
170.0000	1.2429	1.2403	0.9797	0.9580	0.3092	0.3398	5.0000
180.0000	1.2690	1.2695	0.9784	0.9577	0.2975	0.3268	5.0000
190.0000	1.2935	1.2938	0.9853	0.9551	0.2875	0.3159	5.0000
200.0000	1.3166	1.3198	0.9840	0.9491	0.2777	0.3050	5.0000
210.0000	1.3437	1.3490	0.9806	0.9455	0.2701	0.2965	5.0000
220.0000	1.3746	1.3815	0.9792	0.9401	0.2600	0.2856	5.0000
230.0000	1.4318	1.4123	0.9761	0.9406	0.2520	0.2771	5.0000
240.0000	1.4584	1.4399	0.9769	0.9436	0.2453	0.2705	5.0000
250.0000	1.4878	1.4692	0.9801	0.9472	0.2391	0.2642	5.0000
260.0000	1.5245	1.5049	0.9837	0.9524	0.2331	0.2579	5.0000
270.0000	1.5745	1.5536	0.9879	0.9573	0.2281	0.2523	5.0000
280.0000	1.6904	1.6672	0.9925	0.9619	0.2211	0.2445	5.0000
290.0000	1.8612	1.8231	0.9962	0.9647	0.2149	0.2372	5.0000
300.0000	1.9970	1.9464	0.9986	0.9656	0.2072	0.2299	5.0000
310.0000	2.0910	2.0422	1.0000	0.9687	0.2028	0.2224	5.0000
320.0000	2.1695	2.1445	1.0022	0.9708	0.1970	0.2153	5.0000
330.0000	2.2758	2.2890	1.0044	0.9723	0.1915	0.2078	5.0000
340.0000	2.4167	2.4692	1.0058	0.9734	0.1865	0.2020	5.0000
350.0000	2.5656	2.6266	1.0065	0.9747	0.1816	0.1951	5.0000
360.0000	2.7037	2.7597	1.0079	0.9746	0.1780	0.1915	5.0000
370.0000	2.8168	2.8701	1.0078	0.9751	0.1741	0.1857	5.0000
380.0000	2.8969	2.9221	1.0086	0.9747	0.1711	0.1827	5.0000
390.0000	2.9184	2.9464	1.0087	0.9747	0.1682	0.1782	5.0000
400.0000	2.9560	2.9870	1.0086	0.9774	0.1652	0.1757	5.0000
410.0000	2.9727	3.0032	1.0105	0.9774	0.1625	0.1716	5.0000
420.0000	2.9778	3.0081	1.0106	0.9769	0.1599	0.1684	5.0000
430.0000	2.9730	3.0049	1.0104	0.9788	0.1577	0.1655	5.0000
440.0000	2.9759	3.0081	1.0118	0.9785	0.1555	0.1626	5.0000
450.0000	2.9661	2.9984	1.0114	0.9802	0.1534	0.1596	5.0000
460.0000	2.9589	2.9919	1.0124	0.9785	0.1514	0.1572	5.0000
470.0000	2.9549	2.9886	1.0116	0.9798	0.1498	0.1546	5.0000
480.0000	2.9506	2.9821	1.0120	0.9798	0.1480	0.1524	5.0000
490.0000	2.9438	2.9789	1.0122	0.9782	0.1472	0.1514	5.0000
500.0000	2.9397	2.9724	1.0113	0.9767	0.1452	0.1482	5.0000
510.0000	2.9307	2.9643	1.0100	0.9783	0.1445	0.1478	5.0000
520.0000	2.9230	2.9578	1.0111	0.9760	0.1432	0.1461	5.0000
530.0000	2.9145	2.9513	1.0097	0.9777	0.1423	0.1446	5.0000
540.0000	2.9019	2.9367	1.0103	0.9785	0.1415	0.1436	5.0000
550.0000	2.8965	2.9302	1.0109	0.9752	0.1406	0.1426	5.0000
560.0000	2.8872	2.9237	1.0087	0.9754	0.1396	0.1412	5.0000
570.0000	2.8711	2.9042	1.0085	0.9749	0.1391	0.1404	5.0000
580.0000	2.8612	2.8945	1.0083	0.9759	0.1381	0.1392	5.0000
590.0000	2.8555	2.8896	1.0086	0.9759	0.1376	0.1383	5.0000
600.0000	2.8470	2.8815	1.0083	0.9757	0.1369	0.1375	5.0000
0.0000	1.0000	1.0000	1.0000	1.0000	1.0000	1.0000	6.0000
10.0000	1.0038	1.0031	0.9868	0.9601	0.9777	0.9814	6.0000
20.0000	1.0077	1.0046	0.9815	0.9563	0.9409	0.9615	6.0000
30.0000	1.0105	1.0076	0.9752	0.9559	0.9230	0.9505	6.0000
40.0000	1.0168	1.0092	0.9761	0.9549	0.8912	0.9433	6.0000
50.0000	1.0227	1.0122	0.9667	0.9509	0.8733	0.9415	6.0000
60.0000	1.0261	1.0153	0.9654	0.9514	0.8578	0.9335	6.0000
70.0000	1.0319	1.0198	0.9682	0.9504	0.8448	0.9284	6.0000
80.0000	1.0385	1.0244	0.9672	0.9481	0.8317	0.9235	6.0000
90.0000	1.0412	1.0290	0.9739	0.9469	0.8135	0.9131	6.0000
100.0000	1.0493	1.0336	0.9631	0.9469	0.8030	0.9052	6.0000

110.0000	1.0540	1.0382	0.9711	0.9438	0.7789	0.8901	6.0000
120.0000	1.0588	1.0427	0.9646	0.9396	0.7709	0.8824	6.0000
130.0000	1.0648	1.0473	0.9628	0.9344	0.7608	0.8742	6.0000
140.0000	1.0691	1.0519	0.9667	0.9339	0.7500	0.8650	6.0000
150.0000	1.0739	1.0550	0.9559	0.9292	0.7350	0.8540	6.0000
160.0000	1.0798	1.0611	0.9643	0.9275	0.7247	0.8440	6.0000
170.0000	1.0836	1.0641	0.9516	0.9240	0.7130	0.8324	6.0000
180.0000	1.0908	1.0718	0.9542	0.9226	0.7009	0.8192	6.0000
190.0000	1.0979	1.0779	0.9520	0.9219	0.6911	0.8084	6.0000
200.0000	1.1057	1.0840	0.9417	0.9164	0.6782	0.7931	6.0000
210.0000	1.1142	1.0916	0.9608	0.9110	0.6678	0.7806	6.0000
220.0000	1.1255	1.0992	0.9573	0.9063	0.6572	0.7688	6.0000
230.0000	1.1322	1.1053	0.9565	0.8997	0.6466	0.7568	6.0000
240.0000	1.1442	1.1160	0.9590	0.8935	0.6352	0.7443	6.0000
250.0000	1.1534	1.1267	0.9504	0.8881	0.6240	0.7313	6.0000
260.0000	1.1628	1.1344	0.9543	0.8796	0.6083	0.7127	6.0000
270.0000	1.1708	1.1420	0.9564	0.8758	0.5976	0.6997	6.0000
280.0000	1.1802	1.1511	0.9550	0.8737	0.5649	0.6495	6.0000
290.0000	1.1863	1.1573	0.9555	0.8709	0.5529	0.6347	6.0000
300.0000	1.1936	1.1664	0.9465	0.8695	0.5409	0.6196	6.0000
310.0000	1.1999	1.1725	0.9498	0.8704	0.5328	0.6100	6.0000
320.0000	1.2084	1.1771	0.9484	0.8709	0.5206	0.5955	6.0000
330.0000	1.2301	1.1847	0.9594	0.8697	0.5086	0.5812	6.0000
340.0000	1.2400	1.1924	0.9595	0.8702	0.5052	0.5766	6.0000
350.0000	1.2556	1.2076	0.9563	0.8711	0.4934	0.5613	6.0000
360.0000	1.2872	1.2366	0.9506	0.8709	0.4866	0.5519	6.0000
370.0000	1.3475	1.2962	0.9486	0.8692	0.4803	0.5464	6.0000
380.0000	1.4278	1.3756	0.9520	0.8697	0.4351	0.4917	6.0000
390.0000	1.5106	1.4626	0.9543	0.8702	0.4279	0.4834	6.0000
400.0000	1.5863	1.5496	0.9541	0.8706	0.4204	0.4748	6.0000
410.0000	1.6520	1.6260	0.9544	0.8692	0.4151	0.4687	6.0000
420.0000	1.7265	1.7206	0.9537	0.8678	0.4096	0.4626	6.0000
430.0000	1.8124	1.8229	0.9533	0.8657	0.4041	0.4563	6.0000
440.0000	1.9197	1.9450	0.9530	0.8643	0.3985	0.4499	6.0000
450.0000	2.0322	2.0672	0.9525	0.8636	0.3919	0.4424	6.0000
460.0000	2.1585	2.2168	0.9510	0.8621	0.3874	0.4373	6.0000
470.0000	2.2813	2.3588	0.9493	0.8586	0.3820	0.4312	6.0000
480.0000	2.4269	2.5099	0.9634	0.8560	0.3758	0.4241	6.0000
490.0000	2.5357	2.6229	0.9642	0.8520	0.3701	0.4177	6.0000
500.0000	2.6414	2.7405	0.9642	0.8508	0.3672	0.4147	6.0000
510.0000	2.7225	2.8275	0.9653	0.8496	0.3599	0.4061	6.0000
520.0000	2.7709	2.8824	0.9656	0.8480	0.3561	0.4020	6.0000
530.0000	2.8020	2.9160	0.9647	0.8454	0.3497	0.3947	6.0000
540.0000	2.8225	2.9420	0.9653	0.8458	0.3435	0.3880	6.0000
550.0000	2.8524	2.9771	0.9644	0.8440	0.3392	0.3831	6.0000
560.0000	2.8377	2.9634	0.9635	0.8428	0.3355	0.3786	6.0000
570.0000	2.8372	2.9634	0.9633	0.8425	0.3323	0.3753	6.0000
580.0000	2.8336	2.9603	0.9627	0.8425	0.3280	0.3702	6.0000
590.0000	2.8351	2.9649	0.9625	0.8421	0.3233	0.3651	6.0000
600.0000	2.8352	2.9683	0.9623	0.8418	0.3189	0.3600	6.0000
0.0000	1.0000	1.0000	1.0000	1.0000	1.0000	1.0000	7.0000
10.0000	1.0068	1.0017	1.0021	0.9981	0.9357	0.9331	7.0000
20.0000	1.0120	1.0051	1.0036	0.9986	0.8650	0.8613	7.0000
30.0000	1.0190	1.0102	1.0064	1.0021	0.7831	0.7779	7.0000
40.0000	1.0252	1.0153	1.0069	1.0019	0.7073	0.7005	7.0000
50.0000	1.0328	1.0204	1.0073	1.0021	0.6435	0.6346	7.0000
60.0000	1.0444	1.0255	1.0075	1.0019	0.5939	0.5835	7.0000
70.0000	1.0524	1.0323	1.0072	1.0005	0.5518	0.5397	7.0000

80.0000	1.0601	1.0408	1.0071	1.0005	0.5175	0.5037	7.0000
90.0000	1.0679	1.0493	1.0053	0.9982	0.4938	0.4784	7.0000
100.0000	1.0763	1.0578	1.0030	0.9956	0.4687	0.4525	7.0000
110.0000	1.0840	1.0646	1.0011	0.9959	0.4508	0.4337	7.0000
120.0000	1.0946	1.0748	0.9967	0.9944	0.4331	0.4152	7.0000
130.0000	1.1064	1.0867	0.9911	0.9933	0.4158	0.3968	7.0000
140.0000	1.1383	1.1122	0.9833	0.9926	0.4012	0.3817	7.0000
150.0000	1.2273	1.1888	0.9962	0.9884	0.3888	0.3684	7.0000
160.0000	1.3699	1.3112	0.9933	0.9852	0.3789	0.3582	7.0000
170.0000	1.5430	1.4728	0.9885	0.9799	0.3689	0.3472	7.0000
180.0000	1.7917	1.6922	0.9843	0.9762	0.3600	0.3373	7.0000
190.0000	2.0408	1.9388	0.9798	0.9732	0.3518	0.3284	7.0000
200.0000	2.3068	2.1990	0.9767	0.9742	0.3454	0.3211	7.0000
210.0000	2.5543	2.4575	0.9731	0.9847	0.3407	0.3159	7.0000
220.0000	2.6182	2.6718	0.9690	0.9979	0.3347	0.3095	7.0000
230.0000	2.8204	2.8163	0.9700	1.0180	0.3297	0.3045	7.0000
240.0000	2.9426	2.9269	0.9787	1.0400	0.3244	0.2989	7.0000
250.0000	3.0244	2.9847	0.9894	1.0617	0.3181	0.2924	7.0000
260.0000	3.0478	3.0034	1.0022	1.0748	0.3127	0.2866	7.0000
270.0000	3.0452	3.0136	1.0146	1.0836	0.3060	0.2794	7.0000
280.0000	3.0389	3.0017	1.0223	1.0900	0.2978	0.2704	7.0000
290.0000	3.0430	2.9677	1.0279	1.0963	0.2896	0.2615	7.0000
300.0000	3.0238	2.9490	1.0317	1.1007	0.2826	0.2542	7.0000
310.0000	2.9809	2.9405	1.0317	1.1074	0.2754	0.2469	7.0000
320.0000	2.9575	2.9303	1.0357	1.1111	0.2693	0.2408	7.0000
330.0000	2.9780	2.9048	1.0357	1.1164	0.2639	0.2352	7.0000
340.0000	2.9594	2.8827	1.0384	1.1219	0.2595	0.2303	7.0000
350.0000	2.9265	2.8656	1.0431	1.1256	0.2554	0.2261	7.0000
360.0000	2.8992	2.8367	1.0524	1.1288	0.2521	0.2225	7.0000
370.0000	2.8729	2.8044	1.0636	1.1323	0.2483	0.2184	7.0000
380.0000	2.8405	2.7874	1.0781	1.1351	0.2458	0.2154	7.0000
390.0000	2.8018	2.7704	1.0916	1.1357	0.2436	0.2128	7.0000
400.0000	2.7805	2.7551	1.1066	1.1388	0.2413	0.2104	7.0000
410.0000	2.7577	2.7330	1.1121	1.1386	0.2393	0.2082	7.0000
420.0000	2.7752	2.7177	1.1228	1.1401	0.2380	0.2069	7.0000
430.0000	2.7586	2.7126	1.1253	1.1415	0.2356	0.2044	7.0000
440.0000	2.7315	2.7279	1.1314	1.1422	0.2337	0.2022	7.0000
450.0000	2.7157	2.7143	1.1338	1.1431	0.2318	0.2003	7.0000
460.0000	2.7089	2.7194	1.1390	1.1438	0.2302	0.1987	7.0000
470.0000	2.7222	2.7126	1.1351	1.1441	0.2281	0.1964	7.0000
480.0000	2.7049	2.6888	1.1336	1.1443	0.2265	0.1949	7.0000
490.0000	2.7071	2.7143	1.1334	1.1461	0.2253	0.1938	7.0000
500.0000	2.6983	2.7041	1.1298	1.1454	0.2253	0.1925	7.0000
510.0000	2.6696	2.7058	1.1334	1.1446	0.2241	0.1905	7.0000
520.0000	2.6931	2.6973	1.1266	1.1431	0.2241	0.1888	7.0000
530.0000	2.6804	2.6888	1.1215	1.1418	0.2220	0.1877	7.0000
540.0000	2.6787	2.6956	1.1090	1.1418	0.2204	0.1860	7.0000
550.0000	2.6651	2.7092	1.0993	1.1415	0.2193	0.1847	7.0000
560.0000	2.6537	2.7109	1.0958	1.1425	0.2178	0.1836	7.0000
570.0000	2.6550	2.7262	1.0943	1.1462	0.2164	0.1817	7.0000
580.0000	2.6639	2.7024	1.0858	1.1445	0.2152	0.1806	7.0000
590.0000	2.6629	2.7126	1.0837	1.1452	0.2133	0.1793	7.0000
600.0000	2.6736	2.7211	1.0704	1.1445	0.2125	0.1787	7.0000
0.0000	1.0000	1.0000	1.0000	1.0000	1.0000	1.0000	8.0000
10.0000	1.0800	1.0752	0.9966	1.0047	0.9536	0.9559	8.0000
20.0000	1.1269	1.1289	0.9982	1.0111	0.9013	0.9227	8.0000
30.0000	1.1479	1.1659	0.9970	1.0456	0.8453	0.8802	8.0000
40.0000	1.1540	1.1909	0.9963	1.0473	0.7815	0.8273	8.0000

50.0000	1.1513	1.2124	0.9959	1.0460	0.7190	0.7761	8.0000
60.0000	1.1920	1.2315	0.9933	1.0413	0.6615	0.7259	8.0000
70.0000	1.1699	1.2446	0.9936	1.0418	0.6170	0.6859	8.0000
80.0000	1.1900	1.2613	0.9909	1.0372	0.5800	0.6570	8.0000
90.0000	1.2055	1.2828	0.9886	1.0334	0.5531	0.6330	8.0000
100.0000	1.2721	1.3043	0.9889	1.0334	0.5324	0.6140	8.0000
110.0000	1.2275	1.3305	0.9859	1.0240	0.5115	0.5968	8.0000
120.0000	1.2517	1.3520	0.9846	1.0215	0.4913	0.5813	8.0000
130.0000	1.2801	1.3735	0.9843	1.0212	0.4775	0.5684	8.0000
140.0000	1.2482	1.3914	0.9848	1.0218	0.4624	0.5547	8.0000
150.0000	1.2847	1.4057	0.9841	1.0209	0.4503	0.5423	8.0000
160.0000	1.2655	1.4165	0.9836	1.0204	0.4412	0.5324	8.0000
170.0000	1.2640	1.4296	0.9831	1.0193	0.4301	0.5205	8.0000
180.0000	1.2546	1.4391	0.9824	1.0187	0.4197	0.5094	8.0000
190.0000	1.2721	1.4523	0.9812	1.0169	0.4098	0.4990	8.0000
200.0000	1.2679	1.4630	0.9801	1.0152	0.4003	0.4883	8.0000
210.0000	1.2637	1.4737	0.9795	1.0138	0.3935	0.4813	8.0000
220.0000	1.2713	1.4893	0.9783	1.0106	0.3846	0.4719	8.0000
230.0000	1.2725	1.5060	0.9766	1.0063	0.3763	0.4635	8.0000
240.0000	1.2708	1.5179	0.9759	1.0044	0.3683	0.4552	8.0000
250.0000	1.2685	1.5274	0.9780	1.0065	0.3605	0.4476	8.0000
260.0000	1.2676	1.5406	0.9827	1.0125	0.3527	0.4400	8.0000
270.0000	1.2638	1.5477	0.9856	1.0168	0.3448	0.4321	8.0000
280.0000	1.2628	1.5561	0.9902	1.0229	0.3371	0.4240	8.0000
290.0000	1.2654	1.5704	0.9940	1.0286	0.3296	0.4159	8.0000
300.0000	1.2783	1.5990	0.9974	1.0339	0.3223	0.4081	8.0000
310.0000	1.2977	1.6492	1.0011	1.0399	0.3148	0.4007	8.0000
320.0000	1.3258	1.7315	1.0031	1.0433	0.3067	0.3929	8.0000
330.0000	1.3746	1.8425	1.0055	1.0475	0.2988	0.3850	8.0000
340.0000	1.5029	1.9773	1.0072	1.0506	0.2913	0.3779	8.0000
350.0000	1.6083	2.1169	1.0084	1.0531	0.2783	0.3698	8.0000
360.0000	1.7225	2.2804	1.0102	1.0562	0.2844	0.3777	8.0000
370.0000	1.8492	2.4296	1.0109	1.0579	0.2768	0.3693	8.0000
380.0000	1.9293	2.5418	1.0126	1.0609	0.2701	0.3614	8.0000
390.0000	1.9753	2.6062	1.0120	1.0603	0.2647	0.3556	8.0000
400.0000	1.9894	2.6265	1.0135	1.0630	0.2592	0.3488	8.0000
410.0000	1.9975	2.6372	1.0140	1.0642	0.2524	0.3412	8.0000
420.0000	1.9999	2.6408	1.0146	1.0656	0.2480	0.3356	8.0000
430.0000	2.0016	2.6420	1.0151	1.0666	0.2429	0.3298	8.0000
440.0000	1.9997	2.6432	1.0156	1.0679	0.2389	0.3250	8.0000
450.0000	1.9988	2.6468	1.0157	1.0680	0.2350	0.3207	8.0000
460.0000	1.9976	2.6504	1.0152	1.0674	0.2306	0.3156	8.0000
470.0000	1.9949	2.6516	1.0151	1.0675	0.2274	0.3118	8.0000
480.0000	1.9932	2.6527	1.0144	1.0666	0.2237	0.3072	8.0000
490.0000	1.9867	2.6492	1.0146	1.0671	0.2210	0.3045	8.0000
500.0000	1.9848	2.6504	1.0144	1.0668	0.2176	0.3004	8.0000
510.0000	1.9841	2.6539	1.0143	1.0669	0.2152	0.2979	8.0000
520.0000	1.9810	2.6516	1.0136	1.0660	0.2120	0.2943	8.0000
530.0000	1.9823	2.6575	1.0131	1.0655	0.2086	0.2903	8.0000
540.0000	1.9799	2.6575	1.0121	1.0639	0.2054	0.2865	8.0000
550.0000	1.9744	2.6504	1.0123	1.0644	0.2023	0.2832	8.0000
560.0000	1.9698	2.6456	1.0116	1.0634	0.1993	0.2806	8.0000
570.0000	1.9661	2.6444	1.0114	1.0631	0.1966	0.2766	8.0000
580.0000	1.9603	2.6384	1.0111	1.0628	0.1937	0.2743	8.0000
590.0000	1.9586	2.6384	1.0104	1.0618	0.1911	0.2708	8.0000
600.0000	1.9599	2.6420	1.0105	1.0623	0.1886	0.2682	8.0000
0.0000	1.0000	1.0000	1.0000	1.0000	1.0000	1.0000	9.0000
10.0000	1.0065	0.9984	1.0009	0.9965	0.9542	0.9546	9.0000

20.0000	1.0036	1.0000	1.0052	0.9884	0.9251	0.9245	9.0000
30.0000	1.0072	1.0000	1.0052	0.9833	0.8942	0.8892	9.0000
40.0000	1.0082	1.0016	1.0053	0.9796	0.8645	0.8562	9.0000
50.0000	1.0090	1.0016	1.0051	0.9757	0.8184	0.8080	9.0000
60.0000	1.0099	1.0048	1.0088	0.9745	0.7716	0.7602	9.0000
70.0000	1.0076	1.0064	1.0117	0.9723	0.7298	0.7185	9.0000
80.0000	1.0025	1.0064	1.0116	0.9701	0.6947	0.6845	9.0000
90.0000	1.0157	1.0080	1.0070	0.9669	0.6609	0.6522	9.0000
100.0000	1.0113	1.0080	1.0102	0.9663	0.6298	0.6214	9.0000
110.0000	1.0056	1.0128	1.0041	0.9629	0.6006	0.5926	9.0000
120.0000	1.0010	1.0112	0.9976	0.9602	0.5738	0.5654	9.0000
130.0000	0.9975	1.0128	0.9931	0.9585	0.5485	0.5402	9.0000
140.0000	1.0060	1.0160	0.9847	0.9560	0.5236	0.5153	9.0000
150.0000	1.0065	1.0208	0.9787	0.9544	0.5026	0.4942	9.0000
160.0000	1.0079	1.0240	0.9737	0.9529	0.4796	0.4707	9.0000
170.0000	1.0105	1.0256	0.9670	0.9511	0.4614	0.4522	9.0000
180.0000	1.0144	1.0256	0.9614	0.9494	0.4460	0.4361	9.0000
190.0000	1.0148	1.0272	0.9624	0.9504	0.4313	0.4217	9.0000
200.0000	1.0159	1.0272	0.9545	0.9480	0.4172	0.4074	9.0000
210.0000	1.0171	1.0319	0.9503	0.9472	0.4055	0.3954	9.0000
220.0000	1.0170	1.0335	0.9451	0.9447	0.3908	0.3801	9.0000
230.0000	1.0209	1.0335	0.9428	0.9428	0.3794	0.3687	9.0000
240.0000	1.0422	1.0383	0.9381	0.9374	0.3693	0.3588	9.0000
250.0000	1.0450	1.0479	0.9324	0.9300	0.3614	0.3508	9.0000
260.0000	1.0495	1.0831	0.9279	0.9244	0.3533	0.3427	9.0000
270.0000	1.0623	1.2460	0.9252	0.9217	0.3468	0.3360	9.0000
280.0000	1.0972	1.5990	0.9238	0.9215	0.3423	0.3315	9.0000
290.0000	1.3149	1.9888	0.9230	0.9229	0.3380	0.3272	9.0000
300.0000	1.7455	2.2764	0.9222	0.9246	0.3343	0.3231	9.0000
310.0000	2.2220	2.4169	0.9221	0.9268	0.3322	0.3207	9.0000
320.0000	2.5230	2.6214	0.9217	0.9285	0.3291	0.3170	9.0000
330.0000	2.6393	2.7939	0.9216	0.9300	0.3258	0.3132	9.0000
340.0000	2.8319	2.8610	0.9222	0.9323	0.3240	0.3115	9.0000
350.0000	2.9822	2.9744	0.9223	0.9333	0.3221	0.3091	9.0000
360.0000	3.0219	3.0783	0.9224	0.9345	0.3180	0.3046	9.0000
370.0000	3.1371	3.1901	0.9224	0.9355	0.3135	0.2996	9.0000
380.0000	3.2457	3.2204	0.9224	0.9359	0.3096	0.2953	9.0000
390.0000	3.3606	3.2141	0.9226	0.9376	0.3091	0.2949	9.0000
400.0000	3.3900	3.2492	0.9227	0.9398	0.3041	0.2897	9.0000
410.0000	3.3757	3.2764	0.9224	0.9428	0.3015	0.2869	9.0000
420.0000	3.4105	3.3019	0.9227	0.9492	0.2994	0.2848	9.0000
430.0000	3.4396	3.3179	0.9239	0.9620	0.2936	0.2785	9.0000
440.0000	3.4652	3.3131	0.9235	0.9750	0.2917	0.2764	9.0000
450.0000	3.4797	3.3211	0.9228	0.9846	0.2893	0.2740	9.0000
460.0000	3.5598	3.3403	0.9223	0.9924	0.2870	0.2716	9.0000
470.0000	3.5702	3.3435	0.9224	1.0057	0.2855	0.2697	9.0000
480.0000	3.5925	3.3530	0.9222	1.0181	0.2841	0.2684	9.0000
490.0000	3.5963	3.3562	0.9219	1.0289	0.2816	0.2656	9.0000
500.0000	3.6046	3.3626	0.9219	1.0415	0.2811	0.2650	9.0000
510.0000	3.6100	3.3738	0.9219	1.0513	0.2803	0.2641	9.0000
520.0000	3.6150	3.3738	0.9217	1.0537	0.2779	0.2615	9.0000
530.0000	3.6274	3.3626	0.9213	1.0518	0.2760	0.2596	9.0000
540.0000	3.6269	3.3706	0.9221	1.0521	0.2742	0.2576	9.0000
550.0000	3.6147	3.3642	0.9217	1.0506	0.2720	0.2553	9.0000
560.0000	3.6224	3.3674	0.9219	1.0513	0.2697	0.2529	9.0000
570.0000	3.6154	3.3818	0.9219	1.0511	0.2692	0.2520	9.0000
580.0000	3.6188	3.3770	0.9216	1.0505	0.2699	0.2531	9.0000
590.0000	3.6338	3.3802	0.9217	1.0513	0.2703	0.2531	9.0000

600.0000	3.6272	3.3818	0.9219	1.0525	0.2690	0.2520	9.0000

Cu+Hg

t （s)	RBH-MFI	RBH-R	Calcein-MFI	Calcein-G	MDAC-MFI	MDAC-B	TYPE
0.0000	1.0000	1.0000	1.0000	1.0000	1.0000	1.0000	1.0000
10.0000	0.9991	0.9988	1.0087	1.0211	0.9118	0.9308	1.0000
20.0000	0.9975	0.9965	1.0077	1.0383	0.8453	0.8725	1.0000
30.0000	0.9968	0.9953	1.0108	1.0556	0.7965	0.8302	1.0000
40.0000	0.9959	0.9917	1.0160	1.0666	0.7547	0.7924	1.0000
50.0000	0.9937	0.9917	1.0143	1.0735	0.7211	0.7621	1.0000
60.0000	1.0029	0.9953	1.0141	1.0832	0.6935	0.7351	1.0000
70.0000	1.0140	1.0059	1.0220	1.0567	0.6710	0.7109	1.0000
80.0000	1.0212	1.0142	1.0168	1.0601	0.6491	0.6865	1.0000
90.0000	1.0236	1.0225	1.0162	1.0614	0.6297	0.6638	1.0000
100.0000	1.0227	1.0272	1.0145	1.0625	0.6115	0.6429	1.0000
110.0000	1.0230	1.0331	1.0169	1.0646	0.5930	0.6213	1.0000
120.0000	1.0316	1.0426	1.0152	1.0644	0.5779	0.6040	1.0000
130.0000	1.0344	1.0532	1.0061	1.0605	0.5640	0.5880	1.0000
140.0000	1.0435	1.0638	1.0036	1.0608	0.5504	0.5729	1.0000
150.0000	1.0479	1.0792	0.9926	1.0543	0.5375	0.5584	1.0000
160.0000	1.0495	1.0910	0.9868	1.0508	0.5253	0.5446	1.0000
170.0000	1.0822	1.1076	0.9777	1.0439	0.5141	0.5326	1.0000
180.0000	1.0859	1.1217	0.9845	1.0403	0.5039	0.5214	1.0000
190.0000	1.0899	1.1371	0.9814	1.0351	0.4918	0.5086	1.0000
200.0000	1.1215	1.1655	0.9779	1.0269	0.4816	0.4975	1.0000
210.0000	1.1962	1.2624	0.9726	1.0228	0.4716	0.4868	1.0000
220.0000	1.4371	1.5449	0.9695	1.0265	0.4594	0.4734	1.0000
230.0000	1.8192	1.8913	0.9701	1.0342	0.4506	0.4637	1.0000
240.0000	2.0552	2.1773	0.9730	1.0439	0.4406	0.4534	1.0000
250.0000	2.2140	2.3440	0.9771	1.0538	0.4313	0.4439	1.0000
260.0000	2.2233	2.3463	0.9817	1.0696	0.4272	0.4399	1.0000
270.0000	2.2240	2.3475	1.0107	1.0849	0.4180	0.4304	1.0000
280.0000	2.2258	2.3475	1.0340	1.0995	0.4141	0.4265	1.0000
290.0000	2.2275	2.3570	1.0583	1.1118	0.4087	0.4207	1.0000
300.0000	2.2293	2.3582	1.0892	1.1256	0.4026	0.4140	1.0000
310.0000	2.2318	2.3582	1.1182	1.1376	0.3938	0.4040	1.0000
320.0000	2.2321	2.3582	1.1376	1.1456	0.3859	0.3949	1.0000
330.0000	2.2332	2.3582	1.1610	1.1551	0.3786	0.3864	1.0000
340.0000	2.2332	2.3629	1.1821	1.1637	0.3725	0.3796	1.0000
350.0000	2.2337	2.3641	1.1918	1.1689	0.3655	0.3721	1.0000
360.0000	2.2339	2.3641	1.2002	1.1736	0.3602	0.3664	1.0000
370.0000	2.2341	2.3652	1.2015	1.1760	0.3541	0.3594	1.0000
380.0000	2.2344	2.3652	1.2073	1.1799	0.3486	0.3534	1.0000
390.0000	2.2355	2.3652	1.2082	1.1822	0.3432	0.3473	1.0000
400.0000	2.2358	2.3688	1.2091	1.1848	0.3386	0.3422	1.0000
410.0000	2.2361	2.3688	1.2023	1.1850	0.3336	0.3363	1.0000
420.0000	2.2368	2.3688	1.1948	1.1846	0.3286	0.3308	1.0000
430.0000	2.2376	2.3700	1.1921	1.1853	0.3245	0.3264	1.0000
440.0000	2.2384	2.3700	1.1850	1.1838	0.3193	0.3206	1.0000
450.0000	2.2387	2.3700	1.1751	1.1816	0.3153	0.3161	1.0000
460.0000	2.2392	2.3700	1.1669	1.1805	0.3109	0.3112	1.0000
470.0000	2.2395	2.3712	1.1741	1.1846	0.3068	0.3066	1.0000
480.0000	2.2411	2.3712	1.1614	1.1809	0.3022	0.3015	1.0000
490.0000	2.2412	2.3712	1.1587	1.1799	0.2988	0.2978	1.0000
500.0000	2.2430	2.3723	1.1481	1.1756	0.2947	0.2930	1.0000
510.0000	2.2438	2.3735	1.1413	1.1728	0.2905	0.2884	1.0000
520.0000	2.2462	2.3747	1.1411	1.1734	0.2876	0.2851	1.0000
530.0000	2.2490	2.3747	1.1352	1.1725	0.2835	0.2802	1.0000
540.0000	2.2490	2.3747	1.1294	1.1719	0.2799	0.2763	1.0000

550.0000	2.2505	2.3759	1.1315	1.1760	0.2772	0.2737	1.0000
560.0000	2.2532	2.3759	1.1256	1.1801	0.2740	0.2697	1.0000
570.0000	2.2535	2.3771	1.1217	1.1894	0.2710	0.2666	1.0000
580.0000	2.2549	2.3771	1.1137	1.1982	0.2677	0.2632	1.0000
590.0000	2.2555	2.3771	1.1107	1.2055	0.2649	0.2598	1.0000
600.0000	2.2556	2.3771	1.1103	1.2148	0.2624	0.2571	1.0000
0.0000	1.0000	1.0000	1.0000	1.0000	1.0000	1.0000	2.0000
10.0000	1.0134	1.0193	0.9979	0.9642	0.9499	0.9561	2.0000
20.0000	1.0414	1.0499	0.9960	0.9534	0.9085	0.9158	2.0000
30.0000	1.0719	1.0884	0.9875	0.9490	0.8756	0.8846	2.0000
40.0000	1.1082	1.1293	0.9704	0.9452	0.8346	0.8489	2.0000
50.0000	1.1303	1.1689	0.9533	0.9398	0.7998	0.8177	2.0000
60.0000	1.1683	1.2098	0.9238	0.9296	0.7747	0.7960	2.0000
70.0000	1.2029	1.2483	0.9028	0.9200	0.7534	0.7772	2.0000
80.0000	1.2319	1.2823	0.8890	0.9092	0.7338	0.7588	2.0000
90.0000	1.2646	1.3231	0.8854	0.8995	0.7179	0.7445	2.0000
100.0000	1.2950	1.3617	0.8789	0.8877	0.7014	0.7292	2.0000
110.0000	1.3232	1.3980	0.8686	0.8745	0.6855	0.7142	2.0000
120.0000	1.3531	1.4354	0.8734	0.8716	0.6738	0.7027	2.0000
130.0000	1.3842	1.4762	0.8761	0.8681	0.6631	0.6923	2.0000
140.0000	1.4116	1.5034	0.8786	0.8650	0.6548	0.6846	2.0000
150.0000	1.4375	1.5374	0.8771	0.8634	0.6439	0.6744	2.0000
160.0000	1.4639	1.5703	0.8756	0.8637	0.6347	0.6658	2.0000
170.0000	1.4922	1.6032	0.8743	0.8643	0.6243	0.6556	2.0000
180.0000	1.5097	1.6281	0.8717	0.8646	0.6168	0.6486	2.0000
190.0000	1.5331	1.6565	0.8696	0.8657	0.6056	0.6371	2.0000
200.0000	1.5518	1.6814	0.8610	0.8634	0.5997	0.6312	2.0000
210.0000	1.5643	1.6984	0.8572	0.8650	0.5897	0.6208	2.0000
220.0000	1.5803	1.7211	0.8507	0.8654	0.5824	0.6135	2.0000
230.0000	1.5975	1.7426	0.8443	0.8643	0.5740	0.6044	2.0000
240.0000	1.6011	1.7494	0.8385	0.8615	0.5657	0.5956	2.0000
250.0000	1.6135	1.7642	0.8352	0.8584	0.5571	0.5870	2.0000
260.0000	1.6233	1.7800	0.8297	0.8534	0.5475	0.5793	2.0000
270.0000	1.6311	1.7914	0.8287	0.8544	0.5331	0.5699	2.0000
280.0000	1.6431	1.8095	0.8277	0.8566	0.5156	0.5562	2.0000
290.0000	1.6446	1.8118	0.8314	0.8630	0.5014	0.5430	2.0000
300.0000	1.6593	1.8118	0.8360	0.8696	0.4916	0.5321	2.0000
310.0000	1.6673	1.8209	0.8393	0.8751	0.4870	0.5260	2.0000
320.0000	1.6729	1.8311	0.8466	0.8819	0.4779	0.5155	2.0000
330.0000	1.6780	1.8379	0.8522	0.8870	0.4704	0.5073	2.0000
340.0000	1.6755	1.8379	0.8597	0.8927	0.4588	0.4943	2.0000
350.0000	1.6867	1.8526	0.8646	0.8969	0.4512	0.4863	2.0000
360.0000	1.7034	1.8776	0.8742	0.9033	0.4421	0.4767	2.0000
370.0000	1.7294	1.9116	0.8752	0.9054	0.4317	0.4652	2.0000
380.0000	1.7596	1.9603	0.8761	0.8674	0.4235	0.4563	2.0000
390.0000	1.7996	2.0227	0.8780	0.8685	0.4158	0.4477	2.0000
400.0000	1.8503	2.1043	0.8829	0.8696	0.4094	0.4404	2.0000
410.0000	1.9137	2.1995	0.8879	0.8714	0.4034	0.4337	2.0000
420.0000	1.9784	2.2925	0.8905	0.8725	0.3956	0.4247	2.0000
430.0000	2.0461	2.3696	0.8932	0.8740	0.3911	0.4195	2.0000
440.0000	2.1123	2.4422	0.8918	0.8740	0.3847	0.4120	2.0000
450.0000	2.1675	2.5193	0.8951	0.8753	0.3797	0.4059	2.0000
460.0000	2.2093	2.5805	0.8956	0.8762	0.3761	0.4018	2.0000
470.0000	2.2381	2.6224	0.8937	0.8760	0.3700	0.3944	2.0000
480.0000	2.2598	2.6531	0.8945	0.8773	0.3650	0.3886	2.0000
490.0000	2.2708	2.6701	0.8933	0.8775	0.3608	0.3836	2.0000
500.0000	2.2776	2.6803	0.8888	0.8767	0.3555	0.3770	2.0000
510.0000	2.2833	2.6905	0.8951	0.8799	0.3515	0.3722	2.0000

520.0000	2.2895	2.6961	0.8944	0.8802	0.3490	0.3693	2.0000
530.0000	2.2860	2.6939	0.8930	0.8802	0.3437	0.3630	2.0000
540.0000	2.2871	2.6950	0.8900	0.8802	0.3407	0.3595	2.0000
550.0000	2.2893	2.6973	0.8895	0.8813	0.3358	0.3538	2.0000
560.0000	2.2828	2.6893	0.8879	0.8811	0.3323	0.3493	2.0000
570.0000	2.2806	2.6859	0.8860	0.8815	0.3273	0.3437	2.0000
580.0000	2.2759	2.6848	0.8817	0.8815	0.3237	0.3394	2.0000
590.0000	2.2710	2.6757	0.8833	0.8830	0.3204	0.3354	2.0000
600.0000	2.2723	2.6780	0.8778	0.8815	0.3168	0.3309	2.0000
0.0000	1.0000	1.0000	1.0000	1.0000	1.0000	1.0000	3.0000
10.0000	0.9979	0.9975	1.0083	1.0082	0.9522	0.9200	3.0000
20.0000	0.9981	0.9963	1.0059	1.0070	0.9240	0.8974	3.0000
30.0000	0.9976	0.9963	1.0128	1.0061	0.8977	0.8780	3.0000
40.0000	0.9951	0.9938	1.0165	1.0052	0.8668	0.8552	3.0000
50.0000	0.9953	0.9951	1.0138	1.0043	0.8322	0.8278	3.0000
60.0000	0.9922	0.9926	1.0177	1.0061	0.7967	0.7980	3.0000
70.0000	0.9920	0.9938	1.0130	1.0086	0.7636	0.7687	3.0000
80.0000	0.9907	0.9938	1.0212	1.0170	0.7342	0.7415	3.0000
90.0000	0.9897	0.9926	1.0177	1.0347	0.7393	0.7153	3.0000
100.0000	0.9902	0.9951	0.9905	1.0284	0.7119	0.6926	3.0000
110.0000	0.9909	0.9951	0.9591	1.0154	0.6885	0.6715	3.0000
120.0000	0.9918	0.9963	0.9515	1.0057	0.6671	0.6528	3.0000
130.0000	0.9918	0.9988	0.9501	1.0023	0.6487	0.6346	3.0000
140.0000	1.0025	0.9988	0.9376	1.0000	0.6309	0.6186	3.0000
150.0000	1.0059	1.0025	0.9466	0.9986	0.6150	0.6031	3.0000
160.0000	1.0065	1.0025	0.9387	0.9964	0.5997	0.5882	3.0000
170.0000	1.0096	1.0049	0.9359	0.9941	0.5851	0.5733	3.0000
180.0000	1.0104	1.0074	0.9268	0.9923	0.5704	0.5594	3.0000
190.0000	1.0114	1.0074	0.9276	0.9893	0.5568	0.5458	3.0000
200.0000	1.0121	1.0086	0.9210	0.9850	0.5436	0.5325	3.0000
210.0000	1.0137	1.0123	0.9129	0.9791	0.5307	0.5205	3.0000
220.0000	1.0136	1.0111	0.9041	0.9703	0.5192	0.5090	3.0000
230.0000	1.0147	1.0135	0.8939	0.9560	0.5080	0.4966	3.0000
240.0000	1.0162	1.0160	0.8842	0.9433	0.4962	0.4852	3.0000
250.0000	1.0150	1.0148	0.8778	0.9367	0.4854	0.4751	3.0000
260.0000	1.0150	1.0172	0.8730	0.9337	0.4757	0.4648	3.0000
270.0000	1.0191	1.0197	0.8711	0.9328	0.4659	0.4550	3.0000
280.0000	1.0112	1.0185	0.8729	0.9333	0.4567	0.4449	3.0000
290.0000	1.0223	1.0197	0.8785	0.9356	0.4473	0.4350	3.0000
300.0000	1.0204	1.0209	0.8828	0.9371	0.4379	0.4254	3.0000
310.0000	1.0149	1.0221	0.8870	0.9381	0.4289	0.4168	3.0000
320.0000	1.0224	1.0234	0.8871	0.9383	0.4209	0.4102	3.0000
330.0000	1.0291	1.0246	0.8890	0.9390	0.4148	0.4037	3.0000
340.0000	1.0327	1.0283	0.8915	0.9392	0.4088	0.3975	3.0000
350.0000	1.0392	1.0344	0.8961	0.9403	0.4030	0.3917	3.0000
360.0000	1.0671	1.0689	0.9010	0.9419	0.3976	0.3854	3.0000
370.0000	1.1461	1.1624	0.9020	0.9421	0.3918	0.3797	3.0000
380.0000	1.2850	1.3346	0.9011	0.9417	0.3865	0.3746	3.0000
390.0000	1.4511	1.5264	0.9025	0.9424	0.3818	0.3693	3.0000
400.0000	1.5897	1.6740	0.9024	0.9426	0.3767	0.3635	3.0000
410.0000	1.7086	1.8081	0.9007	0.9424	0.3714	0.3591	3.0000
420.0000	1.8281	1.9569	0.9029	0.9433	0.3670	0.3540	3.0000
430.0000	1.9400	2.1009	0.9065	0.9449	0.3622	0.3488	3.0000
440.0000	2.0415	2.2399	0.9059	0.9446	0.3574	0.3450	3.0000
450.0000	2.1180	2.3739	0.9073	0.9455	0.3534	0.3405	3.0000
460.0000	2.2064	2.4748	0.9024	0.9446	0.3493	0.3360	3.0000
470.0000	2.2582	2.5535	0.9013	0.9449	0.3449	0.3325	3.0000
480.0000	2.3146	2.5756	0.8998	0.9446	0.3416	0.3295	3.0000

490.0000	2.3272	2.6125	0.9039	0.9464	0.3386	0.3260	3.0000
500.0000	2.3522	2.6175	0.9027	0.9464	0.3355	0.3212	3.0000
510.0000	2.3561	2.6212	0.8995	0.9462	0.3312	0.3157	3.0000
520.0000	2.3585	2.6310	0.8973	0.9460	0.3260	0.3158	3.0000
530.0000	2.3646	2.6322	0.8949	0.9458	0.3262	0.3138	3.0000
540.0000	2.3657	2.6421	0.8971	0.9471	0.3242	0.3102	3.0000
550.0000	2.3739	2.6482	0.8972	0.9478	0.3211	0.3079	3.0000
560.0000	2.3779	2.6654	0.8953	0.9476	0.3188	0.3051	3.0000
570.0000	2.3890	2.6704	0.8943	0.9476	0.3163	0.3026	3.0000
580.0000	2.3928	2.6581	0.8959	0.9485	0.3138	0.3002	3.0000
590.0000	2.3823	2.6544	0.8959	0.9492	0.3117	0.2971	3.0000
600.0000	2.3778	2.6605	0.8872	0.9469	0.3088	0.2945	3.0000
0.0000	1.0000	1.0000	1.0000	1.0000	1.0000	1.0000	4.0000
10.0000	1.0017	1.0038	1.0103	0.9400	0.9573	0.9658	4.0000
20.0000	1.0061	1.0089	1.0080	0.9329	0.9227	0.9383	4.0000
30.0000	1.0083	1.0115	1.0081	0.9302	0.9002	0.9205	4.0000
40.0000	1.0100	1.0140	1.0086	0.9297	0.8768	0.9036	4.0000
50.0000	1.0148	1.0204	1.0030	0.9264	0.8550	0.8884	4.0000
60.0000	1.0224	1.0230	1.0040	0.9252	0.8390	0.8768	4.0000
70.0000	1.0263	1.0268	0.9964	0.9201	0.8221	0.8647	4.0000
80.0000	1.0299	1.0306	0.9893	0.9172	0.8060	0.8526	4.0000
90.0000	1.0336	1.0332	0.9865	0.9143	0.7938	0.8438	4.0000
100.0000	1.0371	1.0370	0.9808	0.9109	0.7804	0.8336	4.0000
110.0000	1.0423	1.0421	0.9748	0.9075	0.7675	0.8233	4.0000
120.0000	1.0454	1.0446	0.9692	0.9029	0.7573	0.8148	4.0000
130.0000	1.0512	1.0497	0.9712	0.9152	0.7471	0.8063	4.0000
140.0000	1.0560	1.0548	0.9672	0.9125	0.7381	0.7985	4.0000
150.0000	1.0615	1.0599	0.9603	0.9102	0.7275	0.7886	4.0000
160.0000	1.0659	1.0625	0.9563	0.9098	0.7190	0.7805	4.0000
170.0000	1.0711	1.0663	0.9515	0.9046	0.7111	0.7726	4.0000
180.0000	1.0747	1.0714	0.9377	0.8912	0.7020	0.7630	4.0000
190.0000	1.0781	1.0753	0.9488	0.8558	0.6960	0.7568	4.0000
200.0000	1.0800	1.0765	0.9689	0.8397	0.6865	0.7469	4.0000
210.0000	1.0871	1.0880	0.9633	0.8303	0.6791	0.7387	4.0000
220.0000	1.1276	1.1518	0.9657	0.8301	0.6732	0.7323	4.0000
230.0000	1.2407	1.3291	0.9719	0.8319	0.6635	0.7219	4.0000
240.0000	1.4425	1.6327	0.9737	0.8359	0.6576	0.7153	4.0000
250.0000	1.6699	1.9694	0.9865	0.8431	0.6525	0.7096	4.0000
260.0000	1.6699	2.3304	0.9967	0.8489	0.6460	0.7023	4.0000
270.0000	1.8966	2.6939	1.0159	0.8554	0.6379	0.6931	4.0000
280.0000	2.1361	2.9974	1.0334	0.8608	0.6325	0.6869	4.0000
290.0000	2.3342	3.1633	1.0477	0.8663	0.6258	0.6792	4.0000
300.0000	2.4440	3.2423	1.0580	0.8708	0.6179	0.6706	4.0000
310.0000	2.4956	3.1441	1.0637	0.8735	0.6049	0.6576	4.0000
320.0000	2.4264	3.0089	1.0780	0.8787	0.5895	0.6420	4.0000
330.0000	2.3565	2.9554	1.0882	0.8823	0.5732	0.6245	4.0000
340.0000	2.3074	2.9260	1.0923	0.8845	0.5555	0.6054	4.0000
350.0000	2.2874	2.8954	1.1018	0.8883	0.5385	0.5870	4.0000
360.0000	2.2665	2.8788	1.1093	0.8916	0.5172	0.5634	4.0000
370.0000	2.2665	2.8597	1.1140	0.8941	0.5005	0.5447	4.0000
380.0000	2.2665	2.8418	1.1178	0.8963	0.4875	0.5302	4.0000
390.0000	2.2538	2.8202	1.1198	0.8977	0.4752	0.5163	4.0000
400.0000	2.2390	2.8112	1.1242	0.8997	0.4618	0.5011	4.0000
410.0000	2.2271	2.8010	1.1239	0.9008	0.4509	0.4883	4.0000
420.0000	2.2111	2.7717	1.1179	0.8999	0.4386	0.4733	4.0000
430.0000	2.2040	2.7500	1.1263	0.9029	0.4321	0.4657	4.0000
440.0000	2.1970	2.7334	1.1310	0.9049	0.4255	0.4579	4.0000
450.0000	2.1783	2.7334	1.1287	0.9049	0.4181	0.4489	4.0000

460.0000	2.1636	2.7232	1.1306	0.9062	0.4099	0.4387	4.0000
470.0000	2.1520	2.7054	1.1264	0.9058	0.4046	0.4323	4.0000
480.0000	2.1512	2.6926	1.1210	0.9051	0.3993	0.4255	4.0000
490.0000	2.1443	2.6862	1.1219	0.9064	0.3930	0.4178	4.0000
500.0000	2.1575	2.6747	1.1217	0.9076	0.3881	0.4120	4.0000
510.0000	2.1475	2.6684	1.1197	0.9073	0.3830	0.4058	4.0000
520.0000	2.1440	2.6582	1.1181	0.9080	0.3765	0.3978	4.0000
530.0000	2.1365	2.6518	1.1146	0.9080	0.3712	0.3915	4.0000
540.0000	2.1314	2.6403	1.1145	0.9087	0.3671	0.3866	4.0000
550.0000	2.1250	2.6390	1.1210	0.9113	0.3602	0.3785	4.0000
560.0000	2.1202	2.6288	1.1161	0.9113	0.3584	0.3763	4.0000
570.0000	2.1125	2.6199	1.1090	0.9093	0.3534	0.3704	4.0000
580.0000	2.1106	2.6212	1.1137	0.9113	0.3456	0.3606	4.0000
590.0000	2.1049	2.6224	1.1042	0.9095	0.3442	0.3591	4.0000
600.0000	2.0988	2.6071	1.0995	0.9087	0.3407	0.3548	4.0000
0.0000	1.0000	1.0000	1.0000	1.0000	1.0000	1.0000	5.0000
10.0000	1.0002	1.0012	1.0077	1.0123	0.9162	0.9067	5.0000
20.0000	0.9994	1.0025	1.0786	1.0167	0.8695	0.8643	5.0000
30.0000	0.9998	1.0025	1.0838	1.0202	0.8255	0.8290	5.0000
40.0000	0.9983	1.0025	1.0784	1.0277	0.7890	0.7986	5.0000
50.0000	0.9997	1.0050	1.0664	1.0309	0.7614	0.7751	5.0000
60.0000	1.0018	1.0062	1.0638	1.0346	0.7351	0.7523	5.0000
70.0000	1.0027	1.0075	1.0535	1.0356	0.7079	0.7295	5.0000
80.0000	1.0035	1.0100	1.0489	1.0383	0.6871	0.7115	5.0000
90.0000	1.0034	1.0100	1.0385	1.0414	0.6667	0.6938	5.0000
100.0000	1.0040	1.0112	1.0394	1.0463	0.6479	0.6768	5.0000
110.0000	1.0085	1.0150	1.0279	1.0482	0.6301	0.6601	5.0000
120.0000	1.0105	1.0162	1.0165	1.0479	0.6146	0.6453	5.0000
130.0000	1.0118	1.0187	1.0045	1.0490	0.5971	0.6281	5.0000
140.0000	1.0143	1.0200	0.9992	1.0485	0.5831	0.6148	5.0000
150.0000	1.0155	1.0225	0.9923	1.0483	0.5667	0.5986	5.0000
160.0000	1.0263	1.0250	0.9852	1.0480	0.5554	0.5869	5.0000
170.0000	1.0270	1.0262	0.9765	1.0485	0.5426	0.5733	5.0000
180.0000	1.0305	1.0287	0.9710	1.0485	0.5327	0.5628	5.0000
190.0000	1.0333	1.0312	0.9638	1.0488	0.5212	0.5503	5.0000
200.0000	1.0347	1.0337	0.9570	1.0491	0.5103	0.5384	5.0000
210.0000	1.0376	1.0362	0.9514	1.0490	0.5019	0.5290	5.0000
220.0000	1.0383	1.0400	0.9457	1.0479	0.4913	0.5166	5.0000
230.0000	1.0420	1.0449	0.9379	1.0436	0.4822	0.5061	5.0000
240.0000	1.0430	1.0474	0.9314	1.0384	0.4722	0.4947	5.0000
250.0000	1.0458	1.0524	0.9231	1.0359	0.4646	0.4863	5.0000
260.0000	1.0492	1.0574	0.9176	1.0345	0.4549	0.4758	5.0000
270.0000	1.0521	1.0599	0.9137	1.0357	0.4434	0.4632	5.0000
280.0000	1.0546	1.0649	0.9158	1.0390	0.4338	0.4526	5.0000
290.0000	1.0611	1.0737	0.9230	1.0419	0.4248	0.4424	5.0000
300.0000	1.0694	1.0874	0.9336	1.0444	0.4166	0.4330	5.0000
310.0000	1.0896	1.1199	0.9389	1.0490	0.4066	0.4218	5.0000
320.0000	1.1718	1.2347	0.9511	1.0526	0.3955	0.4097	5.0000
330.0000	1.3119	1.4557	0.9643	1.0263	0.3833	0.3964	5.0000
340.0000	1.4683	1.6916	0.9803	1.0293	0.3729	0.3850	5.0000
350.0000	1.6418	1.9526	0.9904	1.0315	0.3625	0.3738	5.0000
360.0000	1.8239	2.2297	0.9985	1.0338	0.3560	0.3668	5.0000
370.0000	2.0242	2.5518	1.0062	1.0357	0.3498	0.3597	5.0000
380.0000	2.2146	2.8714	1.0103	1.0368	0.3422	0.3513	5.0000
390.0000	2.3568	3.0811	1.0140	1.0381	0.3396	0.3485	5.0000
400.0000	2.4193	3.1860	1.0152	1.0398	0.3322	0.3404	5.0000
410.0000	2.4463	3.2447	1.0198	1.0409	0.3285	0.3362	5.0000
420.0000	2.4619	3.2797	1.0220	1.0417	0.3258	0.3333	5.0000

430.0000	2.4685	3.2921	1.0213	1.0425	0.3208	0.3278	5.0000
440.0000	2.4646	3.2859	1.0216	1.0431	0.3188	0.3255	5.0000
450.0000	2.4622	3.2821	1.0208	1.0436	0.3139	0.3198	5.0000
460.0000	2.4553	3.2722	1.0172	1.0438	0.3099	0.3155	5.0000
470.0000	2.4548	3.2697	1.0150	1.0438	0.3077	0.3129	5.0000
480.0000	2.4608	3.2846	1.0137	1.0441	0.3027	0.3074	5.0000
490.0000	2.4653	3.2934	1.0144	1.0450	0.2992	0.3036	5.0000
500.0000	2.4597	3.2859	1.0100	1.0447	0.2958	0.2997	5.0000
510.0000	2.4641	3.2971	1.0078	1.0446	0.2944	0.2986	5.0000
520.0000	2.4616	3.2946	1.0047	1.0444	0.2913	0.2954	5.0000
530.0000	2.4674	3.3084	1.0023	1.0447	0.2890	0.2928	5.0000
540.0000	2.4603	3.2971	0.9975	1.0441	0.2852	0.2892	5.0000
550.0000	2.4664	3.3096	0.9901	1.0428	0.2839	0.2881	5.0000
560.0000	2.4618	3.3059	0.9843	1.0420	0.2817	0.2867	5.0000
570.0000	2.4581	3.2996	0.9832	1.0423	0.2800	0.2863	5.0000
580.0000	2.4539	3.2971	0.9807	1.0420	0.2785	0.2857	5.0000
590.0000	2.4528	3.2984	0.9766	1.0416	0.2775	0.2852	5.0000
600.0000	2.4511	3.2971	0.9775	1.0422	0.2753	0.2829	5.0000
0.0000	1.0000	1.0000	1.0000	1.0000	1.0000	1.0000	6.0000
10.0000	0.9962	0.9964	0.9981	1.0049	0.9138	0.9347	6.0000
20.0000	0.9926	0.9964	1.0117	1.0107	0.8478	0.8778	6.0000
30.0000	0.9887	0.9964	1.0126	1.0110	0.7974	0.8308	6.0000
40.0000	0.9883	0.9976	1.0192	1.0195	0.7552	0.7910	6.0000
50.0000	0.9874	0.9988	1.0221	1.0321	0.7237	0.7610	6.0000
60.0000	0.9884	0.9988	1.0230	1.0420	0.6936	0.7338	6.0000
70.0000	0.9881	1.0000	1.0148	1.0482	0.6691	0.7110	6.0000
80.0000	0.9872	1.0012	1.0079	1.0526	0.6499	0.6925	6.0000
90.0000	0.9873	1.0024	0.9925	1.0547	0.6315	0.6737	6.0000
100.0000	0.9877	1.0036	0.9682	1.0543	0.6151	0.6568	6.0000
110.0000	0.9866	1.0048	0.9886	1.0573	0.6007	0.6415	6.0000
120.0000	0.9871	1.0072	0.9830	1.0615	0.5888	0.6285	6.0000
130.0000	0.9879	1.0096	0.9793	1.0640	0.5799	0.6184	6.0000
140.0000	0.9980	1.0108	0.9794	1.0679	0.5687	0.6058	6.0000
150.0000	0.9980	1.0120	0.9702	1.0688	0.5590	0.5944	6.0000
160.0000	0.9998	1.0156	0.9731	1.0699	0.5503	0.5847	6.0000
170.0000	0.9993	1.0180	0.9718	1.0707	0.5388	0.5713	6.0000
180.0000	0.9984	1.0192	0.9660	1.0717	0.5307	0.5621	6.0000
190.0000	0.9980	1.0216	0.9656	1.0711	0.5209	0.5508	6.0000
200.0000	0.9973	1.0240	0.9561	1.0696	0.5108	0.5389	6.0000
210.0000	1.0125	1.0251	0.9476	1.0685	0.5027	0.5297	6.0000
220.0000	1.0102	1.0275	0.9370	1.0679	0.4908	0.5156	6.0000
230.0000	1.0101	1.0287	0.9307	1.0669	0.4826	0.5062	6.0000
240.0000	1.0105	1.0299	0.9253	1.0652	0.4732	0.4953	6.0000
250.0000	1.0116	1.0335	0.9172	1.0633	0.4631	0.4835	6.0000
260.0000	1.0116	1.0335	0.9098	1.0625	0.4548	0.4737	6.0000
270.0000	1.0130	1.0359	0.9086	1.0591	0.4444	0.4617	6.0000
280.0000	1.0135	1.0371	0.9013	1.0594	0.4379	0.4543	6.0000
290.0000	1.0150	1.0395	0.8985	1.0630	0.4301	0.4454	6.0000
300.0000	1.0148	1.0383	0.8996	1.0676	0.4216	0.4355	6.0000
310.0000	1.0162	1.0419	0.9022	1.0717	0.4134	0.4259	6.0000
320.0000	1.0175	1.0431	0.9070	1.0743	0.4070	0.4187	6.0000
330.0000	1.0190	1.0455	0.9102	1.0779	0.4001	0.4106	6.0000
340.0000	1.0206	1.0479	0.9141	1.0799	0.3952	0.4045	6.0000
350.0000	1.0234	1.0527	0.9156	1.0837	0.3898	0.3976	6.0000
360.0000	1.0313	1.0659	0.9224	1.0856	0.3859	0.3929	6.0000
370.0000	1.0588	1.1102	0.9268	1.0877	0.3835	0.3898	6.0000
380.0000	1.1335	1.2204	0.9281	1.0906	0.3799	0.3854	6.0000
390.0000	1.2579	1.3856	0.9313	1.0924	0.3775	0.3822	6.0000

400.0000	1.3995	1.5629	0.9329	1.0948	0.3740	0.3779	6.0000
410.0000	1.5427	1.7329	0.9369	1.0977	0.3723	0.3757	6.0000
420.0000	1.6822	1.8994	0.9408	1.1003	0.3682	0.3708	6.0000
430.0000	1.8367	2.0790	0.9434	1.1032	0.3656	0.3673	6.0000
440.0000	1.9934	2.2659	0.9485	1.1035	0.3645	0.3658	6.0000
450.0000	2.1533	2.4551	0.9475	1.0986	0.3607	0.3612	6.0000
460.0000	2.2915	2.6144	0.9481	1.0905	0.3580	0.3577	6.0000
470.0000	2.3808	2.7210	0.9596	1.0908	0.3574	0.3569	6.0000
480.0000	2.4345	2.7832	0.9576	1.0916	0.3554	0.3544	6.0000
490.0000	2.4635	2.8192	0.9561	1.0943	0.3530	0.3514	6.0000
500.0000	2.4829	2.8443	0.9634	1.0956	0.3487	0.3462	6.0000
510.0000	2.4946	2.8599	0.9631	1.0957	0.3466	0.3438	6.0000
520.0000	2.5031	2.8707	0.9611	1.0956	0.3447	0.3414	6.0000
530.0000	2.5067	2.8766	0.9568	1.0977	0.3422	0.3385	6.0000
540.0000	2.5091	2.8802	0.9618	1.0976	0.3391	0.3350	6.0000
550.0000	2.5155	2.8898	0.9573	1.0983	0.3374	0.3329	6.0000
560.0000	2.5188	2.8958	0.9555	1.1000	0.3327	0.3273	6.0000
570.0000	2.5234	2.9030	0.9592	1.1009	0.3305	0.3248	6.0000
580.0000	2.5233	2.9018	0.9596	1.1015	0.3295	0.3237	6.0000
590.0000	2.5254	2.9066	0.9610	1.1015	0.3266	0.3204	6.0000
600.0000	2.5336	2.9102	0.9576	1.1022	0.3243	0.3176	6.0000
0.0000	1.0000	1.0000	1.0000	1.0000	1.0000	1.0000	7.0000
10.0000	1.0015	1.0000	0.9175	0.9339	0.9541	0.9740	7.0000
20.0000	1.0023	1.0013	0.8861	0.9053	0.9104	0.9420	7.0000
30.0000	1.0046	1.0051	0.8766	0.8910	0.8727	0.9154	7.0000
40.0000	1.0059	1.0088	0.8745	0.8820	0.8448	0.8952	7.0000
50.0000	1.0042	1.0101	0.8778	0.8771	0.8216	0.8790	7.0000
60.0000	1.0054	1.0114	0.8739	0.8711	0.8024	0.8655	7.0000
70.0000	1.0091	1.0152	0.8521	0.8485	0.7856	0.8536	7.0000
80.0000	1.0122	1.0177	0.8496	0.8485	0.7708	0.8434	7.0000
90.0000	1.0205	1.0227	0.8448	0.8458	0.7570	0.8336	7.0000
100.0000	1.0243	1.0265	0.8456	0.8453	0.7449	0.8254	7.0000
110.0000	1.0279	1.0303	0.8429	0.8405	0.7322	0.8164	7.0000
120.0000	1.0334	1.0366	0.8375	0.8356	0.7204	0.8074	7.0000
130.0000	1.0416	1.0404	0.8333	0.8300	0.7077	0.7974	7.0000
140.0000	1.0465	1.0467	0.8342	0.8266	0.6956	0.7876	7.0000
150.0000	1.0518	1.0530	0.8281	0.8213	0.6886	0.7827	7.0000
160.0000	1.0567	1.0593	0.8144	0.8167	0.6775	0.7746	7.0000
170.0000	1.0631	1.0669	0.8050	0.8111	0.6768	0.7807	7.0000
180.0000	1.0679	1.0770	0.7945	0.8116	0.6648	0.7718	7.0000
190.0000	1.0759	1.0972	0.7865	0.8009	0.6482	0.7542	7.0000
200.0000	1.0909	1.2096	0.7779	0.7810	0.6323	0.7420	7.0000
210.0000	1.1614	1.4495	0.7684	0.7751	0.6241	0.7365	7.0000
220.0000	1.3158	1.6578	0.7648	0.7710	0.6140	0.7291	7.0000
230.0000	1.4571	1.8106	0.7695	0.7788	0.6088	0.7266	7.0000
240.0000	1.5707	1.8788	0.7806	0.7844	0.5981	0.7164	7.0000
250.0000	1.6269	1.8990	0.7941	0.7904	0.5902	0.7080	7.0000
260.0000	1.6490	1.9331	0.8151	0.8011	0.5808	0.6984	7.0000
270.0000	1.6805	2.0114	0.8282	0.7975	0.5607	0.6755	7.0000
280.0000	1.7438	2.0909	0.8498	0.8016	0.5588	0.6738	7.0000
290.0000	1.8010	2.1742	0.8648	0.8023	0.5539	0.6682	7.0000
300.0000	1.8690	2.2626	0.8819	0.8060	0.5449	0.6577	7.0000
310.0000	1.9332	2.3510	0.8882	0.8038	0.5339	0.6449	7.0000
320.0000	1.9934	2.5101	0.9062	0.8062	0.5232	0.6328	7.0000
330.0000	2.1105	2.6793	0.9144	0.8065	0.5111	0.6187	7.0000
340.0000	2.2395	2.7790	0.9227	0.8060	0.4998	0.6055	7.0000
350.0000	2.3148	2.8144	0.9302	0.8065	0.4883	0.5921	7.0000
360.0000	2.3378	2.8144	0.9392	0.8069	0.4762	0.5776	7.0000

370.0000	2.3362	2.8295	0.9368	0.8050	0.4642	0.5629	7.0000
380.0000	2.3444	2.8295	0.9464	0.8018	0.4515	0.5470	7.0000
390.0000	2.3432	2.8030	0.9516	0.7963	0.4415	0.5340	7.0000
400.0000	2.3261	2.8043	0.9525	0.7885	0.4330	0.5231	7.0000
410.0000	2.3265	2.8043	0.9491	0.7875	0.4230	0.5100	7.0000
420.0000	2.3265	2.7992	0.9481	0.7882	0.4152	0.4999	7.0000
430.0000	2.3229	2.7917	0.9472	0.7885	0.4068	0.4888	7.0000
440.0000	2.3282	2.8068	0.9547	0.7916	0.3963	0.4744	7.0000
450.0000	2.3307	2.8081	0.9460	0.7892	0.3878	0.4630	7.0000
460.0000	2.3215	2.7955	0.9533	0.7919	0.3824	0.4555	7.0000
470.0000	2.3125	2.7803	0.9461	0.7887	0.3774	0.4488	7.0000
480.0000	2.3043	2.7677	0.9449	0.7870	0.3707	0.4395	7.0000
490.0000	2.2838	2.7361	0.9352	0.7831	0.3652	0.4320	7.0000
500.0000	2.2790	2.7298	0.9305	0.7810	0.3596	0.4245	7.0000
510.0000	2.2797	2.7311	0.9368	0.7797	0.3529	0.4151	7.0000
520.0000	2.2604	2.7020	0.9302	0.7773	0.3487	0.4092	7.0000
530.0000	2.2545	2.6932	0.9246	0.7645	0.3431	0.4015	7.0000
540.0000	2.2380	2.6692	0.9200	0.7588	0.3377	0.3941	7.0000
550.0000	2.2328	2.6641	0.9168	0.7497	0.3316	0.3857	7.0000
560.0000	2.2363	2.6692	0.9137	0.7406	0.3268	0.3791	7.0000
570.0000	2.2341	2.6667	0.9086	0.7335	0.3214	0.3716	7.0000
580.0000	2.2205	2.6490	0.9023	0.7258	0.3168	0.3655	7.0000
590.0000	2.2187	2.6477	0.9003	0.7263	0.3103	0.3567	7.0000
600.0000	2.2067	2.6301	0.8972	0.7154	0.3065	0.3516	7.0000
0.0000	1.0000	1.0000	1.0000	1.0000	1.0000	1.0000	8.0000
10.0000	1.0012	1.0012	0.9996	0.9924	0.9474	0.9614	8.0000
20.0000	1.0021	1.0012	1.0001	0.9924	0.9082	0.9329	8.0000
30.0000	0.9981	1.0012	0.9970	0.9894	0.8736	0.9084	8.0000
40.0000	0.9940	1.0025	1.0060	0.9911	0.8487	0.8903	8.0000
50.0000	1.0064	1.0037	1.0016	0.9882	0.8233	0.8725	8.0000
60.0000	1.0066	1.0061	0.9875	0.9884	0.8028	0.8572	8.0000
70.0000	1.0064	1.0074	0.9925	0.9893	0.7841	0.8430	8.0000
80.0000	1.0084	1.0110	1.0004	0.9923	0.7662	0.8296	8.0000
90.0000	1.0124	1.0147	0.9965	0.9973	0.7498	0.8172	8.0000
100.0000	1.0157	1.0172	0.9867	1.0014	0.7371	0.8074	8.0000
110.0000	1.0186	1.0208	0.9655	1.0047	0.7226	0.7962	8.0000
120.0000	1.0217	1.0257	0.9672	1.0133	0.7090	0.7855	8.0000
130.0000	1.0249	1.0294	0.9712	1.0260	0.6956	0.7745	8.0000
140.0000	1.0282	1.0355	0.9501	1.0251	0.6846	0.7654	8.0000
150.0000	1.0330	1.0417	0.9457	1.0370	0.6725	0.7551	8.0000
160.0000	1.0370	1.0466	0.9511	1.0529	0.6694	0.7650	8.0000
170.0000	1.0405	1.0527	0.9378	1.0575	0.6434	0.7196	8.0000
180.0000	1.0448	1.0613	0.9272	1.0578	0.6331	0.7100	8.0000
190.0000	1.0510	1.0686	0.9220	1.0592	0.6223	0.7011	8.0000
200.0000	1.0557	1.0760	0.9217	1.0294	0.6150	0.6945	8.0000
210.0000	1.0609	1.0870	0.9144	1.0287	0.6064	0.6862	8.0000
220.0000	1.0688	1.1054	0.9056	1.0254	0.5970	0.6771	8.0000
230.0000	1.0813	1.1544	0.8920	1.0171	0.5877	0.6680	8.0000
240.0000	1.1147	1.3027	0.8954	0.9169	0.5809	0.6613	8.0000
250.0000	1.2193	1.5748	0.8926	0.9121	0.5727	0.6529	8.0000
260.0000	1.4139	1.8100	0.8890	0.9124	0.5654	0.6451	8.0000
270.0000	1.6713	1.9375	0.8957	0.9160	0.5570	0.6364	8.0000
280.0000	1.7168	2.0061	0.9038	0.9201	0.5507	0.6297	8.0000
290.0000	1.7333	2.0343	0.9089	0.9227	0.5443	0.6230	8.0000
300.0000	1.7466	2.0576	0.9151	0.9256	0.5364	0.6143	8.0000
310.0000	1.7572	2.0760	0.9249	0.9287	0.5298	0.6074	8.0000
320.0000	1.7669	2.0956	0.9315	0.9314	0.5231	0.6004	8.0000
330.0000	1.7845	2.1275	0.9410	0.9343	0.5149	0.5911	8.0000

340.0000	1.8252	2.2010	0.9459	0.9363	0.5072	0.5822	8.0000
350.0000	1.8848	2.3113	0.9515	0.9384	0.5005	0.5747	8.0000
360.0000	1.9903	2.4498	0.9501	0.9388	0.4946	0.5680	8.0000
370.0000	2.0882	2.5723	0.9531	0.9402	0.4870	0.5592	8.0000
380.0000	2.1462	2.6532	0.9521	0.9408	0.4776	0.5480	8.0000
390.0000	2.1902	2.7181	0.9526	0.9413	0.4719	0.5413	8.0000
400.0000	2.2243	2.7647	0.9594	0.9437	0.4638	0.5317	8.0000
410.0000	2.2338	2.7770	0.9603	0.9444	0.4570	0.5234	8.0000
420.0000	2.2357	2.7819	0.9652	0.9461	0.4514	0.5167	8.0000
430.0000	2.2381	2.7855	0.9676	0.9470	0.4439	0.5075	8.0000
440.0000	2.2357	2.7831	0.9672	0.9476	0.4376	0.5000	8.0000
450.0000	2.2199	2.7635	0.9709	0.9491	0.4307	0.4913	8.0000
460.0000	2.2284	2.7745	0.9722	0.9499	0.4235	0.4825	8.0000
470.0000	2.2325	2.7806	0.9742	0.9508	0.4173	0.4747	8.0000
480.0000	2.2230	2.7708	0.9722	0.9509	0.4120	0.4682	8.0000
490.0000	2.2117	2.7525	0.9658	0.9499	0.4050	0.4593	8.0000
500.0000	2.2257	2.7733	0.9688	0.9512	0.3977	0.4501	8.0000
510.0000	2.2190	2.7647	0.9652	0.9509	0.3907	0.4413	8.0000
520.0000	2.2178	2.7623	0.9624	0.9508	0.3869	0.4366	8.0000
530.0000	2.2238	2.7708	0.9606	0.9511	0.3792	0.4266	8.0000
540.0000	2.2050	2.7451	0.9576	0.9508	0.3734	0.4193	8.0000
550.0000	2.2041	2.7439	0.9554	0.9508	0.3680	0.4125	8.0000
560.0000	2.2140	2.7574	0.9540	0.9511	0.3637	0.4068	8.0000
570.0000	2.2085	2.7488	0.9512	0.9509	0.3586	0.4005	8.0000
580.0000	2.2104	2.7525	0.9485	0.9503	0.3536	0.3941	8.0000
590.0000	2.2113	2.7549	0.9423	0.9491	0.3485	0.3878	8.0000
600.0000	2.1893	2.7230	0.9426	0.9497	0.3429	0.3807	8.0000
0.0000	1.0000	1.0000	1.0000	1.0000	1.0000		9.0000
10.0000	1.0001	1.0024	0.9815	0.9836	0.9774	0.9789	9.0000
20.0000	0.9991	1.0000	0.9787	0.9803	0.9537	0.9594	9.0000
30.0000	0.9992	0.9976	0.9634	0.9774	0.9347	0.9442	9.0000
40.0000	0.9996	0.9988	0.9674	0.9777	0.9160	0.9285	9.0000
50.0000	0.9992	0.9976	0.9693	0.9777	0.8984	0.9145	9.0000
60.0000	1.0013	0.9988	0.9668	0.9769	0.8845	0.9056	9.0000
70.0000	1.0002	1.0000	0.9651	0.9761	0.8729	0.8990	9.0000
80.0000	0.9963	0.9988	0.9574	0.9740	0.8606	0.8915	9.0000
90.0000	0.9971	1.0024	0.9532	0.9728	0.8482	0.8842	9.0000
100.0000	0.9972	1.0012	0.9489	0.9718	0.8446	0.8786	9.0000
110.0000	0.9983	1.0036	0.9385	0.9695	0.8358	0.8735	9.0000
120.0000	0.9985	1.0048	0.9430	0.9701	0.8258	0.8676	9.0000
130.0000	0.9998	1.0060	0.9235	0.9666	0.8166	0.8568	9.0000
140.0000	1.0023	1.0084	0.9207	0.9661	0.8034	0.8503	9.0000
150.0000	1.0044	1.0109	0.9193	0.9660	0.7956	0.8427	9.0000
160.0000	1.0034	1.0084	0.9149	0.9651	0.7864	0.8360	9.0000
170.0000	1.0032	1.0097	0.9098	0.9642	0.7785	0.8296	9.0000
180.0000	1.0036	1.0109	0.9041	0.9632	0.7707	0.8219	9.0000
190.0000	1.0048	1.0121	0.9016	0.9626	0.7619	0.8148	9.0000
200.0000	1.0053	1.0133	0.8979	0.9617	0.7536	0.8088	9.0000
210.0000	1.0051	1.0121	0.8950	0.9611	0.7469	0.8025	9.0000
220.0000	1.0052	1.0133	0.8883	0.9598	0.7397	0.7959	9.0000
230.0000	1.0075	1.0145	0.8812	0.9583	0.7324	0.7924	9.0000
240.0000	1.0081	1.0169	0.8700	0.9551	0.7284	0.7862	9.0000
250.0000	1.0085	1.0181	0.8643	0.9512	0.7210	0.7809	9.0000
260.0000	1.0110	1.0205	0.8557	0.9454	0.7146	0.7725	9.0000
270.0000	1.0129	1.0229	0.8481	0.9405	0.7059	0.7640	9.0000
280.0000	1.0141	1.0241	0.8454	0.9393	0.6968	0.7553	9.0000
290.0000	1.0153	1.0265	0.8410	0.9379	0.6889	0.7506	9.0000
300.0000	1.0166	1.0277	0.8439	0.9394	0.6835	0.7431	9.0000

310.0000	1.0179	1.0302	0.8483	0.9417	0.6765	0.7359	9.0000
320.0000	1.0211	1.0338	0.8478	0.9417	0.6693	0.7307	9.0000
330.0000	1.0304	1.0470	0.8556	0.9444	0.6642	0.7270	9.0000
340.0000	1.0544	1.0820	0.8542	0.9441	0.6598	0.7190	9.0000
350.0000	1.1577	1.2256	0.8541	0.9441	0.6520	0.7118	9.0000
360.0000	1.4090	1.5476	0.8608	0.9630	0.6449	0.7071	9.0000
370.0000	1.6596	1.8287	0.8603	0.9549	0.6399	0.7035	9.0000
380.0000	1.8376	2.0338	0.8571	0.9506	0.6345	0.6998	9.0000
390.0000	1.9674	2.1990	0.8564	0.9485	0.6309	0.6925	9.0000
400.0000	2.0956	2.3788	0.8544	0.9477	0.6238	0.6892	9.0000
410.0000	2.1870	2.5042	0.8549	0.9491	0.6206	0.6839	9.0000
420.0000	2.2313	2.5573	0.8563	0.9502	0.6152	0.6797	9.0000
430.0000	2.2534	2.5790	0.8532	0.9497	0.6112	0.6731	9.0000
440.0000	2.2693	2.5959	0.8466	0.9488	0.6049	0.6660	9.0000
450.0000	2.2752	2.6019	0.8412	0.9492	0.5981	0.6585	9.0000
460.0000	2.2769	2.6019	0.8340	0.9488	0.5909	0.6534	9.0000
470.0000	2.2819	2.6068	0.8306	0.9487	0.5863	0.6490	9.0000
480.0000	2.2869	2.6116	0.8317	0.9490	0.5821	0.6445	9.0000
490.0000	2.2878	2.6116	0.8251	0.9428	0.5781	0.6398	9.0000
500.0000	2.2849	2.6068	0.8270	0.9439	0.5734	0.6356	9.0000
510.0000	2.2829	2.6031	0.8255	0.9432	0.5645	0.6300	9.0000
520.0000	2.2880	2.6104	0.8255	0.9432	0.5590	0.6243	9.0000
530.0000	2.2802	2.5995	0.8247	0.9428	0.5555	0.6208	9.0000
540.0000	2.2828	2.6019	0.8251	0.9431	0.5526	0.6173	9.0000
550.0000	2.2904	2.6116	0.8249	0.9431	0.5449	0.6095	9.0000
560.0000	2.2904	2.6104	0.8251	0.9432	0.5425	0.6067	9.0000
570.0000	2.2944	2.6152	0.8254	0.9434	0.5355	0.5997	9.0000
580.0000	2.2891	2.6080	0.8262	0.9441	0.5397	0.6040	9.0000
590.0000	2.2943	2.6128	0.8266	0.9444	0.5341	0.5979	9.0000
600.0000	2.2958	2.6152	0.8255	0.9438	0.5266	0.5897	9.0000

Sheet 3

	Single Metal-NNs-M			Single Metal-RF-C	
No.	Ground	Predicted	No.	Ground	Predicted
1.00000	29.00000	42.84140	1.00000	0.00100	0.00054
4.00000	29.00000	29.98189	4.00000	0.00100	0.00069
8.00000	29.00000	25.56201	8.00000	0.00100	0.00077
9.00000	29.00000	29.49698	9.00000	0.00100	0.00078
12.00000	29.00000	27.57243	12.00000	0.00100	0.00090
17.00000	29.00000	32.87847	17.00000	0.00100	0.00090
20.00000	29.00000	30.08975	20.00000	0.00100	0.00076
22.00000	29.00000	30.22950	22.00000	0.00100	0.00083
26.00000	29.00000	29.44440	26.00000	0.00100	0.00099
32.00000	29.00000	29.43095	32.00000	0.00100	0.00099
33.00000	29.00000	29.42923	33.00000	0.00100	0.00099
35.00000	29.00000	29.43144	35.00000	0.00100	0.00100
36.00000	29.00000	29.48377	36.00000	0.00100	0.00100
38.00000	29.00000	30.18388	38.00000	0.00100	0.00100
42.00000	29.00000	28.27731	42.00000	0.00100	0.00100
45.00000	29.00000	28.28756	45.00000	0.00100	0.00100
52.00000	29.00000	28.28766	52.00000	0.00100	0.00100
54.00000	29.00000	28.28766	54.00000	0.00100	0.00100
59.00000	29.00000	28.28766	59.00000	0.00100	0.00100
62.00000	29.00000	42.84140	62.00000	0.00050	0.00054
70.00000	29.00000	30.15350	70.00000	0.00050	0.00050
75.00000	29.00000	26.49952	75.00000	0.00050	0.00056
78.00000	29.00000	26.51540	78.00000	0.00050	0.00053
79.00000	29.00000	26.65834	79.00000	0.00050	0.00055
81.00000	29.00000	26.68742	81.00000	0.00050	0.00061
82.00000	29.00000	26.68880	82.00000	0.00050	0.00057
95.00000	29.00000	26.69082	95.00000	0.00050	0.00052
101.00000	29.00000	26.70806	101.00000	0.00050	0.00052
104.00000	29.00000	26.74080	104.00000	0.00050	0.00055
110.00000	29.00000	28.25851	110.00000	0.00050	0.00053
111.00000	29.00000	26.89692	111.00000	0.00050	0.00052
114.00000	29.00000	26.94028	114.00000	0.00050	0.00050
115.00000	29.00000	27.12580	115.00000	0.00050	0.00052
116.00000	29.00000	26.71837	116.00000	0.00050	0.00050
119.00000	29.00000	26.69902	119.00000	0.00050	0.00050
123.00000	29.00000	42.84140	123.00000	0.00020	0.00054
125.00000	29.00000	37.64261	125.00000	0.00020	0.00054
127.00000	29.00000	32.73444	127.00000	0.00020	0.00051
131.00000	29.00000	35.76719	131.00000	0.00020	0.00031
134.00000	29.00000	31.05032	134.00000	0.00020	0.00032
135.00000	29.00000	28.55047	135.00000	0.00020	0.00036
137.00000	29.00000	27.89031	137.00000	0.00020	0.00030
138.00000	29.00000	21.24794	138.00000	0.00020	0.00031
143.00000	29.00000	26.62082	143.00000	0.00020	0.00035
152.00000	29.00000	30.62974	152.00000	0.00020	0.00024
155.00000	29.00000	36.06150	155.00000	0.00020	0.00021
157.00000	29.00000	18.80192	157.00000	0.00020	0.00029
162.00000	29.00000	31.37653	162.00000	0.00020	0.00022
166.00000	29.00000	30.29795	166.00000	0.00020	0.00024
167.00000	29.00000	29.28108	167.00000	0.00020	0.00026
175.00000	29.00000	30.68128	175.00000	0.00020	0.00023
177.00000	29.00000	30.61930	177.00000	0.00020	0.00022
179.00000	29.00000	28.19783	179.00000	0.00020	0.00022
183.00000	29.00000	30.70493	183.00000	0.00020	0.00023
184.00000	80.00000	42.84140	184.00000	0.00100	0.00054

185.00000	80.00000	51.47577	185.00000	0.00100	0.00051
189.00000	80.00000	61.51493	189.00000	0.00100	0.00044
190.00000	80.00000	61.44330	190.00000	0.00100	0.00038
191.00000	80.00000	66.45428	191.00000	0.00100	0.00039
192.00000	80.00000	79.25137	192.00000	0.00100	0.00040
193.00000	80.00000	79.45828	193.00000	0.00100	0.00052
197.00000	80.00000	78.25723	197.00000	0.00100	0.00081
200.00000	80.00000	76.30439	200.00000	0.00100	0.00075
201.00000	80.00000	74.74279	201.00000	0.00100	0.00075
203.00000	80.00000	74.75868	203.00000	0.00100	0.00073
204.00000	80.00000	73.69220	204.00000	0.00100	0.00076
206.00000	80.00000	67.13977	206.00000	0.00100	0.00069
207.00000	80.00000	54.52944	207.00000	0.00100	0.00064
208.00000	80.00000	91.13848	208.00000	0.00100	0.00076
209.00000	80.00000	87.82464	209.00000	0.00100	0.00076
213.00000	80.00000	80.50086	213.00000	0.00100	0.00100
220.00000	80.00000	80.47148	220.00000	0.00100	0.00100
221.00000	80.00000	80.47022	221.00000	0.00100	0.00100
227.00000	80.00000	80.46944	227.00000	0.00100	0.00100
231.00000	80.00000	80.46942	231.00000	0.00100	0.00100
236.00000	80.00000	81.64636	236.00000	0.00100	0.00100
237.00000	80.00000	93.79181	237.00000	0.00100	0.00100
241.00000	80.00000	78.15757	241.00000	0.00100	0.00100
248.00000	80.00000	36.06833	248.00000	0.00050	0.00047
251.00000	80.00000	90.08410	251.00000	0.00050	0.00053
259.00000	80.00000	73.58167	259.00000	0.00050	0.00053
261.00000	80.00000	87.90233	261.00000	0.00050	0.00052
264.00000	80.00000	79.39828	264.00000	0.00050	0.00050
266.00000	80.00000	80.38425	266.00000	0.00050	0.00055
272.00000	80.00000	78.96934	272.00000	0.00050	0.00070
276.00000	80.00000	80.46993	276.00000	0.00050	0.00049
281.00000	80.00000	80.46912	281.00000	0.00050	0.00050
294.00000	80.00000	80.40154	294.00000	0.00050	0.00050
296.00000	80.00000	80.32965	296.00000	0.00050	0.00050
298.00000	80.00000	80.18616	298.00000	0.00050	0.00050
300.00000	80.00000	79.78739	300.00000	0.00050	0.00050
308.00000	80.00000	72.88597	308.00000	0.00020	0.00044
310.00000	80.00000	67.53078	310.00000	0.00020	0.00038
315.00000	80.00000	77.68824	315.00000	0.00020	0.00040
316.00000	80.00000	74.97878	316.00000	0.00020	0.00045
317.00000	80.00000	78.49582	317.00000	0.00020	0.00047
318.00000	80.00000	79.46582	318.00000	0.00020	0.00047
319.00000	80.00000	78.51306	319.00000	0.00020	0.00044
322.00000	80.00000	82.30871	322.00000	0.00020	0.00030
325.00000	80.00000	79.90208	325.00000	0.00020	0.00034
326.00000	80.00000	101.49964	326.00000	0.00020	0.00039
328.00000	80.00000	75.45655	328.00000	0.00020	0.00033
330.00000	80.00000	63.25335	330.00000	0.00020	0.00038
332.00000	80.00000	61.45807	332.00000	0.00020	0.00063
334.00000	80.00000	90.89663	334.00000	0.00020	0.00025
339.00000	80.00000	46.50862	339.00000	0.00020	0.00027
344.00000	80.00000	88.37362	344.00000	0.00020	0.00045
350.00000	80.00000	80.46919	350.00000	0.00020	0.00021
351.00000	80.00000	80.46848	351.00000	0.00020	0.00021
353.00000	80.00000	80.44797	353.00000	0.00020	0.00021
354.00000	80.00000	80.45559	354.00000	0.00020	0.00021
357.00000	80.00000	80.46269	357.00000	0.00020	0.00020
358.00000	80.00000	80.46428	358.00000	0.00020	0.00021

360.00000	80.00000	80.46533	360.00000	0.00020	0.00021
366.00000	80.00000	76.90220	366.00000	0.00020	0.00034
367.00000	13.00000	42.84140	367.00000	0.00100	0.00054
371.00000	13.00000	12.23429	371.00000	0.00100	0.00076
373.00000	13.00000	5.21464	373.00000	0.00100	0.00069
377.00000	13.00000	9.01863	377.00000	0.00100	0.00092
383.00000	13.00000	15.13774	383.00000	0.00100	0.00093
390.00000	13.00000	7.13444	390.00000	0.00100	0.00095
391.00000	13.00000	6.50488	391.00000	0.00100	0.00095
392.00000	13.00000	8.28835	392.00000	0.00100	0.00098
393.00000	13.00000	10.99550	393.00000	0.00100	0.00100
395.00000	13.00000	6.88889	395.00000	0.00100	0.00100
401.00000	13.00000	7.50844	401.00000	0.00100	0.00100
403.00000	13.00000	9.90561	403.00000	0.00100	0.00100
408.00000	13.00000	14.18891	408.00000	0.00100	0.00100
412.00000	13.00000	12.56441	412.00000	0.00100	0.00100
417.00000	13.00000	26.92168	417.00000	0.00100	0.00100
420.00000	13.00000	11.99973	420.00000	0.00100	0.00100
422.00000	13.00000	8.40780	422.00000	0.00100	0.00100
424.00000	13.00000	8.88290	424.00000	0.00100	0.00099
427.00000	13.00000	8.61820	427.00000	0.00100	0.00092
428.00000	13.00000	42.84140	428.00000	0.00050	0.00054
429.00000	13.00000	33.70873	429.00000	0.00050	0.00059
434.00000	13.00000	16.40473	434.00000	0.00050	0.00050
440.00000	13.00000	15.34761	440.00000	0.00050	0.00048
445.00000	13.00000	17.18460	445.00000	0.00050	0.00061
446.00000	13.00000	14.36521	446.00000	0.00050	0.00057
452.00000	13.00000	8.46596	452.00000	0.00050	0.00049
454.00000	13.00000	1.26532	454.00000	0.00050	0.00051
456.00000	13.00000	22.46290	456.00000	0.00050	0.00050
459.00000	13.00000	22.62050	459.00000	0.00050	0.00049
460.00000	13.00000	22.34853	460.00000	0.00050	0.00051
465.00000	13.00000	26.13664	465.00000	0.00050	0.00050
473.00000	13.00000	17.04842	473.00000	0.00050	0.00050
483.00000	13.00000	12.67396	483.00000	0.00050	0.00053
486.00000	13.00000	5.59339	486.00000	0.00050	0.00059
489.00000	13.00000	42.84140	489.00000	0.00020	0.00054
493.00000	13.00000	8.15449	493.00000	0.00020	0.00031
494.00000	13.00000	8.17404	494.00000	0.00020	0.00033
497.00000	13.00000	11.37356	497.00000	0.00020	0.00026
501.00000	13.00000	7.30670	501.00000	0.00020	0.00026
505.00000	13.00000	15.32351	505.00000	0.00020	0.00029
506.00000	13.00000	15.86024	506.00000	0.00020	0.00030
509.00000	13.00000	7.83801	509.00000	0.00020	0.00031
511.00000	13.00000	10.11897	511.00000	0.00020	0.00026
517.00000	13.00000	17.41597	517.00000	0.00020	0.00022
519.00000	13.00000	16.66643	519.00000	0.00020	0.00021
523.00000	13.00000	24.24440	523.00000	0.00020	0.00022
526.00000	13.00000	11.19592	526.00000	0.00020	0.00021
527.00000	13.00000	6.60975	527.00000	0.00020	0.00021
533.00000	13.00000	7.51178	533.00000	0.00020	0.00022
536.00000	13.00000	10.69733	536.00000	0.00020	0.00021
537.00000	13.00000	12.14587	537.00000	0.00020	0.00022
539.00000	13.00000	11.97499	539.00000	0.00020	0.00021
543.00000	13.00000	13.77374	543.00000	0.00020	0.00020
544.00000	13.00000	16.15575	544.00000	0.00020	0.00024
548.00000	13.00000	13.10056	548.00000	0.00020	0.00021
550.00000	24.00000	42.84140	550.00000	0.00100	0.00054

552.00000	24.00000	26.81972	552.00000	0.00100	0.00084
554.00000	24.00000	26.71138	554.00000	0.00100	0.00082
560.00000	24.00000	26.43310	560.00000	0.00100	0.00090
567.00000	24.00000	24.67067	567.00000	0.00100	0.00094
569.00000	24.00000	25.68218	569.00000	0.00100	0.00093
571.00000	24.00000	25.51246	571.00000	0.00100	0.00089
573.00000	24.00000	24.33052	573.00000	0.00100	0.00087
574.00000	24.00000	24.67177	574.00000	0.00100	0.00086
579.00000	24.00000	21.91727	579.00000	0.00100	0.00096
580.00000	24.00000	13.69419	580.00000	0.00100	0.00095
583.00000	24.00000	21.16498	583.00000	0.00100	0.00093
585.00000	24.00000	28.58915	585.00000	0.00100	0.00096
591.00000	24.00000	24.40284	591.00000	0.00100	0.00094
595.00000	24.00000	28.03851	595.00000	0.00100	0.00083
596.00000	24.00000	28.09253	596.00000	0.00100	0.00083
603.00000	24.00000	26.78548	603.00000	0.00100	0.00100
608.00000	24.00000	22.35694	608.00000	0.00100	0.00099
610.00000	24.00000	27.45856	610.00000	0.00100	0.00097
611.00000	24.00000	42.84140	611.00000	0.00050	0.00054
613.00000	24.00000	44.12291	613.00000	0.00050	0.00048
619.00000	24.00000	27.55350	619.00000	0.00050	0.00050
623.00000	24.00000	28.00688	623.00000	0.00050	0.00049
625.00000	24.00000	27.77746	625.00000	0.00050	0.00049
634.00000	24.00000	22.96257	634.00000	0.00050	0.00048
640.00000	24.00000	22.95817	640.00000	0.00050	0.00049
645.00000	24.00000	22.95815	645.00000	0.00050	0.00045
650.00000	24.00000	22.95813	650.00000	0.00050	0.00039
652.00000	24.00000	22.95819	652.00000	0.00050	0.00049
655.00000	24.00000	22.95847	655.00000	0.00050	0.00050
663.00000	24.00000	22.95809	663.00000	0.00050	0.00050
665.00000	24.00000	22.95812	665.00000	0.00050	0.00050
666.00000	24.00000	22.95820	666.00000	0.00050	0.00049
667.00000	24.00000	22.95821	667.00000	0.00050	0.00049
672.00000	24.00000	42.84140	672.00000	0.00020	0.00054
673.00000	24.00000	39.85497	673.00000	0.00020	0.00035
676.00000	24.00000	25.48874	676.00000	0.00020	0.00039
678.00000	24.00000	23.21634	678.00000	0.00020	0.00041
679.00000	24.00000	23.17846	679.00000	0.00020	0.00042
680.00000	24.00000	26.24656	680.00000	0.00020	0.00040
681.00000	24.00000	23.23096	681.00000	0.00020	0.00038
682.00000	24.00000	23.75758	682.00000	0.00020	0.00035
683.00000	24.00000	23.80131	683.00000	0.00020	0.00031
686.00000	24.00000	23.82760	686.00000	0.00020	0.00030
699.00000	24.00000	26.69058	699.00000	0.00020	0.00023
702.00000	24.00000	26.69058	702.00000	0.00020	0.00025
705.00000	24.00000	25.74036	705.00000	0.00020	0.00035
713.00000	24.00000	22.95805	713.00000	0.00020	0.00022
715.00000	24.00000	22.95804	715.00000	0.00020	0.00022
717.00000	24.00000	22.95810	717.00000	0.00020	0.00021
724.00000	24.00000	22.92415	724.00000	0.00020	0.00026
725.00000	24.00000	22.75037	725.00000	0.00020	0.00032
729.00000	24.00000	22.95229	729.00000	0.00020	0.00023
735.00000	26.00000	28.47993	735.00000	0.00100	0.00075
737.00000	26.00000	28.29999	737.00000	0.00100	0.00073
738.00000	26.00000	28.78864	738.00000	0.00100	0.00080
745.00000	26.00000	25.85872	745.00000	0.00100	0.00078
748.00000	26.00000	25.06770	748.00000	0.00100	0.00089
749.00000	26.00000	24.07790	749.00000	0.00100	0.00088

752.00000	26.00000	27.63177	752.00000	0.00100	0.00087
755.00000	26.00000	28.67832	755.00000	0.00100	0.00086
762.00000	26.00000	8.43685	762.00000	0.00100	0.00092
767.00000	26.00000	26.68481	767.00000	0.00100	0.00059
768.00000	26.00000	26.33528	768.00000	0.00100	0.00056
769.00000	26.00000	26.68225	769.00000	0.00100	0.00063
771.00000	26.00000	26.69045	771.00000	0.00100	0.00067
772.00000	26.00000	26.69033	772.00000	0.00100	0.00069
776.00000	26.00000	26.69058	776.00000	0.00100	0.00091
783.00000	26.00000	26.69058	783.00000	0.00100	0.00098
786.00000	26.00000	26.69058	786.00000	0.00100	0.00096
787.00000	26.00000	26.69058	787.00000	0.00100	0.00097
790.00000	26.00000	26.69058	790.00000	0.00100	0.00096
793.00000	26.00000	26.69057	793.00000	0.00100	0.00070
800.00000	26.00000	27.01731	800.00000	0.00050	0.00067
806.00000	26.00000	25.54064	806.00000	0.00050	0.00050
821.00000	26.00000	25.98543	821.00000	0.00050	0.00051
822.00000	26.00000	26.38592	822.00000	0.00050	0.00051
823.00000	26.00000	25.97589	823.00000	0.00050	0.00050
826.00000	26.00000	26.50968	826.00000	0.00050	0.00050
829.00000	26.00000	26.65879	829.00000	0.00050	0.00047
835.00000	26.00000	26.69025	835.00000	0.00050	0.00050
840.00000	26.00000	26.69053	840.00000	0.00050	0.00050
841.00000	26.00000	26.69054	841.00000	0.00050	0.00050
844.00000	26.00000	26.69057	844.00000	0.00050	0.00050
853.00000	26.00000	26.69060	853.00000	0.00050	0.00058
855.00000	26.00000	42.84140	855.00000	0.00020	0.00054
856.00000	26.00000	40.79953	856.00000	0.00020	0.00064
857.00000	26.00000	9.93272	857.00000	0.00020	0.00072
868.00000	26.00000	27.44990	868.00000	0.00020	0.00031
870.00000	26.00000	25.60569	870.00000	0.00020	0.00028
879.00000	26.00000	24.35794	879.00000	0.00020	0.00023
881.00000	26.00000	28.42442	881.00000	0.00020	0.00022
884.00000	26.00000	27.30145	884.00000	0.00020	0.00022
885.00000	26.00000	26.66441	885.00000	0.00020	0.00022
887.00000	26.00000	29.25539	887.00000	0.00020	0.00022
889.00000	26.00000	33.60591	889.00000	0.00020	0.00022
892.00000	26.00000	26.85226	892.00000	0.00020	0.00022
893.00000	26.00000	25.93547	893.00000	0.00020	0.00021
895.00000	26.00000	27.89595	895.00000	0.00020	0.00020
896.00000	26.00000	24.76986	896.00000	0.00020	0.00020
898.00000	26.00000	25.84932	898.00000	0.00020	0.00020
906.00000	26.00000	24.44129	906.00000	0.00020	0.00021
907.00000	26.00000	23.96698	907.00000	0.00020	0.00021
908.00000	26.00000	24.14343	908.00000	0.00020	0.00021
909.00000	26.00000	24.35123	909.00000	0.00020	0.00021

Hybrid Metal-KNN-M			Hybrid Metal-RF-C-1		
No.	Ground	Predicted	No.	Ground	Predicted
1.00000	1.00000	2.09524	1.00000	1.00000	4.86282
4.00000	1.00000	2.20000	4.00000	1.00000	2.97720
5.00000	1.00000	1.60000	5.00000	1.00000	2.42997
9.00000	1.00000	1.00000	9.00000	1.00000	1.31920
16.00000	1.00000	1.00000	16.00000	1.00000	3.01980
18.00000	1.00000	1.00000	18.00000	1.00000	2.21597
20.00000	1.00000	1.00000	20.00000	1.00000	1.97553
25.00000	1.00000	1.00000	25.00000	1.00000	3.01723
26.00000	1.00000	1.40000	26.00000	1.00000	2.65510
29.00000	1.00000	1.00000	29.00000	1.00000	2.83873

34.00000	1.00000	1.00000	34.00000	1.00000	1.69287
39.00000	1.00000	1.00000	39.00000	1.00000	1.20880
42.00000	1.00000	1.00000	42.00000	1.00000	1.39767
44.00000	1.00000	1.00000	44.00000	1.00000	1.40430
46.00000	1.00000	1.00000	46.00000	1.00000	1.16487
52.00000	1.00000	1.00000	52.00000	1.00000	1.08323
62.00000	1.00000	2.09524	62.00000	2.00000	4.86282
71.00000	1.00000	1.60000	71.00000	2.00000	2.56357
72.00000	1.00000	1.60000	72.00000	2.00000	2.32600
78.00000	1.00000	1.20000	78.00000	2.00000	3.96987
79.00000	1.00000	1.00000	79.00000	2.00000	4.06337
85.00000	1.00000	1.00000	85.00000	2.00000	2.47010
86.00000	1.00000	1.00000	86.00000	2.00000	2.83753
88.00000	1.00000	1.00000	88.00000	2.00000	2.46257
90.00000	1.00000	1.00000	90.00000	2.00000	2.47100
91.00000	1.00000	1.00000	91.00000	2.00000	2.52363
92.00000	1.00000	1.00000	92.00000	2.00000	2.40683
99.00000	1.00000	1.00000	99.00000	2.00000	2.07247
100.00000	1.00000	1.00000	100.00000	2.00000	2.10413
101.00000	1.00000	1.00000	101.00000	2.00000	2.10197
110.00000	1.00000	1.40000	110.00000	2.00000	2.14730
115.00000	1.00000	1.00000	115.00000	2.00000	2.18120
116.00000	1.00000	1.00000	116.00000	2.00000	2.17920
119.00000	1.00000	1.00000	119.00000	2.00000	2.07120
125.00000	1.00000	1.40000	125.00000	3.00000	4.86647
126.00000	1.00000	1.40000	126.00000	3.00000	3.71700
127.00000	1.00000	1.40000	127.00000	3.00000	3.38197
134.00000	1.00000	1.00000	134.00000	3.00000	3.08940
137.00000	1.00000	1.00000	137.00000	3.00000	3.10390
146.00000	1.00000	1.00000	146.00000	3.00000	3.15417
155.00000	1.00000	1.00000	155.00000	3.00000	3.11827
157.00000	1.00000	1.00000	157.00000	3.00000	3.07903
160.00000	1.00000	1.00000	160.00000	3.00000	3.26737
161.00000	1.00000	1.00000	161.00000	3.00000	3.21610
164.00000	1.00000	1.00000	164.00000	3.00000	2.98737
167.00000	1.00000	1.00000	167.00000	3.00000	3.22577
170.00000	1.00000	1.00000	170.00000	3.00000	3.24347
187.00000	1.00000	1.60000	187.00000	4.00000	7.58397
189.00000	1.00000	1.40000	189.00000	4.00000	6.46273
190.00000	1.00000	1.80000	190.00000	4.00000	5.38160
194.00000	1.00000	1.00000	194.00000	4.00000	4.28103
200.00000	1.00000	1.00000	200.00000	4.00000	4.13490
202.00000	1.00000	1.00000	202.00000	4.00000	4.09400
203.00000	1.00000	1.00000	203.00000	4.00000	4.06887
212.00000	1.00000	1.00000	212.00000	4.00000	4.02007
224.00000	1.00000	1.00000	224.00000	4.00000	3.95213
226.00000	1.00000	1.00000	226.00000	4.00000	3.77793
227.00000	1.00000	1.00000	227.00000	4.00000	3.77740
234.00000	1.00000	1.00000	234.00000	4.00000	3.98960
241.00000	1.00000	1.00000	241.00000	4.00000	4.02893
242.00000	1.00000	1.00000	242.00000	4.00000	4.02893
244.00000	1.00000	1.00000	244.00000	4.00000	4.11583
250.00000	1.00000	1.40000	250.00000	5.00000	5.62457
252.00000	1.00000	1.40000	252.00000	5.00000	5.83483
257.00000	1.00000	1.40000	257.00000	5.00000	6.05717
259.00000	1.00000	1.60000	259.00000	5.00000	5.68890
261.00000	1.00000	1.40000	261.00000	5.00000	5.30557
262.00000	1.00000	1.40000	262.00000	5.00000	5.27327

264.00000	1.00000	1.20000	264.00000	5.00000	5.24723
266.00000	1.00000	1.00000	266.00000	5.00000	4.82720
271.00000	1.00000	1.00000	271.00000	5.00000	5.10573
272.00000	1.00000	1.00000	272.00000	5.00000	5.13993
274.00000	1.00000	1.00000	274.00000	5.00000	4.84443
280.00000	1.00000	1.00000	280.00000	5.00000	5.17453
282.00000	1.00000	1.00000	282.00000	5.00000	5.20207
290.00000	1.00000	1.00000	290.00000	5.00000	5.00267
293.00000	1.00000	1.00000	293.00000	5.00000	5.09007
299.00000	1.00000	1.00000	299.00000	5.00000	4.54143
300.00000	1.00000	1.00000	300.00000	5.00000	4.55643
301.00000	1.00000	1.00000	301.00000	5.00000	4.40043
302.00000	1.00000	1.00000	302.00000	5.00000	4.37517
303.00000	1.00000	1.40000	303.00000	5.00000	4.35293
304.00000	1.00000	1.80000	304.00000	5.00000	4.37593
310.00000	1.00000	1.40000	310.00000	6.00000	5.55280
312.00000	1.00000	1.40000	312.00000	6.00000	5.47290
314.00000	1.00000	1.60000	314.00000	6.00000	4.27133
316.00000	1.00000	1.60000	316.00000	6.00000	5.50663
318.00000	1.00000	1.40000	318.00000	6.00000	5.46807
328.00000	1.00000	1.00000	328.00000	6.00000	5.65107
330.00000	1.00000	1.00000	330.00000	6.00000	5.90347
331.00000	1.00000	1.00000	331.00000	6.00000	5.85380
334.00000	1.00000	1.00000	334.00000	6.00000	6.07963
335.00000	1.00000	1.00000	335.00000	6.00000	6.13723
338.00000	1.00000	1.00000	338.00000	6.00000	6.08813
340.00000	1.00000	1.00000	340.00000	6.00000	6.15630
345.00000	1.00000	1.00000	345.00000	6.00000	5.98150
353.00000	1.00000	1.00000	353.00000	6.00000	6.01050
356.00000	1.00000	1.00000	356.00000	6.00000	5.99400
360.00000	1.00000	1.00000	360.00000	6.00000	5.99670
361.00000	1.00000	1.00000	361.00000	6.00000	5.98370
367.00000	1.00000	2.09524	367.00000	7.00000	4.86282
376.00000	1.00000	1.80000	376.00000	7.00000	6.81020
378.00000	1.00000	1.60000	378.00000	7.00000	6.79293
381.00000	1.00000	1.00000	381.00000	7.00000	6.70237
384.00000	1.00000	1.00000	384.00000	7.00000	6.48180
389.00000	1.00000	1.00000	389.00000	7.00000	4.54423
390.00000	1.00000	1.00000	390.00000	7.00000	4.48330
391.00000	1.00000	1.00000	391.00000	7.00000	4.97630
395.00000	1.00000	1.00000	395.00000	7.00000	6.42927
405.00000	1.00000	1.00000	405.00000	7.00000	6.97480
416.00000	1.00000	1.00000	416.00000	7.00000	7.00150
420.00000	1.00000	1.00000	420.00000	7.00000	6.94200
429.00000	1.00000	2.09524	429.00000	8.00000	7.39884
436.00000	1.00000	1.00000	436.00000	8.00000	7.38460
441.00000	1.00000	1.00000	441.00000	8.00000	7.51630
443.00000	1.00000	1.00000	443.00000	8.00000	6.83310
445.00000	1.00000	1.40000	445.00000	8.00000	7.49557
448.00000	1.00000	1.00000	448.00000	8.00000	7.04813
449.00000	1.00000	1.40000	449.00000	8.00000	7.57613
450.00000	1.00000	1.00000	450.00000	8.00000	7.78330
451.00000	1.00000	1.00000	451.00000	8.00000	7.82767
454.00000	1.00000	1.00000	454.00000	8.00000	7.89643
460.00000	1.00000	1.00000	460.00000	8.00000	7.97817
465.00000	1.00000	1.00000	465.00000	8.00000	8.00200
477.00000	1.00000	1.00000	477.00000	8.00000	7.96700
479.00000	1.00000	1.00000	479.00000	8.00000	7.95840

480.00000	1.00000	1.00000	480.00000	8.00000	7.95640
481.00000	1.00000	1.00000	481.00000	8.00000	7.96640
482.00000	1.00000	1.00000	482.00000	8.00000	7.96640
483.00000	1.00000	1.00000	483.00000	8.00000	7.96357
486.00000	1.00000	1.00000	486.00000	8.00000	7.93457
494.00000	1.00000	2.00000	494.00000	9.00000	6.58283
495.00000	1.00000	2.60000	495.00000	9.00000	6.02900
500.00000	1.00000	1.00000	500.00000	9.00000	8.86913
502.00000	1.00000	1.00000	502.00000	9.00000	8.77177
505.00000	1.00000	1.00000	505.00000	9.00000	8.89050
509.00000	1.00000	1.00000	509.00000	9.00000	8.67050
510.00000	1.00000	1.00000	510.00000	9.00000	8.58887
511.00000	1.00000	1.00000	511.00000	9.00000	8.61053
513.00000	1.00000	1.00000	513.00000	9.00000	8.67707
515.00000	1.00000	1.00000	515.00000	9.00000	8.79500
518.00000	1.00000	1.00000	518.00000	9.00000	8.87900
519.00000	1.00000	1.00000	519.00000	9.00000	8.80600
520.00000	1.00000	1.00000	520.00000	9.00000	8.78667
521.00000	1.00000	1.00000	521.00000	9.00000	8.76267
524.00000	1.00000	1.00000	524.00000	9.00000	8.89750
531.00000	1.00000	1.00000	531.00000	9.00000	8.86797
541.00000	1.00000	1.00000	541.00000	9.00000	8.88970
544.00000	1.00000	1.00000	544.00000	9.00000	8.84160
545.00000	1.00000	1.00000	545.00000	9.00000	8.78760
547.00000	1.00000	1.00000	547.00000	9.00000	8.79810
553.00000	2.00000	1.80000	553.00000	1.00000	6.64602
554.00000	2.00000	2.20000	554.00000	1.00000	7.86107
556.00000	2.00000	2.40000	556.00000	1.00000	4.33880
560.00000	2.00000	2.00000	560.00000	1.00000	2.43537
562.00000	2.00000	1.80000	562.00000	1.00000	2.29897
569.00000	2.00000	1.80000	569.00000	1.00000	1.57257
570.00000	2.00000	1.80000	570.00000	1.00000	1.81310
580.00000	2.00000	2.00000	580.00000	1.00000	1.18973
584.00000	2.00000	2.00000	584.00000	1.00000	1.08227
586.00000	2.00000	2.00000	586.00000	1.00000	1.08240
590.00000	2.00000	2.00000	590.00000	1.00000	1.01000
595.00000	2.00000	2.00000	595.00000	1.00000	1.01100
596.00000	2.00000	2.00000	596.00000	1.00000	1.00000
607.00000	2.00000	2.00000	607.00000	1.00000	1.09440
608.00000	2.00000	2.00000	608.00000	1.00000	1.13220
617.00000	2.00000	1.80000	617.00000	2.00000	2.20407
620.00000	2.00000	1.80000	620.00000	2.00000	2.66643
622.00000	2.00000	2.00000	622.00000	2.00000	2.30097
623.00000	2.00000	1.80000	623.00000	2.00000	2.31777
625.00000	2.00000	2.00000	625.00000	2.00000	2.10317
627.00000	2.00000	2.00000	627.00000	2.00000	2.12883
629.00000	2.00000	2.00000	629.00000	2.00000	2.19750
631.00000	2.00000	2.00000	631.00000	2.00000	2.14663
636.00000	2.00000	2.00000	636.00000	2.00000	2.74383
637.00000	2.00000	2.00000	637.00000	2.00000	3.28190
638.00000	2.00000	1.80000	638.00000	2.00000	4.38860
639.00000	2.00000	2.00000	639.00000	2.00000	2.61463
640.00000	2.00000	2.00000	640.00000	2.00000	2.63397
642.00000	2.00000	1.80000	642.00000	2.00000	2.81513
643.00000	2.00000	1.80000	643.00000	2.00000	2.92300
644.00000	2.00000	1.80000	644.00000	2.00000	2.82900
647.00000	2.00000	1.80000	647.00000	2.00000	2.62403
648.00000	2.00000	1.80000	648.00000	2.00000	2.72800

659.00000	2.00000	2.00000	659.00000	2.00000	2.02517
660.00000	2.00000	2.00000	660.00000	2.00000	2.01167
665.00000	2.00000	2.00000	665.00000	2.00000	2.00867
666.00000	2.00000	2.00000	666.00000	2.00000	2.00067
667.00000	2.00000	2.00000	667.00000	2.00000	2.00267
670.00000	2.00000	2.00000	670.00000	2.00000	2.01987
676.00000	2.00000	1.40000	676.00000	3.00000	4.72643
677.00000	2.00000	1.80000	677.00000	3.00000	4.11033
681.00000	2.00000	2.00000	681.00000	3.00000	4.40300
683.00000	2.00000	2.00000	683.00000	3.00000	3.85567
685.00000	2.00000	2.00000	685.00000	3.00000	3.25673
691.00000	2.00000	2.00000	691.00000	3.00000	3.04357
693.00000	2.00000	2.00000	693.00000	3.00000	3.29463
696.00000	2.00000	2.00000	696.00000	3.00000	2.65003
702.00000	2.00000	2.00000	702.00000	3.00000	3.25747
703.00000	2.00000	2.00000	703.00000	3.00000	3.21820
705.00000	2.00000	2.00000	705.00000	3.00000	3.29513
709.00000	2.00000	2.00000	709.00000	3.00000	3.08033
711.00000	2.00000	2.00000	711.00000	3.00000	3.03777
723.00000	2.00000	2.00000	723.00000	3.00000	3.30607
724.00000	2.00000	2.00000	724.00000	3.00000	3.05827
730.00000	2.00000	2.00000	730.00000	3.00000	3.62140
731.00000	2.00000	2.00000	731.00000	3.00000	3.71357
732.00000	2.00000	2.00000	732.00000	3.00000	3.97900
742.00000	2.00000	1.80000	742.00000	4.00000	4.22780
747.00000	2.00000	2.00000	747.00000	4.00000	4.15390
748.00000	2.00000	2.00000	748.00000	4.00000	4.68777
749.00000	2.00000	2.00000	749.00000	4.00000	4.65397
750.00000	2.00000	2.00000	750.00000	4.00000	4.41393
758.00000	2.00000	2.00000	758.00000	4.00000	4.78403
760.00000	2.00000	2.00000	760.00000	4.00000	3.96370
766.00000	2.00000	2.00000	766.00000	4.00000	3.92327
767.00000	2.00000	2.00000	767.00000	4.00000	3.99017
771.00000	2.00000	2.00000	771.00000	4.00000	4.07467
772.00000	2.00000	2.00000	772.00000	4.00000	4.00960
778.00000	2.00000	2.00000	778.00000	4.00000	4.04920
781.00000	2.00000	2.00000	781.00000	4.00000	4.08423
788.00000	2.00000	2.00000	788.00000	4.00000	4.01900
790.00000	2.00000	2.00000	790.00000	4.00000	4.02500
798.00000	2.00000	1.00000	798.00000	5.00000	4.65060
805.00000	2.00000	2.00000	805.00000	5.00000	5.16663
806.00000	2.00000	2.00000	806.00000	5.00000	5.31293
813.00000	2.00000	2.00000	813.00000	5.00000	5.19937
814.00000	2.00000	2.00000	814.00000	5.00000	5.00827
816.00000	2.00000	2.00000	816.00000	5.00000	4.92543
818.00000	2.00000	2.00000	818.00000	5.00000	4.92827
820.00000	2.00000	2.00000	820.00000	5.00000	5.08803
821.00000	2.00000	2.00000	821.00000	5.00000	5.04803
824.00000	2.00000	2.00000	824.00000	5.00000	5.09783
827.00000	2.00000	2.00000	827.00000	5.00000	4.93660
829.00000	2.00000	2.00000	829.00000	5.00000	5.00723
833.00000	2.00000	2.00000	833.00000	5.00000	5.01800
840.00000	2.00000	2.00000	840.00000	5.00000	5.01200
849.00000	2.00000	2.00000	849.00000	5.00000	5.00950
851.00000	2.00000	2.00000	851.00000	5.00000	5.01350
854.00000	2.00000	2.00000	854.00000	5.00000	4.99550
858.00000	2.00000	2.40000	858.00000	6.00000	6.44850
862.00000	2.00000	2.40000	862.00000	6.00000	5.80647

863.00000	2.00000	2.40000	863.00000	6.00000	5.79550
864.00000	2.00000	2.20000	864.00000	6.00000	5.03780
865.00000	2.00000	2.20000	865.00000	6.00000	5.81550
870.00000	2.00000	2.00000	870.00000	6.00000	5.76720
874.00000	2.00000	2.20000	874.00000	6.00000	5.52277
877.00000	2.00000	2.40000	877.00000	6.00000	4.56880
878.00000	2.00000	2.20000	878.00000	6.00000	4.43060
879.00000	2.00000	2.00000	879.00000	6.00000	4.81940
883.00000	2.00000	2.00000	883.00000	6.00000	5.56403
884.00000	2.00000	2.00000	884.00000	6.00000	5.62740
888.00000	2.00000	2.00000	888.00000	6.00000	5.61700
901.00000	2.00000	1.80000	901.00000	6.00000	5.21907
903.00000	2.00000	2.00000	903.00000	6.00000	5.48240
909.00000	2.00000	2.00000	909.00000	6.00000	5.95247
910.00000	2.00000	2.00000	910.00000	6.00000	5.90407
912.00000	2.00000	2.00000	912.00000	6.00000	5.81267
913.00000	2.00000	2.00000	913.00000	6.00000	5.76917
915.00000	2.00000	2.00000	915.00000	6.00000	5.63377
916.00000	2.00000	2.09524	916.00000	7.00000	4.86282
917.00000	2.00000	1.20000	917.00000	7.00000	5.20888
926.00000	2.00000	2.00000	926.00000	7.00000	6.31093
928.00000	2.00000	2.00000	928.00000	7.00000	6.29647
930.00000	2.00000	2.00000	930.00000	7.00000	5.84230
931.00000	2.00000	2.00000	931.00000	7.00000	5.77797
934.00000	2.00000	2.00000	934.00000	7.00000	6.40323
943.00000	2.00000	2.00000	943.00000	7.00000	6.64427
945.00000	2.00000	2.00000	945.00000	7.00000	6.44767
946.00000	2.00000	2.00000	946.00000	7.00000	6.43127
947.00000	2.00000	2.00000	947.00000	7.00000	6.76467
950.00000	2.00000	2.00000	950.00000	7.00000	6.96250
952.00000	2.00000	2.00000	952.00000	7.00000	6.97000
954.00000	2.00000	2.00000	954.00000	7.00000	6.98600
956.00000	2.00000	2.00000	956.00000	7.00000	6.99200
959.00000	2.00000	2.00000	959.00000	7.00000	6.99600
961.00000	2.00000	2.00000	961.00000	7.00000	7.00000
965.00000	2.00000	2.00000	965.00000	7.00000	7.00000
970.00000	2.00000	2.00000	970.00000	7.00000	6.95800
971.00000	2.00000	2.00000	971.00000	7.00000	6.99550
972.00000	2.00000	2.00000	972.00000	7.00000	6.99950
974.00000	2.00000	2.00000	974.00000	7.00000	7.00050
975.00000	2.00000	2.00000	975.00000	7.00000	6.98200
980.00000	2.00000	1.80000	980.00000	8.00000	6.47443
981.00000	2.00000	2.00000	981.00000	8.00000	6.87260
989.00000	2.00000	2.00000	989.00000	8.00000	7.52697
993.00000	2.00000	2.00000	993.00000	8.00000	7.67257
998.00000	2.00000	2.00000	998.00000	8.00000	7.58443
999.00000	2.00000	2.00000	999.00000	8.00000	7.52393
1005.00000	2.00000	2.00000	1005.00000	8.00000	7.55143
1009.00000	2.00000	2.20000	1009.00000	8.00000	6.78253
1011.00000	2.00000	2.20000	1011.00000	8.00000	6.06163
1012.00000	2.00000	2.00000	1012.00000	8.00000	6.07657
1013.00000	2.00000	2.20000	1013.00000	8.00000	5.85827
1018.00000	2.00000	2.00000	1018.00000	8.00000	7.90137
1021.00000	2.00000	2.00000	1021.00000	8.00000	7.63813
1024.00000	2.00000	2.00000	1024.00000	8.00000	7.97680
1025.00000	2.00000	2.00000	1025.00000	8.00000	7.99080
1029.00000	2.00000	2.00000	1029.00000	8.00000	7.98933
1030.00000	2.00000	2.00000	1030.00000	8.00000	7.95733

1032.00000	2.00000	2.00000	1032.00000	8.00000	7.95723
1033.00000	2.00000	2.00000	1033.00000	8.00000	7.95823
1036.00000	2.00000	2.00000	1036.00000	8.00000	7.87323
1038.00000	2.00000	2.09524	1038.00000	9.00000	4.86282
1040.00000	2.00000	1.20000	1040.00000	9.00000	3.89481
1041.00000	2.00000	1.80000	1041.00000	9.00000	4.84900
1042.00000	2.00000	1.40000	1042.00000	9.00000	5.48063
1046.00000	2.00000	1.40000	1046.00000	9.00000	6.24283
1054.00000	2.00000	1.60000	1054.00000	9.00000	7.77290
1056.00000	2.00000	1.60000	1056.00000	9.00000	7.51530
1058.00000	2.00000	1.80000	1058.00000	9.00000	7.34157
1060.00000	2.00000	1.80000	1060.00000	9.00000	7.37260
1061.00000	2.00000	2.00000	1061.00000	9.00000	7.51397
1064.00000	2.00000	2.00000	1064.00000	9.00000	7.76237
1067.00000	2.00000	1.40000	1067.00000	9.00000	7.36280
1073.00000	2.00000	2.00000	1073.00000	9.00000	8.67130
1075.00000	2.00000	2.00000	1075.00000	9.00000	8.83310
1079.00000	2.00000	2.00000	1079.00000	9.00000	8.82750
1088.00000	2.00000	2.00000	1088.00000	9.00000	8.83967
1100.00000	3.00000	2.00000	1100.00000	1.00000	3.46801
1105.00000	3.00000	3.00000	1105.00000	1.00000	2.37280
1106.00000	3.00000	3.00000	1106.00000	1.00000	3.57000
1111.00000	3.00000	3.00000	1111.00000	1.00000	2.00720
1115.00000	3.00000	3.00000	1115.00000	1.00000	4.27143
1116.00000	3.00000	3.00000	1116.00000	1.00000	4.00567
1118.00000	3.00000	2.20000	1118.00000	1.00000	3.93967
1119.00000	3.00000	2.20000	1119.00000	1.00000	4.41573
1120.00000	3.00000	2.20000	1120.00000	1.00000	5.03300
1123.00000	3.00000	2.60000	1123.00000	1.00000	2.72513
1126.00000	3.00000	2.80000	1126.00000	1.00000	2.86627
1128.00000	3.00000	2.80000	1128.00000	1.00000	1.22650
1131.00000	3.00000	3.00000	1131.00000	1.00000	1.09600
1134.00000	3.00000	3.00000	1134.00000	1.00000	1.00000
1135.00000	3.00000	3.00000	1135.00000	1.00000	1.01400
1138.00000	3.00000	3.00000	1138.00000	1.00000	1.12800
1139.00000	3.00000	3.00000	1139.00000	1.00000	1.12800
1143.00000	3.00000	3.00000	1143.00000	1.00000	1.01600
1149.00000	3.00000	3.00000	1149.00000	1.00000	1.00000
1151.00000	3.00000	3.00000	1151.00000	1.00000	1.00000
1161.00000	3.00000	2.20000	1161.00000	2.00000	3.66140
1166.00000	3.00000	2.66667	1166.00000	2.00000	4.33627
1186.00000	3.00000	3.00000	1186.00000	2.00000	2.23363
1189.00000	3.00000	3.00000	1189.00000	2.00000	2.25853
1190.00000	3.00000	3.00000	1190.00000	2.00000	2.34937
1192.00000	3.00000	3.00000	1192.00000	2.00000	2.24907
1195.00000	3.00000	3.00000	1195.00000	2.00000	2.10820
1200.00000	3.00000	2.20000	1200.00000	2.00000	2.30573
1201.00000	3.00000	2.60000	1201.00000	2.00000	2.31350
1203.00000	3.00000	2.60000	1203.00000	2.00000	2.23527
1207.00000	3.00000	3.00000	1207.00000	2.00000	2.17900
1209.00000	3.00000	3.00000	1209.00000	2.00000	2.04100
1217.00000	3.00000	3.00000	1217.00000	2.00000	2.17573
1218.00000	3.00000	3.00000	1218.00000	2.00000	2.22360
1227.00000	3.00000	2.40000	1227.00000	3.00000	3.38680
1228.00000	3.00000	1.80000	1228.00000	3.00000	3.67487
1229.00000	3.00000	2.20000	1229.00000	3.00000	4.51520
1230.00000	3.00000	3.00000	1230.00000	3.00000	4.10700
1233.00000	3.00000	2.60000	1233.00000	3.00000	4.52170

1234.00000	3.00000	3.00000	1234.00000	3.00000	4.55240
1243.00000	3.00000	3.00000	1243.00000	3.00000	3.82240
1245.00000	3.00000	3.00000	1245.00000	3.00000	3.81523
1246.00000	3.00000	2.60000	1246.00000	3.00000	3.26940
1249.00000	3.00000	3.00000	1249.00000	3.00000	3.33477
1250.00000	3.00000	3.00000	1250.00000	3.00000	3.12880
1254.00000	3.00000	3.00000	1254.00000	3.00000	3.60543
1255.00000	3.00000	3.00000	1255.00000	3.00000	3.83443
1258.00000	3.00000	3.00000	1258.00000	3.00000	3.53623
1259.00000	3.00000	3.00000	1259.00000	3.00000	3.60027
1260.00000	3.00000	1.80000	1260.00000	3.00000	3.46490
1262.00000	3.00000	2.20000	1262.00000	3.00000	3.31927
1265.00000	3.00000	3.00000	1265.00000	3.00000	3.37737
1272.00000	3.00000	3.00000	1272.00000	3.00000	3.20367
1280.00000	3.00000	3.00000	1280.00000	3.00000	4.15063
1281.00000	3.00000	3.00000	1281.00000	3.00000	3.74543
1284.00000	3.00000	2.60000	1284.00000	4.00000	4.20177
1285.00000	3.00000	3.00000	1285.00000	4.00000	4.26953
1286.00000	3.00000	2.80000	1286.00000	4.00000	4.21650
1289.00000	3.00000	3.00000	1289.00000	4.00000	3.89010
1291.00000	3.00000	3.00000	1291.00000	4.00000	4.37413
1296.00000	3.00000	3.00000	1296.00000	4.00000	4.62420
1305.00000	3.00000	3.00000	1305.00000	4.00000	3.79660
1310.00000	3.00000	3.00000	1310.00000	4.00000	4.09800
1318.00000	3.00000	3.00000	1318.00000	4.00000	3.99600
1324.00000	3.00000	3.00000	1324.00000	4.00000	4.03600
1328.00000	3.00000	3.00000	1328.00000	4.00000	4.01600
1331.00000	3.00000	3.00000	1331.00000	4.00000	3.99800
1332.00000	3.00000	3.00000	1332.00000	4.00000	3.98800
1334.00000	3.00000	3.00000	1334.00000	4.00000	3.98450
1335.00000	3.00000	3.00000	1335.00000	4.00000	3.98450
1336.00000	3.00000	3.00000	1336.00000	4.00000	3.98450
1346.00000	3.00000	3.00000	1346.00000	5.00000	4.55070
1347.00000	3.00000	3.00000	1347.00000	5.00000	4.68487
1354.00000	3.00000	3.00000	1354.00000	5.00000	4.08477
1356.00000	3.00000	3.00000	1356.00000	5.00000	5.12507
1357.00000	3.00000	3.00000	1357.00000	5.00000	4.74000
1362.00000	3.00000	3.00000	1362.00000	5.00000	5.03743
1373.00000	3.00000	3.00000	1373.00000	5.00000	5.26830
1374.00000	3.00000	3.00000	1374.00000	5.00000	5.19793
1378.00000	3.00000	2.20000	1378.00000	5.00000	4.77890
1379.00000	3.00000	2.20000	1379.00000	5.00000	4.70427
1381.00000	3.00000	2.60000	1381.00000	5.00000	5.59430
1384.00000	3.00000	3.00000	1384.00000	5.00000	4.89350
1385.00000	3.00000	3.00000	1385.00000	5.00000	4.95643
1389.00000	3.00000	3.00000	1389.00000	5.00000	5.01530
1390.00000	3.00000	3.00000	1390.00000	5.00000	5.07557
1392.00000	3.00000	3.00000	1392.00000	5.00000	5.08957
1395.00000	3.00000	3.00000	1395.00000	5.00000	5.02480
1397.00000	3.00000	3.00000	1397.00000	5.00000	5.01350
1405.00000	3.00000	1.40000	1405.00000	6.00000	2.09616
1406.00000	3.00000	1.80000	1406.00000	6.00000	2.86727
1411.00000	3.00000	3.00000	1411.00000	6.00000	4.96076
1414.00000	3.00000	3.00000	1414.00000	6.00000	5.82807
1420.00000	3.00000	3.00000	1420.00000	6.00000	5.93207
1421.00000	3.00000	3.00000	1421.00000	6.00000	5.89100
1423.00000	3.00000	3.00000	1423.00000	6.00000	5.97140
1425.00000	3.00000	3.00000	1425.00000	6.00000	5.54403

1427.00000	3.00000	3.00000	1427.00000	6.00000	5.90410
1429.00000	3.00000	3.00000	1429.00000	6.00000	5.80360
1431.00000	3.00000	3.00000	1431.00000	6.00000	5.88933
1433.00000	3.00000	3.00000	1433.00000	6.00000	5.68337
1434.00000	3.00000	3.00000	1434.00000	6.00000	5.72607
1435.00000	3.00000	3.00000	1435.00000	6.00000	5.83913
1436.00000	3.00000	3.00000	1436.00000	6.00000	5.89837
1441.00000	3.00000	3.00000	1441.00000	6.00000	6.02720
1442.00000	3.00000	3.00000	1442.00000	6.00000	5.93593
1445.00000	3.00000	2.60000	1445.00000	6.00000	5.96707
1450.00000	3.00000	3.00000	1450.00000	6.00000	6.06083
1451.00000	3.00000	3.00000	1451.00000	6.00000	6.00697
1455.00000	3.00000	3.00000	1455.00000	6.00000	5.96967
1457.00000	3.00000	3.00000	1457.00000	6.00000	6.07320
1458.00000	3.00000	3.00000	1458.00000	6.00000	5.98120
1460.00000	3.00000	3.00000	1460.00000	6.00000	6.14727
1463.00000	3.00000	3.00000	1463.00000	6.00000	6.01487
1465.00000	3.00000	2.09524	1465.00000	7.00000	4.86282
1466.00000	3.00000	1.80000	1466.00000	7.00000	6.65597
1470.00000	3.00000	3.00000	1470.00000	7.00000	6.86803
1471.00000	3.00000	3.00000	1471.00000	7.00000	6.84633
1478.00000	3.00000	3.00000	1478.00000	7.00000	6.40023
1481.00000	3.00000	3.00000	1481.00000	7.00000	6.88737
1483.00000	3.00000	3.00000	1483.00000	7.00000	6.80250
1484.00000	3.00000	3.00000	1484.00000	7.00000	6.77500
1488.00000	3.00000	3.00000	1488.00000	7.00000	7.03860
1493.00000	3.00000	3.00000	1493.00000	7.00000	6.98300
1495.00000	3.00000	3.00000	1495.00000	7.00000	6.85080
1496.00000	3.00000	3.00000	1496.00000	7.00000	6.89340
1498.00000	3.00000	3.00000	1498.00000	7.00000	6.93660
1500.00000	3.00000	3.00000	1500.00000	7.00000	6.94357
1504.00000	3.00000	3.00000	1504.00000	7.00000	6.99753
1515.00000	3.00000	3.00000	1515.00000	7.00000	6.97703
1516.00000	3.00000	3.00000	1516.00000	7.00000	6.96503
1520.00000	3.00000	3.00000	1520.00000	7.00000	6.91567
1528.00000	3.00000	1.60000	1528.00000	8.00000	5.43138
1530.00000	3.00000	1.80000	1530.00000	8.00000	5.48005
1531.00000	3.00000	1.60000	1531.00000	8.00000	4.89957
1534.00000	3.00000	2.20000	1534.00000	8.00000	4.70324
1537.00000	3.00000	2.20000	1537.00000	8.00000	7.29620
1538.00000	3.00000	2.20000	1538.00000	8.00000	7.09593
1540.00000	3.00000	3.00000	1540.00000	8.00000	7.49740
1545.00000	3.00000	3.00000	1545.00000	8.00000	7.19787
1548.00000	3.00000	3.00000	1548.00000	8.00000	7.49317
1551.00000	3.00000	1.80000	1551.00000	8.00000	5.17513
1553.00000	3.00000	3.00000	1553.00000	8.00000	6.05050
1554.00000	3.00000	3.00000	1554.00000	8.00000	6.06420
1558.00000	3.00000	3.00000	1558.00000	8.00000	6.80240
1559.00000	3.00000	3.00000	1559.00000	8.00000	6.53757
1560.00000	3.00000	3.00000	1560.00000	8.00000	6.54003
1567.00000	3.00000	3.00000	1567.00000	8.00000	7.74037
1568.00000	3.00000	3.00000	1568.00000	8.00000	7.65620
1571.00000	3.00000	3.00000	1571.00000	8.00000	7.50940
1573.00000	3.00000	3.00000	1573.00000	8.00000	7.48887
1575.00000	3.00000	3.00000	1575.00000	8.00000	7.77073
1580.00000	3.00000	3.00000	1580.00000	8.00000	7.84523
1584.00000	3.00000	3.00000	1584.00000	8.00000	7.78590
1585.00000	3.00000	3.00000	1585.00000	8.00000	7.66863

1588.00000	3.00000	2.20000	1588.00000	9.00000	7.31181
1596.00000	3.00000	3.00000	1596.00000	9.00000	8.30813
1598.00000	3.00000	3.00000	1598.00000	9.00000	8.47917
1602.00000	3.00000	3.00000	1602.00000	9.00000	8.77900
1603.00000	3.00000	3.00000	1603.00000	9.00000	8.78500
1607.00000	3.00000	3.00000	1607.00000	9.00000	8.82707
1611.00000	3.00000	3.00000	1611.00000	9.00000	8.47730
1612.00000	3.00000	3.00000	1612.00000	9.00000	8.46317
1615.00000	3.00000	3.00000	1615.00000	9.00000	8.54637
1616.00000	3.00000	3.00000	1616.00000	9.00000	8.32547
1619.00000	3.00000	3.00000	1619.00000	9.00000	8.52837
1620.00000	3.00000	3.00000	1620.00000	9.00000	8.41290
1621.00000	3.00000	3.00000	1621.00000	9.00000	8.35490
1624.00000	3.00000	3.00000	1624.00000	9.00000	8.44403
1632.00000	3.00000	3.00000	1632.00000	9.00000	8.95410
1634.00000	3.00000	3.00000	1634.00000	9.00000	8.97693
1637.00000	3.00000	3.00000	1637.00000	9.00000	8.98040
1642.00000	3.00000	3.00000	1642.00000	9.00000	8.90497
1644.00000	3.00000	3.00000	1644.00000	9.00000	8.58623
1647.00000	3.00000	3.00000	1647.00000	9.00000	7.96213

Hybrid Metal-KNN-C-2

No.	Ground	Predicted
1.00000	1.00000	5.83333
4.00000	1.00000	4.20000
5.00000	1.00000	2.40000
9.00000	1.00000	1.00000
16.00000	1.00000	1.00000
18.00000	1.00000	3.40000
20.00000	1.00000	2.20000
25.00000	1.00000	2.20000
26.00000	1.00000	2.20000
29.00000	1.00000	1.00000
34.00000	1.00000	1.00000
39.00000	1.00000	1.00000
42.00000	1.00000	1.00000
44.00000	1.00000	1.00000
46.00000	1.00000	1.00000
52.00000	1.00000	1.00000
62.00000	2.00000	5.83333
71.00000	2.00000	3.40000
72.00000	2.00000	3.40000
78.00000	2.00000	2.00000
79.00000	2.00000	2.00000
85.00000	2.00000	2.00000
86.00000	2.00000	2.00000
88.00000	2.00000	2.00000
90.00000	2.00000	2.00000
91.00000	2.00000	2.00000
92.00000	2.00000	2.00000
99.00000	2.00000	2.00000
100.00000	2.00000	2.00000
101.00000	2.00000	2.00000
110.00000	2.00000	2.00000
115.00000	2.00000	2.00000
116.00000	2.00000	2.00000
119.00000	2.00000	2.00000
125.00000	3.00000	5.20000
126.00000	3.00000	5.40000

127.00000	3.00000	5.00000
134.00000	3.00000	3.00000
137.00000	3.00000	3.00000
146.00000	3.00000	3.00000
155.00000	3.00000	3.00000
157.00000	3.00000	3.00000
160.00000	3.00000	3.00000
161.00000	3.00000	3.00000
164.00000	3.00000	3.00000
167.00000	3.00000	3.00000
170.00000	3.00000	3.00000
187.00000	4.00000	6.20000
189.00000	4.00000	5.60000
190.00000	4.00000	4.80000
194.00000	4.00000	4.00000
200.00000	4.00000	4.00000
202.00000	4.00000	4.00000
203.00000	4.00000	4.00000
212.00000	4.00000	4.00000
224.00000	4.00000	4.00000
226.00000	4.00000	4.00000
227.00000	4.00000	4.00000
234.00000	4.00000	4.00000
241.00000	4.00000	4.00000
242.00000	4.00000	4.00000
244.00000	4.00000	4.00000
250.00000	5.00000	4.00000
252.00000	5.00000	4.60000
257.00000	5.00000	5.00000
259.00000	5.00000	5.00000
261.00000	5.00000	5.00000
262.00000	5.00000	5.00000
264.00000	5.00000	5.00000
266.00000	5.00000	5.00000
271.00000	5.00000	5.00000
272.00000	5.00000	5.00000
274.00000	5.00000	5.00000
280.00000	5.00000	5.00000
282.00000	5.00000	5.00000
290.00000	5.00000	5.00000
293.00000	5.00000	5.00000
299.00000	5.00000	5.00000
300.00000	5.00000	5.00000
301.00000	5.00000	5.00000
302.00000	5.00000	5.00000
303.00000	5.00000	5.00000
304.00000	5.00000	5.00000
310.00000	6.00000	3.20000
312.00000	6.00000	3.80000
314.00000	6.00000	4.60000
316.00000	6.00000	5.60000
318.00000	6.00000	6.00000
328.00000	6.00000	6.00000
330.00000	6.00000	6.00000
331.00000	6.00000	6.00000
334.00000	6.00000	6.00000
335.00000	6.00000	6.00000
338.00000	6.00000	6.00000

340.00000	6.00000	6.00000
345.00000	6.00000	6.00000
353.00000	6.00000	6.00000
356.00000	6.00000	6.00000
360.00000	6.00000	6.00000
361.00000	6.00000	6.00000
367.00000	7.00000	5.83333
376.00000	7.00000	6.80000
378.00000	7.00000	7.00000
381.00000	7.00000	7.00000
384.00000	7.00000	7.00000
389.00000	7.00000	5.80000
390.00000	7.00000	5.80000
391.00000	7.00000	5.80000
395.00000	7.00000	7.00000
405.00000	7.00000	7.00000
416.00000	7.00000	7.00000
420.00000	7.00000	7.00000
429.00000	8.00000	5.83333
436.00000	8.00000	8.00000
441.00000	8.00000	8.00000
443.00000	8.00000	8.00000
445.00000	8.00000	8.00000
448.00000	8.00000	8.00000
449.00000	8.00000	8.00000
450.00000	8.00000	8.00000
451.00000	8.00000	8.00000
454.00000	8.00000	8.00000
460.00000	8.00000	8.00000
465.00000	8.00000	8.00000
477.00000	8.00000	8.00000
479.00000	8.00000	8.00000
480.00000	8.00000	8.00000
481.00000	8.00000	8.00000
482.00000	8.00000	8.00000
483.00000	8.00000	8.00000
486.00000	8.00000	8.00000
494.00000	9.00000	6.40000
495.00000	9.00000	7.60000
500.00000	9.00000	9.00000
502.00000	9.00000	9.00000
505.00000	9.00000	9.00000
509.00000	9.00000	9.00000
510.00000	9.00000	9.00000
511.00000	9.00000	9.00000
513.00000	9.00000	9.00000
515.00000	9.00000	9.00000
518.00000	9.00000	9.00000
519.00000	9.00000	9.00000
520.00000	9.00000	9.00000
521.00000	9.00000	9.00000
524.00000	9.00000	9.00000
531.00000	9.00000	9.00000
541.00000	9.00000	9.00000
544.00000	9.00000	9.00000
545.00000	9.00000	9.00000
547.00000	9.00000	9.00000
553.00000	1.00000	3.80000

554.00000	1.00000	4.00000
556.00000	1.00000	2.60000
560.00000	1.00000	2.20000
562.00000	1.00000	1.80000
569.00000	1.00000	1.00000
570.00000	1.00000	1.00000
580.00000	1.00000	1.00000
584.00000	1.00000	1.00000
586.00000	1.00000	1.00000
590.00000	1.00000	1.00000
595.00000	1.00000	1.00000
596.00000	1.00000	1.00000
607.00000	1.00000	1.00000
608.00000	1.00000	1.00000
617.00000	2.00000	3.40000
620.00000	2.00000	2.00000
622.00000	2.00000	2.00000
623.00000	2.00000	2.00000
625.00000	2.00000	2.00000
627.00000	2.00000	2.00000
629.00000	2.00000	2.00000
631.00000	2.00000	2.00000
636.00000	2.00000	2.00000
637.00000	2.00000	2.00000
638.00000	2.00000	2.00000
639.00000	2.00000	2.00000
640.00000	2.00000	2.00000
642.00000	2.00000	2.00000
643.00000	2.00000	2.00000
644.00000	2.00000	2.00000
647.00000	2.00000	2.00000
648.00000	2.00000	2.00000
659.00000	2.00000	2.00000
660.00000	2.00000	2.00000
665.00000	2.00000	2.00000
666.00000	2.00000	2.00000
667.00000	2.00000	2.00000
670.00000	2.00000	2.00000
676.00000	3.00000	4.00000
677.00000	3.00000	4.20000
681.00000	3.00000	1.80000
683.00000	3.00000	2.20000
685.00000	3.00000	2.60000
691.00000	3.00000	3.00000
693.00000	3.00000	3.20000
696.00000	3.00000	2.20000
702.00000	3.00000	3.20000
703.00000	3.00000	3.00000
705.00000	3.00000	3.00000
709.00000	3.00000	3.00000
711.00000	3.00000	3.00000
723.00000	3.00000	3.00000
724.00000	3.00000	3.00000
730.00000	3.00000	3.00000
731.00000	3.00000	3.00000
732.00000	3.00000	3.00000
742.00000	4.00000	4.00000
747.00000	4.00000	3.80000

748.00000	4.00000	3.80000
749.00000	4.00000	3.60000
750.00000	4.00000	3.60000
758.00000	4.00000	3.20000
760.00000	4.00000	4.00000
766.00000	4.00000	4.00000
767.00000	4.00000	4.00000
771.00000	4.00000	4.00000
772.00000	4.00000	4.00000
778.00000	4.00000	4.00000
781.00000	4.00000	4.00000
788.00000	4.00000	4.00000
790.00000	4.00000	4.00000
798.00000	5.00000	7.20000
805.00000	5.00000	5.80000
806.00000	5.00000	5.80000
813.00000	5.00000	5.00000
814.00000	5.00000	5.00000
816.00000	5.00000	5.00000
818.00000	5.00000	5.00000
820.00000	5.00000	5.00000
821.00000	5.00000	5.00000
824.00000	5.00000	5.00000
827.00000	5.00000	5.00000
829.00000	5.00000	5.00000
833.00000	5.00000	5.00000
840.00000	5.00000	5.00000
849.00000	5.00000	5.00000
851.00000	5.00000	5.00000
854.00000	5.00000	5.00000
858.00000	6.00000	6.00000
862.00000	6.00000	6.00000
863.00000	6.00000	6.00000
864.00000	6.00000	6.00000
865.00000	6.00000	6.00000
870.00000	6.00000	6.00000
874.00000	6.00000	6.00000
877.00000	6.00000	6.00000
878.00000	6.00000	6.00000
879.00000	6.00000	6.00000
883.00000	6.00000	6.00000
884.00000	6.00000	6.00000
888.00000	6.00000	6.00000
901.00000	6.00000	6.00000
903.00000	6.00000	6.00000
909.00000	6.00000	6.00000
910.00000	6.00000	6.00000
912.00000	6.00000	6.00000
913.00000	6.00000	6.00000
915.00000	6.00000	6.00000
916.00000	7.00000	4.14286
917.00000	7.00000	4.40000
926.00000	7.00000	6.60000
928.00000	7.00000	6.20000
930.00000	7.00000	5.80000
931.00000	7.00000	5.80000
934.00000	7.00000	6.20000
943.00000	7.00000	7.00000

945.00000	7.00000	7.00000
946.00000	7.00000	7.00000
947.00000	7.00000	7.00000
950.00000	7.00000	7.00000
952.00000	7.00000	7.00000
954.00000	7.00000	7.00000
956.00000	7.00000	7.00000
959.00000	7.00000	7.00000
961.00000	7.00000	7.00000
965.00000	7.00000	7.00000
970.00000	7.00000	7.00000
971.00000	7.00000	7.00000
972.00000	7.00000	7.00000
974.00000	7.00000	7.00000
975.00000	7.00000	7.00000
980.00000	8.00000	5.60000
981.00000	8.00000	5.60000
989.00000	8.00000	8.00000
993.00000	8.00000	8.00000
998.00000	8.00000	8.00000
999.00000	8.00000	8.00000
1005.00000	8.00000	8.00000
1009.00000	8.00000	8.00000
1011.00000	8.00000	8.00000
1012.00000	8.00000	8.00000
1013.00000	8.00000	8.00000
1018.00000	8.00000	8.00000
1021.00000	8.00000	8.00000
1024.00000	8.00000	8.00000
1025.00000	8.00000	8.00000
1029.00000	8.00000	8.00000
1030.00000	8.00000	8.00000
1032.00000	8.00000	8.00000
1033.00000	8.00000	8.00000
1036.00000	8.00000	8.00000
1038.00000	9.00000	4.14286
1040.00000	9.00000	3.20000
1041.00000	9.00000	4.40000
1042.00000	9.00000	3.80000
1046.00000	9.00000	7.80000
1054.00000	9.00000	8.20000
1056.00000	9.00000	8.20000
1058.00000	9.00000	9.00000
1060.00000	9.00000	9.00000
1061.00000	9.00000	9.00000
1064.00000	9.00000	9.00000
1067.00000	9.00000	6.20000
1073.00000	9.00000	9.00000
1075.00000	9.00000	9.00000
1079.00000	9.00000	9.00000
1088.00000	9.00000	9.00000
1100.00000	1.00000	4.40000
1105.00000	1.00000	3.00000
1106.00000	1.00000	3.80000
1111.00000	1.00000	1.80000
1115.00000	1.00000	3.40000
1116.00000	1.00000	3.40000
1118.00000	1.00000	4.20000

1119.00000	1.00000	4.20000
1120.00000	1.00000	3.40000
1123.00000	1.00000	1.80000
1126.00000	1.00000	1.80000
1128.00000	1.00000	1.00000
1131.00000	1.00000	1.00000
1134.00000	1.00000	1.00000
1135.00000	1.00000	1.00000
1138.00000	1.00000	1.00000
1139.00000	1.00000	1.00000
1143.00000	1.00000	1.00000
1149.00000	1.00000	1.00000
1151.00000	1.00000	1.00000
1161.00000	2.00000	6.40000
1166.00000	2.00000	2.00000
1186.00000	2.00000	2.00000
1189.00000	2.00000	2.00000
1190.00000	2.00000	2.00000
1192.00000	2.00000	2.00000
1195.00000	2.00000	2.00000
1200.00000	2.00000	2.00000
1201.00000	2.00000	2.00000
1203.00000	2.00000	2.00000
1207.00000	2.00000	2.00000
1209.00000	2.00000	2.00000
1217.00000	2.00000	2.00000
1218.00000	2.00000	2.00000
1227.00000	3.00000	6.20000
1228.00000	3.00000	6.80000
1229.00000	3.00000	5.20000
1230.00000	3.00000	5.00000
1233.00000	3.00000	3.00000
1234.00000	3.00000	3.00000
1243.00000	3.00000	3.00000
1245.00000	3.00000	3.00000
1246.00000	3.00000	3.00000
1249.00000	3.00000	3.00000
1250.00000	3.00000	3.00000
1254.00000	3.00000	3.00000
1255.00000	3.00000	3.00000
1258.00000	3.00000	3.00000
1259.00000	3.00000	3.00000
1260.00000	3.00000	2.80000
1262.00000	3.00000	2.60000
1265.00000	3.00000	3.00000
1272.00000	3.00000	3.00000
1280.00000	3.00000	3.00000
1281.00000	3.00000	3.00000
1284.00000	4.00000	3.20000
1285.00000	4.00000	3.20000
1286.00000	4.00000	3.20000
1289.00000	4.00000	3.60000
1291.00000	4.00000	4.00000
1296.00000	4.00000	4.00000
1305.00000	4.00000	4.00000
1310.00000	4.00000	4.00000
1318.00000	4.00000	4.00000
1324.00000	4.00000	4.00000

1328.00000	4.00000	4.00000
1331.00000	4.00000	4.00000
1332.00000	4.00000	4.00000
1334.00000	4.00000	4.00000
1335.00000	4.00000	4.00000
1336.00000	4.00000	4.00000
1346.00000	5.00000	4.20000
1347.00000	5.00000	5.20000
1354.00000	5.00000	2.60000
1356.00000	5.00000	3.60000
1357.00000	5.00000	4.60000
1362.00000	5.00000	5.20000
1373.00000	5.00000	5.00000
1374.00000	5.00000	5.20000
1378.00000	5.00000	4.40000
1379.00000	5.00000	3.60000
1381.00000	5.00000	5.00000
1384.00000	5.00000	5.00000
1385.00000	5.00000	5.00000
1389.00000	5.00000	5.00000
1390.00000	5.00000	5.00000
1392.00000	5.00000	5.00000
1395.00000	5.00000	5.00000
1397.00000	5.00000	5.00000
1405.00000	6.00000	5.40000
1406.00000	6.00000	3.60000
1411.00000	6.00000	3.80000
1414.00000	6.00000	6.00000
1420.00000	6.00000	5.60000
1421.00000	6.00000	5.60000
1423.00000	6.00000	5.40000
1425.00000	6.00000	5.40000
1427.00000	6.00000	5.40000
1429.00000	6.00000	5.60000
1431.00000	6.00000	5.40000
1433.00000	6.00000	5.40000
1434.00000	6.00000	5.80000
1435.00000	6.00000	5.80000
1436.00000	6.00000	6.00000
1441.00000	6.00000	6.00000
1442.00000	6.00000	6.00000
1445.00000	6.00000	6.00000
1450.00000	6.00000	6.00000
1451.00000	6.00000	6.00000
1455.00000	6.00000	6.00000
1457.00000	6.00000	6.00000
1458.00000	6.00000	6.00000
1460.00000	6.00000	6.00000
1463.00000	6.00000	6.00000
1465.00000	7.00000	4.75000
1466.00000	7.00000	8.20000
1470.00000	7.00000	7.00000
1471.00000	7.00000	7.00000
1478.00000	7.00000	7.00000
1481.00000	7.00000	7.00000
1483.00000	7.00000	7.00000
1484.00000	7.00000	7.00000
1488.00000	7.00000	7.00000

1493.00000	7.00000	6.00000
1495.00000	7.00000	7.00000
1496.00000	7.00000	7.00000
1498.00000	7.00000	7.00000
1500.00000	7.00000	7.00000
1504.00000	7.00000	7.00000
1515.00000	7.00000	7.00000
1516.00000	7.00000	7.00000
1520.00000	7.00000	7.00000
1528.00000	8.00000	5.40000
1530.00000	8.00000	5.00000
1531.00000	8.00000	6.00000
1534.00000	8.00000	7.60000
1537.00000	8.00000	8.00000
1538.00000	8.00000	8.00000
1540.00000	8.00000	7.00000
1545.00000	8.00000	8.00000
1548.00000	8.00000	8.00000
1551.00000	8.00000	4.40000
1553.00000	8.00000	6.80000
1554.00000	8.00000	8.00000
1558.00000	8.00000	8.00000
1559.00000	8.00000	8.00000
1560.00000	8.00000	8.00000
1567.00000	8.00000	8.00000
1568.00000	8.00000	8.00000
1571.00000	8.00000	8.00000
1573.00000	8.00000	8.00000
1575.00000	8.00000	8.00000
1580.00000	8.00000	8.00000
1584.00000	8.00000	8.00000
1585.00000	8.00000	8.00000
1588.00000	9.00000	5.50000
1596.00000	9.00000	9.00000
1598.00000	9.00000	9.00000
1602.00000	9.00000	9.00000
1603.00000	9.00000	9.00000
1607.00000	9.00000	9.00000
1611.00000	9.00000	9.00000
1612.00000	9.00000	9.00000
1615.00000	9.00000	9.00000
1616.00000	9.00000	9.00000
1619.00000	9.00000	9.00000
1620.00000	9.00000	9.00000
1621.00000	9.00000	9.00000
1624.00000	9.00000	8.80000
1632.00000	9.00000	9.00000
1634.00000	9.00000	9.00000
1637.00000	9.00000	9.00000
1642.00000	9.00000	9.00000
1644.00000	9.00000	9.00000
1647.00000	9.00000	9.00000
